# Structure of NHE6 and its lipid-mediated interactions regulating endosomal pH

**DOI:** 10.1038/s41467-026-75877-x

**Published:** 2026-08-11

**Authors:** Sukkyeong Jung, Hyunku Yeo, Hang Li, Surabhi Kokane, Tom Reichenbach, Ashutosh Gulati, Giuseppe Albano, Carla Kirschbaum, Tin Manh Ho, Michael Landreh, Mia Abramsson, Carol V. Robinson, Daniel G. Fuster, David Drew

**Affiliations:** 1 Department of Biochemistry and Biophysics, Science for Life laboratory, Stockholm University, Stockholm, Sweden; 2 Department of Nephrology and Hypertension, Inselspital, Bern University Hospital and University of Bern, Bern, Switzerland; 3 Department of Chemistry, Dorothy Crowfoot Hodgkin Building, University of Oxford, Oxford, UK; 4 Department of Cell and Molecular Biology, Uppsala University, Uppsala, Sweden

**Keywords:** Ion transport, Cryoelectron microscopy, Neuronal development, Membrane lipids

## Abstract

Sodium-proton exchangers (NHEs) are found in all cells to regulate intracellular pH, sodium levels and cell volume. In humans, there are nine different NHE transporters (SLC9A1-9), which vary in tissue distribution, kinetics and regulation. NHE6 localizes to endosomal membranes and mutations in the protein are known to cause the X-linked neurological disorder Christianson syndrome. Despite its importance, the structural basis of NHE6 function and regulation is unclear. Here we report four cryo-electron microscopy structures of rat NHE6 between 2.2 and 3.3 Å resolution, revealing its homodimeric structure, ion binding and remodelling by lipids. We characterize a lipid-binding site between the protomers that accommodates the endosomal-specific phosphatidylinositol 3-phosphate (PI3P) lipid. Using solid-supported membrane (SSM)-based electrophysiology we demonstrate that NHE6 transports both Na^+^ and K^+^ ions and that PI3P enhances NHE6 stability and activity. Furthermore, we identify a phosphatidylinositol 4,5-bisphosphate (PI(4,5)P_2_) lipid, which interacts with the C-terminal domain of NHE6 to stabilize an auto-inhibited state. We further demonstrate that NHE6 is non-functional when mislocalized to the plasma membrane where PI(4,5)P_2_ is primarily located. We propose the lipid-dependent regulation has evolved to shut-down NHE6 activity during recycling of endosomes at the plasma membrane.

## Abstract (web summary)

The sodium-proton exchanger NHE6 localises to endosomal membranes and mutations in the protein are known to cause the X-linked neurological disorder Christianson syndrome. Here the authors report four cryo-EM structures of rat NHE6, revealing its homodimeric structure, ion binding and remodelling by lipids.

## Introduction

Na^+^/H^+^ exchangers (NHEs) facilitate the exchange of cations (Na^+^/Li^+^/K^+^) and protons (H^+^) across membranes to regulate intracellular pH, sodium levels and cell volume^1^. In mammals, thirteen distinct Na⁺/H⁺ exchangers have been identified that can be classified into three subfamilies: SLC9A (NHE1–NHE9), SLC9B (NHA1–NHA2), and the sperm-specific SLC9C1-2^1,2^. NHEs vary in their substrate specificity, transport kinetics, subcellular localization, and tissue expression^1^. Isoforms NHE1 to NHE5 are predominantly expressed at the plasma membrane, where they play essential roles in maintaining acid-base homeostasis^1^. In contrast, isoforms NHE6 to NHE9 are principally localized to organelles, where they extrude H^+^ to counterbalance V-ATPase activity and thereby fine-tune organellar pH^3^.

NHE6, encoded by the *SLC9A6* gene, regulates endosomal pH by exchanging endosomal H^+^ for cytoplasmic Na^+^ or K^+^^3^. NHE6 is essential for proper endosomal receptor recycling^4^. NHE6 is critical for maintaining brain functions^5–7^, as its activity is connected to several neuronal processes^3,8–11^. In synapses, NHE6 plays a vital role in synaptic vesicle function and plasticity^9^. During NMDA receptor-dependent long-term potentiation, NHE6-containing vesicles exhibit enhanced translocation to dendrite ends, suggesting a role in learning and memory^9^. Furthermore, NHE6 interacts with SCAMP5^12,13^, and this interaction is thought to be important for regulating presynaptic efficacy. Loss-of-function mutations in NHE6 further lead to Christianson syndrome^4,7,8,14,15^, a severe X-linked developmental disorder characterized by intellectual disability, autism, ataxia, and epilepsy. In essence, NHE6 dysfunction causes endosomal hyperacidification, which impairs endosome maturation, trafficking, and lysosome function^15,16^. This disruption impacts synaptic function, contributing to both the observed neurodevelopmental and neurodegenerative phenotypes. Interestingly, NHE6 has also been implicated in Alzheimer’s disease pathology^11^, with its depletion shown to correct ApoE4-mediated synaptic impairments^17^.

Na^+^/H^+^ exchangers are functional homodimers made up of a 6-TM core ion translocation domain and a dimerization (scaffold) domain^18–24^. They operate by an elevator alternating-access mechanism, whereby either H^+^ or Na^+^ (K^+^) bind to a strictly conserved ion-binding aspartate in the core domain and is subsequently carried across the membrane against the fixed dimer domain^18,19,25^. However, despite extensive biochemical, computational and biophysical studies, ion-bound structures have yet to be determined, with the exception of a distantly-related bacterial K^+^/H^+^ exchanger from *E. coli*^26^. In addition to its ion-binding site architecture, NHE activity is further allosterically regulated by the binding of specific lipids and by their long, extramembrane globular C-terminal domain (CTD)^1,27^.

Whilst the structure of the core ion translocation domain is well-conserved^19^, the scaffold domain shows much greater variability, and this alters how the protomers come together. Essentially, either weaker or more dynamic dimerization interfaces appear to require the binding of specific lipids to stabilise the homodimer assembly, acting as a molecular glue between protomers: cardiolipin with NhaA^28–30^, phosphatidic acid (PA) with SLC9C1^23^, phosphatidylinositol (PI) and PA with SLC9B2^22,31^ and phosphatidylinositol 3,5-bisphosphate [PI(3,5)P_2_] with NHE9^32^. The proposed model is that these ligand-like lipid interactions are formed under cell specific cues to regulate NHE activity via promoting homodimerization stability. The C-terminal domain (CTD), on the other hand, is thought to inactivate the transporter by directly restricting the mobility of the core domain^32^, unless detached following its binding to other auxiliary proteins, e.g., CHP1^20^. Although it is unclear exactly how lipids and CTD interactions fine-tune NHE activity, it is apparent that these proteins are some of the most highly regulated transporters, mirroring the exquisite regulation of intracellular pH^33^.

Given the importance of NHE6 to not only brain function, but to the maintenance of endosomal pH in general, here, we set out to determine the molecular basis of NHE6 function and regulation.

## Results

### Cryo EM structure of the rat NHE6 in nanodiscs and detergent

Rat NHE6 was expressed by transient transfection in mammalian HEK293F cells and purified in lauryl maltose neopentyl glycol (LMNG) supplemented with brain lipids at pH 7.5 (Methods, Supplementary Fig. 1a). The purified NHE6 protein was reconstituted into MSP1D1 nanodiscs together with yeast polar lipids. We collected 10,514 movies, and the final 3D-reconstruction contained data of 127,459 particles, from which an EM map was reconstructed to 3.3 Å according to the gold-standard Fourier shell correlation (FSC) 0.143 criterion (Supplementary Fig. 1b, c and Supplementary Table 1).

**Fig. 1:**
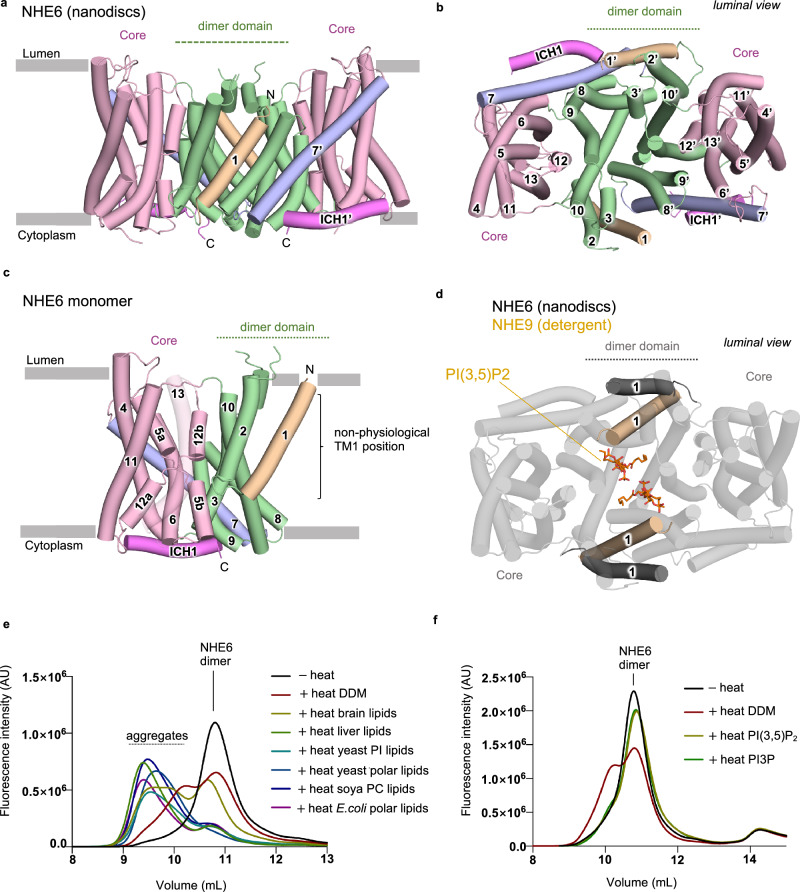
The overall architecture of rat NHE6 in nanodiscs and analysing lipid preferences of the homodimer. **a** Cartoon representation of NHE6 structure in nanodisc with homodimer shown from the membrane plane with core domain (light pink), dimer domain (pale green), linker helix (light purple), TM1 (sand) and intracellular helix ICH1 (pink). **b** Extracellular view of a cartoon representation of NHE6 in nanodiscs, consisting of 13 TM helices labelled and colored as in (**a**). **c** Cartoon representation of the NHE6 monomer, which is made of 6-TM core domain and dimer domain colored as in (**a**). The core domain has two broken helices TM5a-b and TM12a-b shown in light pink, which cross-over in the centre of the membrane forming a putative ion binding site. For illustrative purposes TM13 has been rendered transparent **d**. Overlay of NHE6 nanodisc structure (grey) with NHE9*CC (PDB ID: 8PVR) (colour) with PI(3,5)P_2_ lipids as sticks (orange). The figure highlights the difference in the position of TM1 at dimer interface.** e** FSEC traces of NHE6-GFP at 4 $^\circ {{\rm{C}}}$ and heated at 45 $^\circ {{\rm{C}}}$ following addition of different DDM-solubilised lipids. **f** FSEC traces of NHE6-GFP at 4 $^\circ {{\rm{C}}}$ and heated at 45 $^\circ {{\rm{C}}}$ following addition of PI3P and PI(3,5)P_2_ lipids.

The NHE6 monomer is made up of 13 TMs with an extracellular N-terminus and intracellular C-terminus. We could also model an intracellular helix (ICH1) of the C-terminal domain (CTD), sitting parallel to the membrane interface (Fig. 1a–c). Although the N-terminus of NHE6 is often modelled facing the cytoplasm^34^, this discrepancy has likely arisen because the first predicted TM segment is, in fact, a cleavable signal peptide, as moderately predicted (*p* = 0.7) by signalP^35^ (Methods, Supplementary Figs. 2 and 3a). The mass of detergent purified NHE6, as determined by native mass spectrometry, was consistent with cleavage at the predicted signal sequence site at residues 43 to 44 (Supplementary Fig. 3b). The rat NHE6 structure is thus similar to other NHEs from the SLC9A family^19–21^ and the modelled length of TM1 is equivalent to that observed for the closest isoform NHE9^32^. However, based on the location of the predicted signal peptide cleavage site, we were unable to model the first ∼26 amino acids of NHE6, which AlphaFold2^36^ also predicts as alpha-helical (Supplementary Fig. 3a). NHE9 lacks a cleavable signal sequence and has a shorter N-terminal tail (Supplementary Fig. 2). The functional reason for the TM1 extension in NHE6 is unclear, but its additional length could make TM1 more dynamic. Consistent with this reasoning, the cryo EM structure of the NHE6 homodimer shows a dimerization interface different to *horse* NHE9^19^, wherein TM1 had been repositioned away from the dimer interface (Fig. 1c, d). The unusual repositioning of TM 1 means that the buried surface area for the interface is only 1100 Å^2^ as compared to 1500 Å^2^ in NHE9 (Methods). In NHE9, PI(3,5)P_2_ lipids were located at the dimer interface with polar interactions formed between TM1 and the phosphate lipid headgroups (Fig. 1d)^32^. Although NHE6 was reconstituted into nanodiscs with yeast lipids that retained NHE9 functional activity^32^, we observed for NHE6 no additional density for lipids at the dimer interface. The cryo EM structure of rat NHE6 in nanodiscs may therefore represent a non-physiological dimer arrangement.

We have previously shown that ligand-like lipid interactions stabilizing the homodimer in Na^+^/H^+^ exchanges can be detected by a GFP-based thermostability assay^19,22,29,32^. To investigate the lipid preferences of rat NHE6, we destabilised the detergent purified protein for 10 min at 45 °C in the presence of either DDM or various DDM-solubilised lipids (Methods). Somewhat unexpectedly, almost all lipids added were more destabilizing than just DDM addition, including the yeast lipids used for nanodisc reconstitution (Fig. 1e). The only exception was brain lipids, which were able to retain the homodimer to the same extent as DDM. The thermostability analysis indicated that externally added lipids could be outcompeting bound lipids that had been retained during purification. The closest isoform to NHE6 is NHE9, which binds the endosomal specific PI(3,5)P_2_ lipid at its dimeric interface^32^. NHE6 and NHE9 are both endosomal exchangers, but NHE6 is thought to be localized primarily to early endosomes, rather than the late endosomal location for NHE9^6^. Early endosomes contain a higher fraction of PI3P lipids, but also small amounts of PI(3,5)P₂ lipids^37^. Lipidomic analysis of detergent purified NHE6 indeed showed that lipids matching the mass and fragmentation pattern of PI3P and PIP_2_ (18:0, 20:4) were retained during purification (Supplementary Fig. 3c). Anionic lipids PI and PS were also prevalent, whereas the more abundant PC showed relative diminishment (Supplementary Fig. 3d). Repeating the thermostability analysis confirmed that the addition of either synthetic PI3P or PI(3,5)P₂ lipids did stabilise the NHE6 homodimer (Methods, Fig. 1f).

**Fig. 2:**
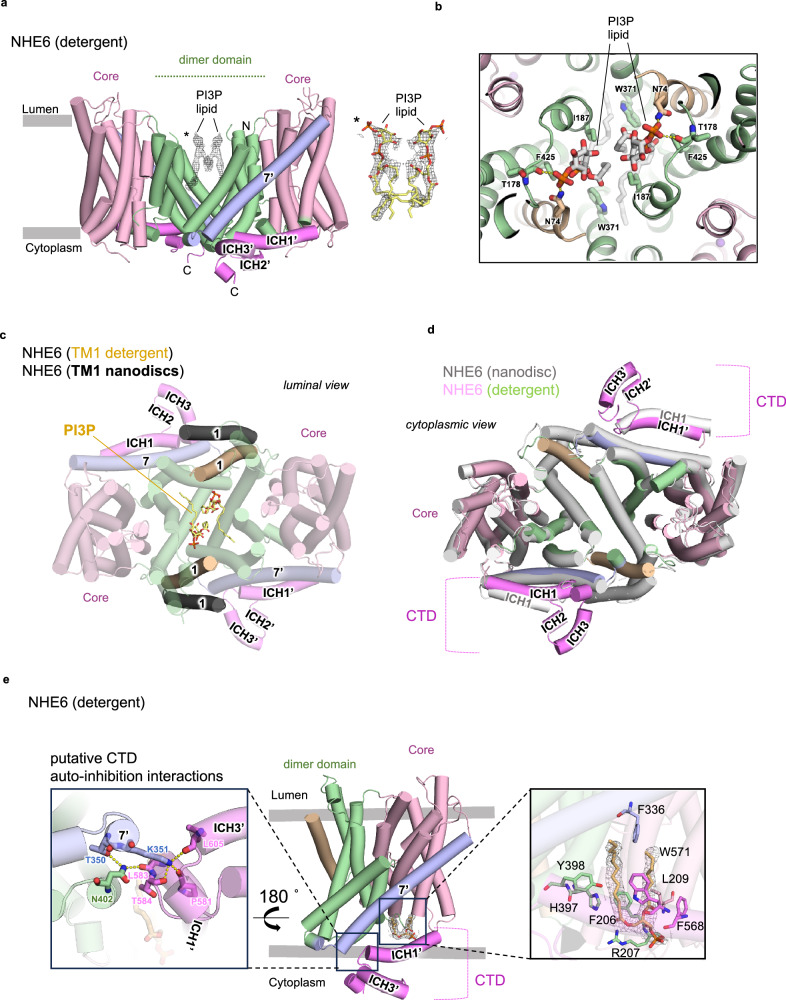
NHE6 structure in detergent with PI3P lipids bound at the homodimer interface and C-terminal domain (CTD) interactions. **a** *left:* Cartoon representation of NHE6 detergent structure where the lipid densities are highlighted at the dimer interface. *right:* Density fit for PI3P lipid shown in yellow sticks. **b** PI3P coordination at the dimer interface by the aromatic and polar residues Trp371, Phe425, Asn74 and Ile187 (sticks, with interactions illustrated by dotted yellow lines). **c** Luminal view of the NHE6 structure in detergent (colored as in Fig. 1a, with TM1 in light-brown), and overlaid is the position of TM1 in the nanodisc structure (black). PI3P lipids are shown in sticks (yellow). In the presence of PI3P the TM1 has repositioned towards the center of the dimer interface.** d** Cytoplasmic view of the structural superimposition of the NHE6 structure in nanodiscs (grey) with NHE6 in detergent (coloured as in Fig. 1a). In the detergent structure more of the CTD was observed, and the ICH2 and ICH3 helices could also be modelled. **e** Cartoon representation of the NHE6 monomer from the detergent structure, highlighting the interactions between the CTD helices ICH1-3 and the transporter module. *left:* magnification of the strictly-conserved positively-charged residue in TM7, K351 in rat NHE6, that forms interactions to back-bone carbonyl oxygen atoms located in the end of ICH1 and the beginning of ICH3. *right:* magnification showing the highly-coordinated PA lipid, observed between ICH1 of the CTD and the transporter module. The cryo EM maps (grey mesh) for the PA lipid (orange sticks) are also shown.

Based on the lipid-stabilization analysis, we repeated the purification of rat NHE6 as previously, wherein brain lipids were added to wash buffers, but rather than reconstitution into nanodiscs with yeast lipids, we blotted the protein directly onto cryo EM grids after the addition of synthetic PI3P lipids (Methods). We collected 14,826 movies, and the final 3D-reconstruction contained data of 256,599 particles, from which an EM map was reconstructed to 2.7 Å according to the gold-standard Fourier shell correlation (FSC) 0.143 criterion (Supplementary Fig. 4a, b and Supplementary Table 1). The cryo EM maps for rat NHE6 in detergent were improved and we observed lipid-like densities at the dimer interface (Supplementary Fig. 4c). Whilst the cryo EM maps indicate two binding poses for the lipid head-group, the stronger map density supports the fitting of two PI3P lipids (Fig. 2a, b and Supplementary Fig. 4b). In the presence of PI3P lipids, TM1 had repositioned to form a more extensive dimer interface (1500 Å^2^), which now closely matches the NHE9 dimer interface with PI(3,5)P_2_^32^ (Fig. 2c, d). The asparagine residue N74 in TM1, had formed hydrogen bonds to C3-phosphate group (Fig. 2b). The glycerol backbone and acyl chains of the PI3P lipid had formed hydrophobic stacking interactions to W371 (TM8, TM8’) (Fig. 2b). The equivalent tryptophan residue to W371 (W321) and a similarly placed glutamine residue in TM1 was previously found to coordinate the PI(3,5)P_2_ lipid in NHE9^32^.

In NHE9, a β-hairpin loop domain located between TM2-TM3 helices clamps above the dimer interface, and conserved lysine residues were further required to bind the PI(3,5)P_2_ lipid^32^. We were, however, unable to refine the β-hairpin loop domain in the rat NHE6 structure, although we could detect its presence in the cryo EM maps at a lower map threshold prior to masked refinement (Supplementary Fig. 4d). Further 3D variability analysis using cryoSPARC^38^ demonstrated that the β-hairpin loop domain adopts multiple positions and was therefore still too dynamic in our preparation to enable its modelling (Supplementary Movie 1). In NHE9, two lysine residues, K107 and K109, in the β-hairpin loop domain were shown to be essential for PI(3,5)P_2_ binding^19,32^. One of these lysine residues K157 (K107 in NHE9) is conserved in the β-hairpin of NHE6 (Supplementary Fig. 4e). It’s plausible that the β-hairpin loop domain stability is still required for full coordination and stabilization of PI3P lipids binding to NHE6, which may explain the relatively weaker lipid density observed at the dimer interface as compared to the PI(3,5)P_2_ lipids previously modelled in the NHE9 structure^32^.

### Regulation of the C-terminal domain (CTD) mobility in NHE6

In humans, although all thirteen Na^+^/H^+^ exchangers catalyze essentially the same reaction, it is thought many isoforms are required, as each isoform has evolved to respond to different external stimuli^1^. The long C-terminal domain (CTD) in NHEs shows the largest sequence variation between isoforms (Supplementary Fig. 2), as it regulates ion-exchange activity in response to these different external cues^1,39,40^. Nevertheless, cryo EM structures of NHE1^20^, NHE3^21^ and NHE9^19,32^ have shown that they have a conserved intracellular helix (ICH1), and more recent structures of NHE9 indicate the CTD likely inhibits Na^+^/H^+^ exchange by restricting movements of the linker helix TM7^32^. In NHE6, we find that K351 in TM7 has formed extensive interactions to the backbone of residues at helical break points of ICH1 and ICH3 from the CTD (Fig. 2e). This same interaction network was observed between the equivalent TM7 lysine (K307) and ICH1 in NHE9^32^. The TM7 residue is a strictly conserved arginine or lysine residue that in AlphaFold3^41^ predictions, forms this interaction across the entire NHE family (Supplementary Figs. 2a and 5). Because this interaction represents the only main contact formed between the NHE transporter module and the CTD, it is reasonable to propose that this interaction constitutes a conserved autoinhibitory interaction. Nevertheless, even with this interaction, the CTD is still likely flexible as we were not able to model a twenty-one-residue long loop between the end of TM13 and the start of ICH1 (Supplementary Fig. 2b). Moreover, in the nanodisc structure only ICH1 could be resolved, and this helix was positioned further from the transporter module as compared to ICH1 in the detergent structure (Fig. 2c, d). The CTD positioning may also be sensitive to lipid binding, as we further observed lipid density between the beginning of ICH1 and the transporter module in the detergent structure (Fig. 2e and Supplementary Fig. 4b). In particular, a phosphatidic acid (PA) lipid fitted well into the cryo-EM maps, and the fatty acid tails were interacting with W571 in ICH1. Although the PA lipid may represent a regulatory lipid site for the CTD, the lack of specific interactions with the lipid headgroup indicates other anionic lipids like PI or PS could also bind (Fig. 2e).

**Fig. 3:**
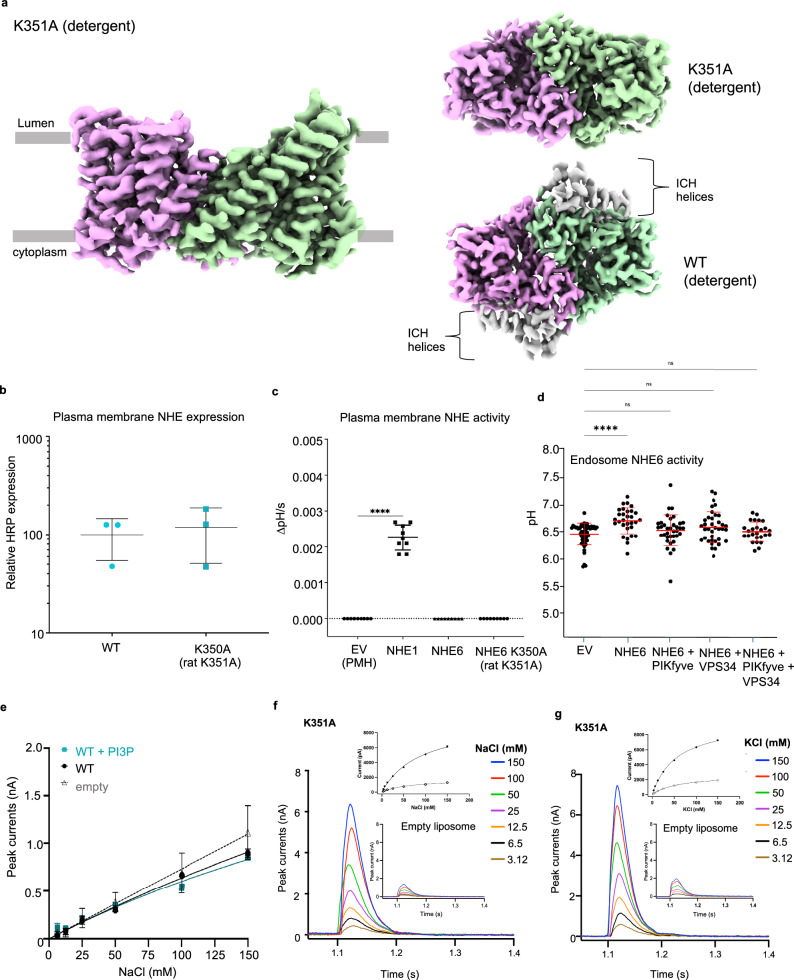
Structure of rat NHE6 variant K351A variant lacking CTD map features and in vivo and in vitro NHE6 activity. **a** *left:* Side-view of the cryo EM maps of the K351A variant with protomers in pink and green respectively. *right above*: As in the left panel, but cytoplasmic view. *right below*: Cryo EM maps of NHE6 WT with the additional map features of the CTD domains colored grey, with protomers shown as in (**a**). **b** NHE6 (*n* = 3) and NHE6 K350A (*n* = 3; rat NHE6 K351A) expression in plasma membrane based on HRP signals. Data are presented as mean values ± SD. **c** Plasma membrane NHE transport activity in PS120 cells transfected with: empty vector (EV; negative control; *n* = 3, with three independent experiments), NHE1 (positive control, *n* = 3) NHE6 (*n* = 3), and NHE6 K350A (rat NHE6 K351A; *n* = 3) with three independent experiments, quantified by the sodium-dependent intracellular pH recovery after acidification of the cytoplasm. Asterisk denotes significance for the indicated comparison (two-tailed unpaired Student’s *t*-test; ****p  <  0.0001). Data are presented as mean values ± SD. **d** Recycling endosome pH measurement of PS120 cells transfected with empty vector (EV; negative control; *n* = 55) or human NHE6, either untreated (*n* = 30) or treated with inhibitors PIKfyve (*n* = 36), VPS34 (*n* = 37), and both (*n* = 26). Data shown are individual observations with mean ± SD of three individual experiments combined. Each dot represents value from an individual cell. Prior to pH measurements, cells were treated with PIKfyve and/or VPS34 inhibitor at final concentrations of 0.25 μM for 4 h. Asterisk denotes significance for the indicated comparison (two-tailed unpaired Student’s *t*-test; ****p < 0.0001); see Supplementary Fig. 7c for pH calibration curve for endosomal pH measurements. **e** Fit of the amplitude of the normalized transient currents as a function of the Na^+^ concentration at pH 7.5 for NHE6 WT (black trace) and after addition of PI3P (blue trace) using proteoliposomes of crude brain lipids. Empty liposomes were used as control (grey trace). Error bars are the mean values ± SEM of: *n* = 3 individual repeats. **f** Representative Na^+^-induced transient currents for the NHE6 K351A variant in brain lipid liposomes with increasing Na^+^ concentrations. *insert top*: Fit of the peak intensities from the K315A mutant in response to an increasing Na^+^ concentration. Error bars are the mean values ± SEM of: *n* = 3 individual repeats. *Inset bottom:* comparative Na^+^-induced currents recorded for empty (no protein) liposomes. **g** As in (**f**), but with increasing K^+^ concentrations. Source data are provided as a Source data file.

To provide further experimental support for the importance of the observed CTD and transporter module interactions, we substituted the TM7 lysine residue with alanine and repeated cryo-EM structural analysis of the K351A variant in the presence of PI3P. With the K351A mutant, we could obtain a final cryo-EM map with an improved resolution of 2.38 Å (Supplementary Fig. 6a–c and Supplementary Table 1). Despite cryo EM maps reconstructed to a higher resolution than rat NHE6 WT, we were unable to observe any map features for the CTD (Fig. 3a). Apart from the lack of CTD map features, however, the WT and K351A structures were very similar (Fig. 3a), although map density for the PI3P lipid was somewhat weaker than in the WT structure in detergent (Supplementary Fig. 6c). Overall, the K351A structural data supports the proposal that the main interaction between the CTD and the transporter module are formed between the backbone of ICH1 and ICH3 and the side-chain of a strictly-conserved lysine or arginine in the TM7 linker helix (Fig. 2e and Supplementary Fig. 2).

We have previously shown that human NHE9 is inactive when mistargeted to the plasma membrane, likely as it requires the endosomal-specific lipid PI(3,5)P_2_ for activation^32^. To evaluate NHE6, we expressed (endosomal) human NHE6 as well as (plasma membrane-localized) human NHE1 in a PS120 cell line, which is deficient in plasma membrane NHEs (Methods). To rule out auto-inhibition by the CTD at the plasma membrane, we also expressed the human K350A variant, which corresponds to K351A in rat. In this mutant cell line, we could confirm plasma membrane localization of NHE1, NHE6 WT and NHE6 K350A after transient transfection by surface biotinylation, isolation and immunoprecipitation (Supplementary Fig. 7a). To measure NHE activity, the pH-sensitive dye BCECF was trapped intracellularly, and pH recovery after acid loading with NH_4_Cl was assessed spectroscopically (Methods). Whilst Na^+^-dependent intracellular pH recovery was clearly measurable in PS120 cells expressing human NHE1, no NHE activity could be measured for either human NHE6 or the K350A variant, despite substantial plasma membrane expression (Fig. 3b, c and Supplementary Fig. 7a). In comparison, human NHE6, which also localized to Rab5 containing early endosomes in PS120 cells, demonstrated the expected activity within endosomes (Fig. 3d and Supplementary Fig. 7b, c). The addition of kinase inhibitors PIKfyve and VPS34 required for producing PI(3,5)P_2_ and PI3P lipids, respectively, further abolished the observed pH difference in NHE6 expressing endosomes (Fig. 3d and Supplementary Fig. 7d). This cell-based data is consistent with our proposal that NHE6 is inactive when at the cell surface, likely because the plasma membrane lacks the endosomal PI3P lipid.

### Solid Supported Membrane-based (SSM) electrophysiology recordings support PI3P binding and CTD auto-inhibition

We have previously measured Na^+^-induced transient currents for *horse* NHE9 and *bison* NHA2 Na^+^/H^+^ exchangers incorporated into proteoliposomes using solid-supported membrane (SSM)-based electrophysiology^22,32^. In brief, Na^+^-induced transient currents are recorded in electroneutral transporters as, in the absence of a pH gradient, the rate of Na^+^ influx is considerably faster than H^+^ efflux^22^. The positive amplitude of the pre-steady-state Na^+^-induced transient currents is clearly distinguishable from the negative amplitude electrogenic currents seen in the bacterial Na^+^/H^+^ exchangers with a 1:2 ion stoichiometry^42^. Because the rat NHE6 structure in detergent has a clear interaction between ICH1-3and the transporter module and is, therefore, likely in an auto-inhibited state, we first optimised the SSM-based electrophysiology setup using the K351A variant. Because rat NHE6 has likely retained PI3P lipids when purified in detergent buffer supplemented with brain lipids, we compared the Na^+^ induced currents in liposomes made from either these brain lipids or yeast lipids, which gave rise to the non-physiological dimer structure in nanodiscs. As an additional comparison, we also reconstituted NHE6 into *E. coli* lipids, which is unlikely to retain ion-exchange activity (Methods) (Supplementary Fig. 7e). Satisfactorily, we see a clear difference in the Na^+^-induced currents between the different NHE6 preparations. The peak area of the Na^+^-induced currents was substantially higher for NHE6 protein incorporated into brain lipid liposomes than yeast lipid liposomes and there were no clear peak currents in *E. coli* lipid liposomes (Supplementary Fig. 7e). Further, a Na^+^-concentration dependent titration shows only weak NHE6 activity in liposomes using yeast lipids as compared to the brain lipid liposomes, for which we could measure an apparent *K*_d_ Na^+ app^ = 109 mM (Supplementary Fig. 7f). In comparison, NHE6 WT shows no apparent activity above background levels with or without PI3P addition in liposomes made from brain lipids (Fig. 3e). However, with optimization the K351A variant in liposomes made from brain lipids demonstrated strong Na^+^- and K^+^-induced transient currents as compared to empty liposomes (Fig. 3f, g). Thus, we can use SSM to follow NHE6 activity of the K351A variant and the analysis supports the proposal that the WT protein was likely purified in an auto-inhibited state.

**Fig. 4:**
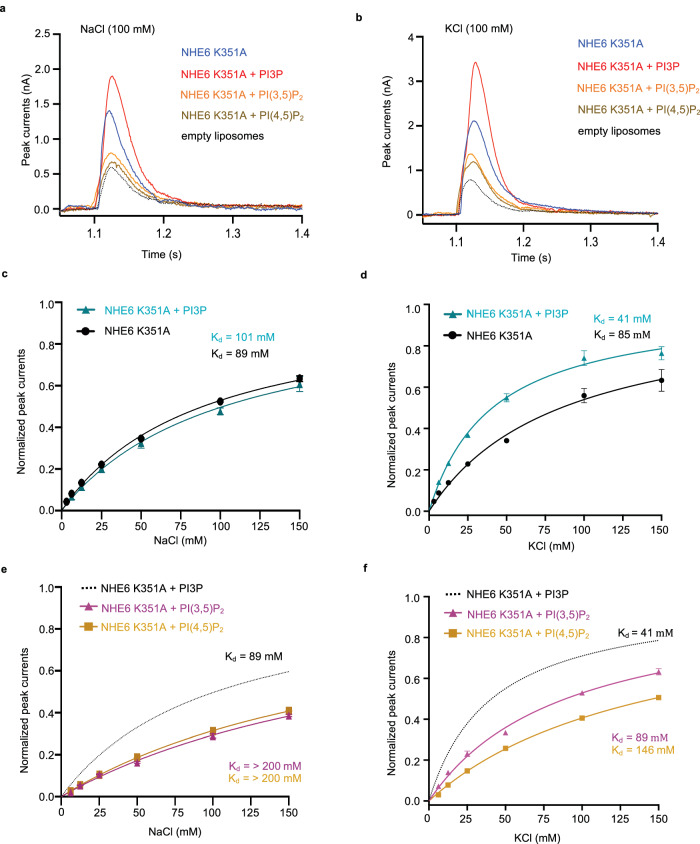
NHE6 lipid preference and ion selectivity. **a** Representative Na^+^-induced transient currents for K351A reconstituted into liposomes made from brain VII extract with or without prior addition of either synthetic PI3P, PI(3,5)P_2_ or PI(4,5)P_2_ lipids. **b** As in **a**., for K^+^ **c**. Fit of the peak intensities of the normalized transient currents as a function of Na^+^ concentrations for K351A proteoliposomes with or without PI3P addition, with the corresponding binding affinities (*K*_d_). Error bars are the mean ± SEM for *n* = 3 technical repeats; the average *K*_d_ values are from *n* = 3 independent titrations and summarized in Supplementary Table 2. **d** As in **c**., for K^+^. **e** Fit of the peak amplitude of the normalized transient currents as a function of Na^+^ concentrations for K351A with either PI(3,5)P_2_ or PI(4,5)P_2_ lipids added prior to reconstitution, with the corresponding binding affinities (*K*_d_) shown together with the data for the NHE6 K351A + PI3P sample from c shown for comparison. The dotted line represents the fit of normalized currents for K351A with PI3P as shown in **c**, together with the data for the NHE6 K351A + PI3P from d for comparison. Error bars are the mean ± SEM for *n* = 3 technical repeats; the average Kd from *n* = 3 independent titrations is summarized in Supplementary Table 2. **f**. As in e., for K^+^. Source data are provided as a Source data file.

We next assessed the impact of different lipids by adding either synthetic PI3P, PI(3,5)P_2_ or PI(4,5)P_2_ lipids prior to reconstitution of purified NHE6 K351A variant into brain lipid liposomes for SSM-based electrophysiology measurements (Methods). Interestingly, we observed higher Na^+^ and K^+^ transient peak currents with PI3P addition than without, whereas PI(3,5)P_2_ and PI(4,5)P_2_ lipids decreased peak currents (Fig. 4a,b). The average *K*_d_^app^ with Na^+^ with and without PI3P addition were similar at 95 mM vs 102 mM (Fig. 4c, and Supplementary Table 2), whereas the K^+^ K_d_^app^ was somewhat lower at 77 mM vs 55 mM (PI3P) (Fig. 4d and Supplementary Table 2). In contrast, consistent with the weak transient currents, both PI(3,5)P_2_ and PI(4,5)P_2_ lipids were inhibitory, with *K*_d_ values > 200 mM for Na^+^ and between ∼100 and 140 mM for K^+^ (Fig. 4a, b, e, f and Supplementary Table 2). Given the ∼10-fold higher concentration of K^+^ vs. Na^+^ in the cytoplasm, we conclude that under physiological conditions NHE6 likely operates as a K^+^/H^+^ exchanger. Furthermore, SSM-based activity measurements support the proposal that PI3P lipids found in early endosomes enhance NHE6 stability and activity.

### Further details of ion binding site in NHE6 structure

**Fig. 5:**
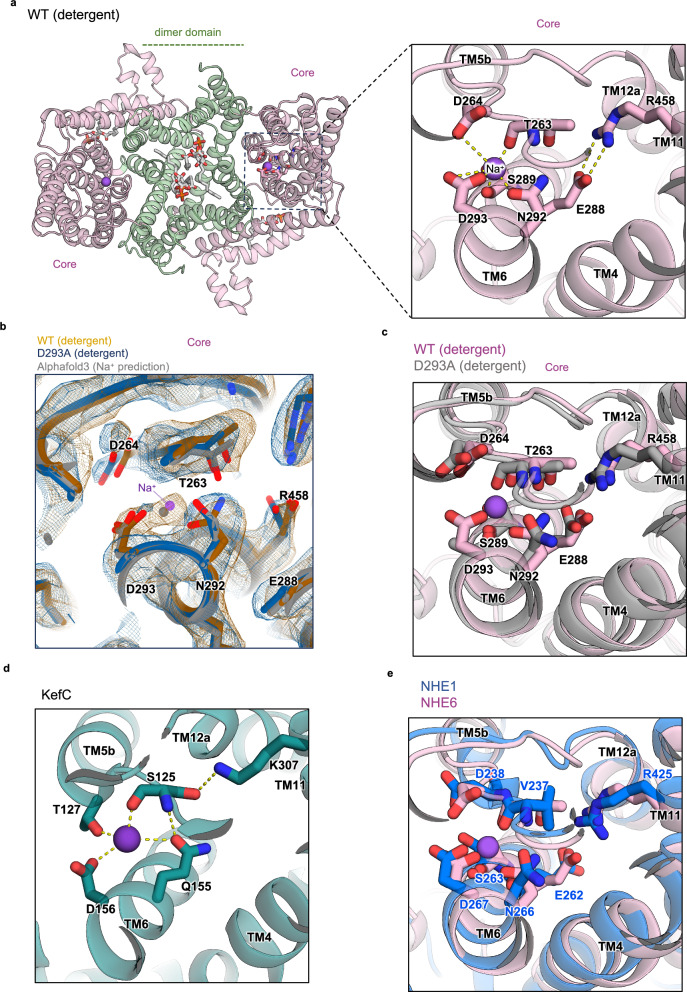
Characterization of the ion binding site of NHE6. **a** *left:* cartoon representation of the NHE6 WT homodimer as viewed from luminal side with the respective domains colored individually and labelled. The conserved ion-binding site is highlighted by the dashed square. *right:* magnified representation of the NHE6 core transport domain harboring the ion binding residues. Na^+^ ion is tightly coordinated by the highly conserved ND motif residues, N292 and D293, in TM6, and interactions from the surrounding main-chain carbonyls from T263 and S289. The conserved salt-bridge interaction between E288 and R458 is also shown. **b** Superimposition of the models and their respective cryo-EM map densities of the NHE6 WT (orange) and D293A variant (blue) structures together with Alphafold3^42^ Na^+^ model, with maps rendered at the same threshold. Even with a higher overall resolution of 2.4 Å no clear map density was observed for a bound ion (purple) following mutation of the key ion binding D293 residue to alanine. **c** Superposition of NHE6 WT (pink) and D293A (gray) variant structures showing moderate side-chain differences at the ion binding site. **d** K^+^ coordination in the bacterial K^+^/H^+^ exchanger KefC, with key residues shown as sticks. Direct interaction of the conserved K307 from TM11 with the ion binding residue S125 of the breakpoint helix TM5 makes the binding site more rigid. In NHE6, the equivalent R458 residue instead interacts with E288 in TM6. **e** Comparison of the ion binding site of NHE1 (blue) and NHE6 (pink). Except T263, all the other ion binding site residues are strictly conserved (see Supplementary Fig. 2).

Similar to NHE9, the rat NHE6 structure has been captured with a cavity open to the cytoplasm (Fig. 5a). In the high-resolution K351A and WT structures in detergent we could observe additional map density that would be consistent with the location of either a bound Na^+^ or K^+^ ion (Fig. 5b). To provide further experimental support, we substituted into alanine the key ion-binding aspartate D293, which is known to be essential to Na^+^/H^+^ exchanger activity^1,43^. After collecting 17,850 movies we obtained a final 3D-reconstruction of the D293A variant to 2.28 Å resolution (Supplementary Fig. 8a–c and Supplementary Table 1). The structure of the D293A variant was likewise in the cytoplasmic-facing conformation, and the density of the PI3P lipid was similar to the WT protein in detergent (Supplementary Fig. 8a–c). When maps are contoured to the same level, however, we see no clear map density for the ion (Fig. 5b). Furthermore, in the absence of ion binding in the D293A variant, the N292 side-chain is orientated away from the ion (Fig. 5c). So far, this WT structure is the only example of an Na^+^/H^+^ exchanger where we have been able to experimentally observe the position of a bound (substrate) ion.

Given the NHE6 protein was blotted in the presence of 150 mM NaCl the map density is more likely to represent Na^+^ rather than K^+^. Using the check-my-metal server^44^, Na^+^ is the preferred bound ion with the observed side side-chain coordination. The Na^+^ ion is coordinated within the core domain and by the conserved ion-binding aspartate D293 and N292 residues, which are positioned next to the half-helical cross-over (Fig. 5a). The ion is further coordinated by backbone interactions of S289 as well as backbone and side-chain interactions to T263 (Fig. 5a). Interestingly, of the half helices the Na^+^ ion is only directly coordinated by the TM5a-b and there are no direct interactions from TM12a-12b. Yet proximal to the ion-binding site is the conserved salt-bridge between E288 in TM6 and R458 in TM11^45^, which has been observed in previous NHE1^20^, NHE3^21^ and NHE9^19^ structures—an interaction that likely stabilizes the core domain (Fig. 5a). Although the role of this conserved salt-bridge has been unclear, from the current Na^+^-bound state it suggests that its presence enables the stabilization of the ion-binding site as well as connecting to the TM12a-b helices, since R458 also interacts with the backbone of M485 in TM12b (Fig. 5a).

In MD simulations of NHE9 the Na^+^ was found to concentrate at two locations that we interpreted as an initial (peripheral) interaction location principally with D293 and a translocation Na^+^ site, located deeper in the core domain^19^. The ion binding site location of Na^+^ is consistent with the later (translocated) Na^+^ ion position^19^ as predicted by AlphaFold3^41^ (Fig. 5c), and as was likewise modelled with a bound K^+^ ion in the K^+^-specific K^+^/H^+^ exchanger KefC (Fig. 5d). KefC has short half-helical breaks as well as lysine residue in TM11 that directly interacts with residues in the ion-binding site (Fig. 5d). Alchemical perturbation calculations of KefC show a preference for K^+^ ion binding because of its lower penalty for dehydration compared with Na^+^^26^, and K^+^ was modelled to be coordinated in a partially-hydrated state (Fig. 5d). We postulate that the more rigid ion-binding site structure in KefC may exclude Na^+^ binding, whereas NHE6 has a more flexible ion-binding site, with a salt-bridge residue formed between E288 in TM6 and R458 in TM11 instead. This difference may allow either K^+^ or a partially-hydrated Na^+^ to bind to NHE6. Consistent with this interpretation, in the Na^+^-specific homologue NHE1 the residue equivalent to T263 in NHE6 is a valine (Fig. 5e). We postulate this difference means that the ion binding site in NHE1 is less polar and more flexible than in NHE6 and will require additional waters for the ion coordination, and thus disfavoring K^+^ from binding.

### PI(4,5)P_2_ lipid stabilizes auto-inhibited state of NHE6

**Fig. 6:**
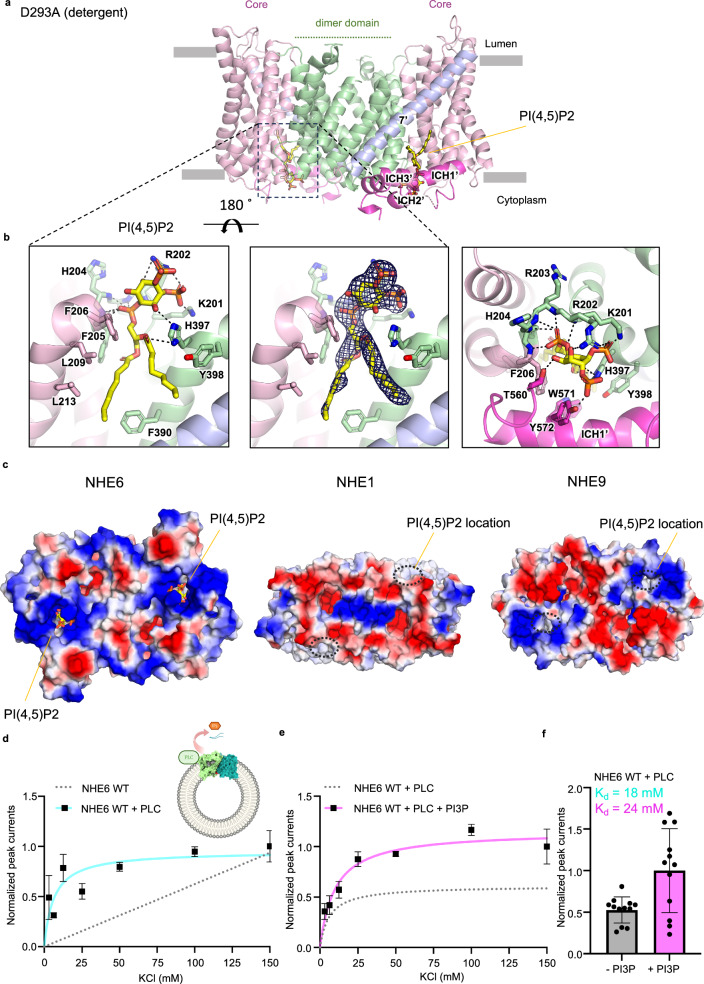
Architecture of PI(4,5)P_2_ binding site. **a** A PI(4,5)P_2_ lipid was observed at the interface of the core, dimerization and ICH domains near the cytosolic interface of the D293A variant, suggesting its potential regulatory role. Cartoon representation colored as in Fig. 1a with lipids shown as sticks. **b** Several residues from core (pink), dimerization (green) and ICH (violet) domains provide key interactions and appeared to be held together by the PI(4,5)P_2_ lipid (yeallow) as seen in the two views from the cytoplasmic side with the density of the lipid shown as blue mesh. **c** Electrostatic surface potential comparison between NHE6 (PI(4,5)P_2_ complex), NHE1 (PDB:7DSV) and NHE9 (PDB: 8PXB) colored blue (positive) to red (negative). PI(4,5)P_2_ resides in the highly positively charged cavity in NHE6, with the corresponding position indicated for NHE1 and NHE9. **d** Fit of the peak intensity of the normalized transient currents as a function of K^+^ concentrations for NHE6 WT proteoliposomes following PLC incubation; Sample without PLC shown previously in Fig. 3e is plotted as dotted-line. *Insert*: illustration of experimental setup with PLC acting on PI(4,5)P_2_. Fit of the normalized amplitude of the transient currents is shown. Error bars are the mean ± SEM for *n* = 3 technical repeats; the average *K*_d_ from *n* = 4 independent titrations is summarized in Supplementary Table 2. **e** As in **d**, but PI3P lipids were added after PLC addition and without shown in **d** (dotted-line). **f** K^+^-induced transient currents at 100 mM KCl with and without PI3P addition to NHE6 WT proteoliposomes incubated with PLC. Error bars are the mean ± SEM for *n* = 12 independent measurements from 4 sensors; the average *K*_d_ from *n* = 4 independent titrations is shown and summarized in Supplementary Table 2. Source data are provided as a Source data file.

Whilst refining the D293A variant structure we observed additional map density that was an unambiguous match for a PI(4,5)P_2_ lipid, which was located between the CTD and transporter module (Fig. 6a). Compared to the PA lipid modelled in the WT structure, the PI(4,5)P_2_ lipid is better coordinated by the positively-charged K201 and R202 residues that interact with the negatively charged phosphate lipid head-groups (Fig. 6a, b). Further, the position of F206 and R202 have adjusted their position to better coordinate the lipid (Fig. 6b). Unexpectedly, PI(4,5)P_2_ lipid interacts with residues not only located in the dimer domain, but also the core domain and the CTD, which collectively would almost certainly stabilize NHE6 in the auto-inhibited state. Comparing the electrostatic surface potential of the NHE6, NHE9 and NHE1 structures we observe that the highly-positively-charged surface is specific to the endosomal NHEs (Fig. 6e). This positively charged surface is ideally suited for negatively-charged lipids like PIP_2_, and likely explains their retention during purification, as was previously assessed by lipidomic analysis (Supplementary Fig. 3c). Consistently, in the GFP-based thermostability assay we found that PI(4,5)P_2_ lipids also stabilize NHE6 WT (Supplementary Fig. 9). We thus propose that because the D293A variant is less dynamic than the WT protein, it was possible to capture NHE6 predominantly in an auto-inhibited state.

In an effort to distinguish between the PI3P and PI(4,5)P_2_ lipid binding sites, we repeated the GFP-thermostability assay by adding either PI3P or POPC lipids together with the PI(4,5)P_2_ lipids. Whilst PI3P afforded no additional thermostability than PI(4,5)P_2_ on its own, the addition of POPC was destabilizing (Supplementary Fig. 9). PI3P is unlikely to bind tightly to the PI(4,5)P_2_ site given that this lipid was not observed in the CTD of any structures, including the D293A variant, despite the fact that synthetic PI3P lipids were added prior to blotting, i.e, under blotted conditions PI3P concentrations would be several magnitudes higher than PI(4,5)P_2_. Furthermore, only PI(4,5)P_2_ addition to purified rat NHE6 decreased Na^+^(K^+^)-induced peak currents (Fig. 4c, d). Since PI3P does not clearly bind to the CTD, it reasonable to assume that POPC would even bind more poorly, and so we interpret the reduced stability as competition of POPC for the PI3P lipid binding site.

To provide support for the interpretation of PI(4,5)P_2_ inhibition, we wondered if the binding of PI(4,5)P_2_ lipids was the reason we were unable to record SSM-based electrophysiology currents for the rat NHE6 WT in brain-lipid-liposomes, which also contain PI(4,5)P_2_^46^. We further reasoned that if this was the case, the inhibition could be removed by incubation of proteoliposomes with the enzyme phospholipase C, i.e., to cleave PI(4,5)P_2_ lipids into IP3 and DAG (see Methods). Satisfactorily, after PLC addition, we could detect K^+^-induced peak currents for the WT NHE6 protein (Fig. 6d). Furthermore, PI3P addition to PLC treated NHE6 liposomes further increased activity, on average, by ∼ 200% (Fig. 6d, e). Although the apparent average *K*_d_^app^ for K^+^ with and without PI3P addition is similar at 18 and 21 mM, the interaction is stronger than what was measured for the K351A variant (41 mM), which implies that PI(4,5)P_2_ was still partially inhibiting in the CTD mutant (Fig. 6e and Supplementary Table 2). This conclusion would be consistent with the reduced activity measured upon PI(4,5)P_2_ addition to detergent-solubilised K351A (Fig. 4e. f and Supplementary Table 2). Overall, we can confirm that the CTD auto-inhibits NHE6 and this interaction is stabilized by PI(4,5)P_2_ lipids.

### Christianson syndrome mutations mapped onto NHE6

Mutations in human NHE6 are the known cause of the X-linked neurological disorder Christianson syndrome^8,14,15^. We therefore mapped the location of sense mutations onto the rat NHE6 structure (Supplementary Fig. 10a, b). Interestingly, despite the fact that the core domain binds and transports the substrate ion across the membrane, only two of the disorder-causing mutations are located within the core domain (Supplementary Fig. 10b). The core domain mutations G218R and G449R are fairly drastic, as glycine allows for TM bending, which would clearly be perturbed if mutated to arginine. Rather, apart from R568Q mutation in ICH1 that could affect PI(4,5)P_2_ binding, all of the other disorder-causing sense mutations are located in the dimer (scaffold) domain. One of these mutations, W90R in TM1 would likely perturb the position and perhaps mobility of TM1, as tryptophan is important for membrane bilayer anchoring. Other disorder mutations, L189P and P190L, are clustered in TM3, which has a conserved hydrophobic surface important for core transport domain^19^. Moreover, TM2-TM3 contains the β-hairpin domain, and TM3 mobility is likely affected by the binding of PI3P lipids. Indeed, a disorder deletion V177 to R202 is situated next to the PI3P binding site (Supplementary Fig. 10a, b). Taken together, although it is unclear if disorder-causing sense mutations would be less tolerated in the core domain, it nonetheless reinforces that the scaffold domain is far from a rigid element, but rather its flexibility and response to specific lipid is likely to be a fundamental property of NHE6 function.

## Discussion

Our structure and mechanistic analysis of the endosomal Na^+^/H^+^ exchanger NHE6 highlights the complexity in the allosteric regulation of ion-exchangers regulating intracellular pH. Perhaps this is unsurprising, given that intracellular and organellar pH needs to be exquisitely regulated and that the Na^+^/H^+^ exchanger family plays an important role in fine-tuning intracellular pH^1,47^. The inability to regulate pH properly is pronounced in the Christianson disorder due to mutations in human NHE6^8,14,15^. Essentially, NHE6 dysfunction leads to excessive acidification of recycling endosomes and, in neurons, the disrupted trafficking compromises endosomal–lysosomal function in Purkinje, cortical, and hippocampal tissues^9,15,47,48^.

Here we demonstrate that a compacted and likely functional NHE6 homodimer is facilitated by the presence of the endosomal PI3P lipid. Empirically, we found that addition of brain lipids was required to reduce aggregation of the rat NHE6 protein during purification from HEK293 cells. Moreover, if rat NHE6 protein was reconstituted into the non-physiological yeast lipid environment in nanodiscs, we found that the homodimer interface was weakened by the displacement of TM1. Further cryo EM structures showed that when synthetic PI3P lipid was added, the homodimer interface in NHE6 did adjust and interact with TM1 to increase the buried surface area of the homodimer interface. The binding of PI3P was further supported by lipidomic analysis, SSM-based electrophysiology, and thermal-shift assays. We further demonstrated that, similar to human NHE9^32^, the human NHE6 protein was nonfunctional at the plasma membrane—a membrane lacking the endosomal-specific PI3P and PI(3,5)P_2_ phosphoinositide lipids. Taken together, we propose that endosomal NHE6 and NHE9 proteins have evolved a dimer interface that requires trafficking to the correct organelle before endosomal-specific lipids can trigger a more compacted homodimer for full activation. The fact that the additional β-hairpin domain is not found in NHEs at the plasma membrane^32^, supports the proposal this domain has a regulatory role. Clearly, further structural and functional analysis will be required to determine exactly how the β-domain engages with PI3P in NHE6 and how this domain can discriminate between the binding of PI3P *vs* PI(3,5)P_2_ in the closely-related isoform NHE9.

An unexpected finding we observed in the D293A variant was the additional map density for PI(4,5)P_2_ lipids located between the NHE6 transporter module and ICH1-3 in the CTD. Given the extensive PI(4,5)P_2_ lipid coordination and the relative low abundance of PI(4,5)P_2_ lipids, we find it highly unlikely that this lipid co-purified with rat NHE6 non-specifically. Even taking this viewpoint, the structure demonstrates that PI(4,5)P_2_ can clearly bind tightly to NHE6, as it was retained during purification in detergent. Furthermore, the PI(4,5)P_2_ lipid is coordinated by strictly conserved residues only found in endosomal NHEs (Supplementary Fig. 2 and Fig. 6c). PI(4,5)P_2_ binding to this location would undoubtedly stabilise the auto-inhibited state of NHE6. Indeed, we could only observe ion-induced currents for either the K351A variant with a detached CTD or for the WT NHE6 protein after incubation with PLC—used to enzymatic dephosphorylate PI(4,5)P_2_ lipids in brain-lipid-liposomes.

**Fig. 7:**
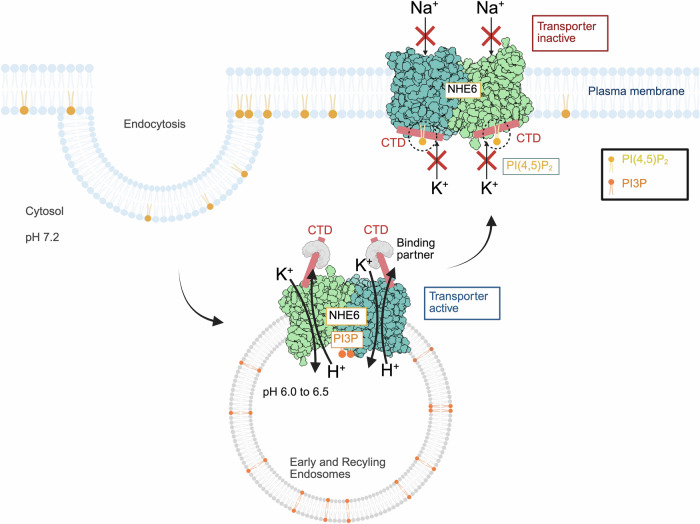
Schematic of lipid-dependent regulation of endosomal NHE6. There is no measureable activity of endosomsal human NHE6 at the cell surface. In vitro analysis demonstrated that rat NHE6 binds PI(4,5)P_2_ lipids to stabilize the auto-inhibited state and inactivate ion-exchange. During NHE6 endocytosis and its recycling into early endosmes the phosphoinisitol composition in the membrane is altered, which likely weakens binding of the inhibitory PI(4,5)P_2_ lipid. NHE6 may also become fully activated in early endosomes by PI3P binding at the homodimerization interface as well as the binding of other accessory proteins (grey) to the CTD. Once activated, NHE6 preference is to exchange cytoplasmic K^+^ with luminal H^+^ to fine-tune endosomal pH.

We propose that upon trafficking to the plasma membrane, the binding of PI(4,5)P_2_ auto-inhibits NHE6 (Fig. 7). Without PI3P lipids, it is possible that NHE6 at the plasma membrane could adopt the more open dimer structure seen for NHE6 in the non-physiological yeast lipids, which would further help to keep NHE6 inactivated. Indeed, although PI(4,5)P_2_ addition clearly auto-inhibited the rat K351A variant in vitro, given no activity was detected for the corresponding human K350A variant in cell-based measurements, implies that a CTD displacement is unlikely to be sufficient on its own for full activation. Rather, we propose that upon trafficking to endosomes (i) the binding of PI3P lipids and (ii) the binding of other accessory proteins to the CTD^32^, are likely required to facilitate CTD detachment. It is further plausible that the relatively higher fraction of PS lipids found in endosomes will out-compete the endocytosis retained NHE6-bound PI(4,5)P_2_ lipids, resulting in a destabilisation of the auto-inhibited state. Exactly the preference for different lipids as well as interacting proteins with the CTD and their role, together with phosphorylation, in fine-tuning NHE6 activity via modulation of the described auto-inhibition interaction is still yet to be determined.

Once activated, ion exchange in NHE6 will be driven solely by the relative ion concentration differences pre-established across the endosomal membrane^49^. Kinetic modelling of bacterial Na^+^/H^+^ exchangers has demonstrated that activity follows a simple ping-pong model, whereby Na^+^(K^+^) ions compete for the same binding site as H^+^^42^. The V-type ATPase acidifies endosomes and synaptic vesicles, and the H^+^ ions are then exchanged for cytoplasmic K^+^ or Na^+^ ions in a pump-leak mechanism to fine-tune the appropriate vesicular pH^5^. The cryo EM structure of WT NHE6 in detergent confirms the location of the substrate ion entirely within the core domain, as expected for an elevator alternating-access mechanism^18,49,50^. Binding affinity estimates by SSM-based electrophysiology analysis support that K^+^ rather than Na^+^ is likely be the main physiological counter-ion transported under most physiological conditions. Consistent with this study, a recent pre-print on the cryo EM structure of human NHE6 has likewise demonstrated a preference for K^+^ *vs* Na^+^ ion and that the CTD bound a stabilized lipid at a similar location^51^.

Taken together, we propose the extensive regulation of lipid-mediated interactions observed here is to ensure that NHE6 activity is completely shut-down upon endosomal fusion with the plasma membrane, that is, to avoid inadvertent Na^+^/K^+^ exchange activity that would effectively run-down the membrane potential at the plasma membrane (Fig. 7). Intriguingly, during metazoan evolution, the relocation of NHEs from endosomes to the plasma membrane coincided with the loss of K^+^ binding and its specificity for Na^+^^43^. Lastly, the Christianson disorder-causing mutations in human NHE6^8,14,15^ cluster around the dimer domain and in helices sensitive to the coordination of PI3P lipid. Given this large pocket is accessible to lipids in detergent, it would be interesting to explore if small molecules could interact to stabilise TM3 and correct mutations in this rare genetic disorder.

In summary, our findings highlight how specific phosphatidylinositol lipids can switch transporters on- and-off, as well as the molecular basis of NHE6 ion-exchange activity and regulation. These observations have broader implications and highlight that cell-surface expression of other membrane proteins, e.g., GPCRs, may not necessarily be functional in tissues lacking specific, regulatory lipids.

## Methods

### Expression and purification of rat NHE6 and nanodisc reconstitution

The gene encoding the NHE6 protein from *Rattus Norveticus* (XP_217630.3), referred to as *SLC9A6*, was synthesized and subcloned (Thermo Fisher Scientific) into the pcDNA3.1(+) vector, followed by a TEV (Tobacco Etch Virus) cleavage site, eGFP, and a TwinStrep tag at the C-terminus. The K351A variant of NHE6 was constructed using the QuickChange kit (Life Technologies). The nucleotide sequence of the generated vectors was verified by DNA sequencing and purified using the PureLink Giga Plasmid Purification Kit (Invitrogen). Serum-free suspension-adapted Freestyle HEK293F cells (Invitrogen) were used for recombinant protein expression and cultured in FreeStyle 293 expression medium (Life Technologies), shaken at 140 rpm in a humidified incubator at 37 °C and under an 8% CO_2_ atmosphere. Cell growth and viability were assessed using a Countess II FL automated cell counter (Invitrogen). The cells were transiently transfected using 20 kDa linear polyethylenimine (PEI) (PolySciences). Transfections were performed using a final DNA concentration of 1 μg ml^−1^. In brief, the cloned Slc9a6-gfp containing pcDNA3.1(+) vector variants was diluted in FreeStyle medium and vortexed for 10 s. PEI-20KDa was diluted using the same medium at a 1:3 (w/w) DNA:PEI ratio and vortexed for 10 s before being added to the DNA-containing medium. The DNA:PEI mixture was incubated for 10 min at room temperature and added to the cells at a cell density of 1–1.5 × 10^6^ cells per mL. Then, 24 h after transfection, sodium butyrate was added to a final concentration of 5 mM. Typically, after 72 h, 10 µL of cell culture was collected by centrifugation (3000 × *g* at 4 °C for 10 min). The cells were subsequently resuspended in 100 ml cell resuspension buffer (20 mM Tris-HCl pH 7.5, 150 mM NaCl) supplemented with 1 tablet of cOmplete, EDTA-free protease inhibitor cocktail (Roche) and subsequently lysed using a homogenizer. The resulting membranes containing the overexpressed protein were then isolated by ultracentrifugation at 195,000 × *g* and 4 °C for 1 h.

For structural studies of NHE6 in nanodiscs, isolated NHE6-GFP containing membranes were solubilized in buffer containing 1% (w/v) DDM (Glycon), 50 mM Tris-HCl pH 7.5, 150 mM NaCl, and 10% (v/v) glycerol for 2 h at 4 °C with mild agitation. The detergent-solublised membranes were subsequently cleared by ultracentrifugation 195,000 × *g* and 4 °C for 1 h. The supernatant was incubated for 2 h at 4 °C with 5 ml Strep-Tactin XT resin (IBA Life Sciences) pre-equilibrated with wash buffer consisting of 0.1% (w/v) DDM (Glycon), 50 mM Tris-HCl pH 7.5, 150 mM NaCl. This resin was transferred into a gravity flow column (BioRad) and subsequently washed in two steps, initially with wash buffer consisting of 0.1% (w/v) DDM (Glycon), 50 mM Tris-HCl pH 7.5, 150 mM NaCl and finally with wash buffer consisting of 0.03% (w/v) DDM, 50 mM Tris–HCl pH 7.5, 150 mM NaCl and eluted in 1 x BXT buffer consisting of 50 mM Tris-HCl pH 7.5, 150 mM NaCl with addition of 0.03% (w/v) DDM. NHE6-GFP protein was concentrated and subsequently mixed with yeast polar lipids (Sigma-Aldrich) and MSP1D1 nanodiscs at a molar ratio of 1:5:50 for 30 min at room temperature. After incubation, 50 mg of preactivated SM-2 Bio-Beads (Bio-Rad) was added, followed by an overnight incubation at 4 °C. The Bio-Beads were discarded following centrifugation at 16,900 × g for 5 min. The NHE6-containing-nanodiscs were re-bound to Strep-Tactin XT resin (IBA Life Sciences) pre-equilibrated with wash buffer consisting of 50 mM Tris-HCl pH 7.5, 150 mM NaCl. The resin was washed with 50 mM Tris-HCl pH 7.5, 150 mM NaCl, and the resin-bound GFP-TwinStrep-His_8_ was digested by TEV-His_8_ protease added in excess amounts during mild agitation overnight at 4 °C. Following centrifugation the cleaved NHE6-containing-nanodiscs were collected from the resulting supernatant and concentrated using 100 kDa MW cut-off spin concentrators (Amicon, Merck-Millipore). The reconstituted nanodiscs were subjected to size-exclusion chromatography on a Superose 6 increase column in a buffer containing 50 mM Tris pH 7.5 and 150 mM NaCl. The NHE6-containing nanodisc peak fractions were collected and concentrated to 1.5 mg/ml using 100 kDa MW cut-off spin concentrators (Amicon, Merck-Millipore).

For structural studies of NHE6 in detergent, isolated NHE6-GFP membranes were solubilized in solubilization buffer containing 1% (w/v) LMNG (Anatrace), 50 mM Tris-HCl pH 7.5, 150 mM NaCl, 0.003% (w/v) Brain extract from bovine brain type VII (brain fraction VII, Sigma-Aldrich), and 10% glycerol for 2 h at 4 °C with mild agitation. Non-solubilized material was removed by ultracentrifugation 195,000 × *g* at 4 °C for 1 h. The NHE6-GFP-containing supernatant was incubated for 2 h at 4 °C with 5 ml Strep-Tactin XT resin (IBA Life Sciences) pre-equilibrated with wash buffer consisting of 0.1% (w/v) LMNG (Anatrace), 50 mM Tris-HCl pH 7.5, 150 mM NaCl. This resin was transferred into a gravity flow column (Bio-Rad) and subsequently washed in two steps, initially with wash buffer consisting of 0.1% (w/v) LMNG (Anatrace), 50 mM Tris-HCl pH 7.5, 150 mM NaCl, and finally with wash buffer consisting of 0.003% (w/v) LMNG, 50 mM Tris–HCl pH 7.5, 150 mM NaCl. The resin-bound NHE6-GFP-TwinStrep-His_8_ was digested by TEV-His_8_ protease added in excess amounts during mild agitation overnight at 4 °C. The cleaved protein was collected and concentrated using 100 kDa MW cut-off spin concentrators (Amicon, Merck-Millipore). The concentrated NHE6 protein was subsequently injected onto a pre-equilibrated Superose 6 Increase 10/300 column (GE Healthcare) in LMNG buffer consisting of 50 mM Tris-HCl pH 7.5, 150 mM NaCl, and 0.03% (w/v) LMNG, and the peak fraction was collected. Peak fractions were concentrated to 2 mg/ml using a 100 kDa MW cut-off spin concentrator (Amicon, Merck-Millipore). The NHE6 protein was further incubated with final (v:v) of 1 mg/ml PI3P (Phosphatidylinositol 3-phosphate dipalmitoyl; Echelon Biosciences, Cat. No. P-3016) dissolved in 50 mM Tris-HCl pH 7.5, 150 mM NaCl.

Purification of NHE6 variants was carried out as described for WT, except supernatant was applied to a GFP nanobody-coupled CNBr-activated Sepharose 4B resin (Cytiva) and incubated for 1 h at 4 °C, and subsequently washed with 10 column volumes of wash buffer consisting of 0.03% LMNG (w/v), 20 mM Tris-HCl pH 7.5, 150 mM NaCl, and 0.003% (w/v) brain fraction 7. The resin-bound GFP-TwinStrep-His_8_ was digested by TEV-His_8_ protease added in excess amounts during mild agitation overnight at 4 °C. The cleaved NHE6 protein was collected and concentrated using 100 kDa MW cut-off spin concentrators (Amicon, Merck-Millipore). The concentrated NHE6 protein was subsequently injected onto a pre-equilibrated Superose 6 Increase 10/300 column (GE Healthcare) in LMNG SEC buffer consisting of 50 mM Tris-HCl pH 7.5, 150 mM NaCl, and 0.03% (w/v) LMNG, and the peak fraction was pooled and concentrated to 2 mg/ml using a 100 kDa MW cut-off spin concentrator (Amicon, Merck-Millipore). Purified NHE6 variants were further incubated with final (v:v) 1 mg/ml of PI3P (Phosphatidylinositol 3-phosphate dipalmitoyl; Echelon Biosciences, Cat. No. P-3016) dissolved in 50 mM Tris-HCl pH 7.5, 150 mM NaCl.

### Cryo-EM sample preparation and data acquisition

A total of 9 µg of NHE6 WT in nanodiscs and 4.5 µg NHE6 WT in detergent was applied to freshly glow-discharged Quantifoil R2/1 Cu300 mesh grids (Electron Microscopy Sciences), blotted for 3 s, under 10% humidity, and plunge-frozen in liquid ethane using a Vitrobot Mark IV (Thermo Fisher Scientific). Cryo-EM data were acquired using a Titan Krios G3i transmission electron microscope operated at 300 kV, equipped with a Gatan Imaging Filter (GIF) and a K3 BioQuantum direct electron detector (Gatan) in counting mode. Movie stacks were recorded at a magnification of 130,000×, yielding a pixel size of 0.65 Å in super-resolution counting mode, with a defocus range of −0.4 to −2.0 µm. Similarly, 2.5 µg of K351A was applied on Quantifoil R2/2 Cu300 mesh grids and 4.0 µg of D293A on Quantifoil R1.2/1.3 Cu300 mesh grids under the same blotting conditions. Movies were collected at 130,000× magnification with a pixel size of 0.65 Å using a Titan Krios G3i transmission electron microscope operating at 300 kV, equipped with a GIF (Gatan) and a K3 BioQuantum direct electron detector (Gatan) in counting mode. The defocus range for these datasets was set between −0.4 and −2.0 µm.

### Cryo-EM image processing

Collected datasets were processed using CryoSparc^38^. Movies were corrected by Patch Motion Correction and Patch CTF Estimation, and further particles were picked using reference based auto-picking for all the datasets. For NHE6 structure in nanodiscs, 3,288,197 particles were subjected to several rounds of 2D classification. 842,384 particles were used for ab initio model building and hetero refinements. The final 3D reconstruction resulted in map resolution of 3.25 Å. NHE6 detergent and its K351A and D293A variant structures were refined using a similar strategy, and the final map’s gold standard FSC resolutions were estimated to be around 2.7 Å, 2.38 Å and 2.28 Å, respectively. The data processing parameters are summarized in Supplementary Table 1.

### Cryo EM model building and refinement

AlphaFold2 model^36^ of rat NHE6 was fitted into the cryo-EM density map of nanodisc and NHE6 WT, respectively. For Model building and real-space refinement COOT^52^ and PHENIX.refine^53^ were used. For NHE6 K351A and NHE6 D293A, the NHE6 WT model was fitted into the respective cryo-EM density maps, and subsequently model building and real-space refinement were performed as mentioned above. The refinement statistics are summarized in Supplementary Table 1. Structural figures were made using PyMOL^54^ and ChimeraX^55^.

### FSEC-based thermal shift assay

To assess lipid binding to NHE6, we employed the FSEC-Thermal Shift assay^56^. Purified NHE6-GFP fusion protein was prepared similarly to the nano-disk sample, but after elution from the strep-resin the undigested protein was injected onto a Enrich 650 column (Bio-Rad) pre-equilibrated in 20 mM Tris-HCl pH 7.5, 150 mM NaCl, 0.03% (w/v) DDM (Glycon) and the resulting NHE6-GFP peak was collected and concentrated using a 100 kDa MW cut-off concentrator at 4 °C (Amicon, Sigma-Aldrich). The protein was diluted to a final concentration of 0.05 – 0.075 mg/ml in a buffer containing 20 mM Tris-HCl, pH 7.5, 150 mM NaCl, 0.03% (w/v) DDM (Glycon). DDM was added to a final concentration of 1% (w/v), and samples were incubated at 4 °C for 30 min. Lipid stocks for liver total lipid extract (bovine; Avanti, cat. no. 181104), yeast polar lipid extract (*S. cerevisiae*; Avanti, cat. no. 190001), brain extract from bovine brain type VII (Sigma-Aldrich, cat. no. B3635), L-α-phosphatidylinositol from yeast (PI; Larodon, cat. no. 37-0132), *E. coli* polar extract (100600 C, Avanti), Soy PC (Avanti, cat. no. 441601 G) were prepared by solubilization with in 10% (wt/vol) DDM to a final concentration of 30 mg ml^−1^ were prepared by mild agitation, overnight at 4 °C. For testing effect of PIP derivatives on NHE6-GFP, PI3P diC16 (P-3016, Echelon Biosciences), PI(3,5)P₂ diC16 (P-3516, Echelon Biosciences), and PI(4,5)P₂ diC16 (P-4516, Echelon Biosciences) were solubilized in 20 mM Tris-HCl pH 7.5, 150 mM NaCl to 1 mg/mL. 10% DDM was added into the lipids and solubilized for 1 h.

The respective lipids were added to individual samples of NHE6-GFP to a final concentration of 0.1 mg/ml for crude lipids and 0.3 mg/mL for PIP derivatives, with 10 min incubation on ice. β-D-Octyl glucoside (Anatrace) was then added to a final concentration of 1% (w/v) in a 300 µl total volume. Aliquots of 100 µl were subjected to heat treatment at 45 °C for 10 min using a Veriti PCR thermocycler (Applied Biosystems). Heat-denatured proteins were pelleted by centrifugation at 5000 × *g* for 30 min at 4 °C. From each sample, 80 µl of the supernatant was injected onto an Enrich SEC 650 10 × 300 column (Bio-Rad), pre-equilibrated with buffer containing 20 mM Tris-HCl pH 7.5, 150 mM NaCl, and 0.03% (w/v) DDM. Fluorescence-detection size-exclusion chromatography (FSEC) was performed (488_excitation_/512 _emission_ nm) using a Shimadzu LC-20AD/RF-20A HPLC, and FSEC traces were analysed using GraphPad Prism (version 9.5).

### Native mass spectrometry of NHE6 WT

Native mass spectra of rat NHE6 WT were recorded on a Q-Exactive UHMR mass spectrometer (Thermo Fisher Scientific). The protein was buffer-exchanged into 200 mM ammonium acetate containing 2 × CMC C_8_E_4_ detergent using 40 K MWCO Zeba Micro Spin desalting columns (Thermo Fisher Scientific). The proteomicelle solution was ionized in positive mode by nano-electrospray ionization (1.1 kV) using gold-coated borosilicate emitters pulled and coated in house. The following instrument parameters were used: capillary temperature: 200 °C; desolvation voltage: −250 V; collisional activation in the HCD cell: 100 V; trapping gas pressure: 10; Orbitrap resolution: 12,500. The spectra were deconvoluted using UniDec^57^.

The identity of HSP70 was confirmed by native top-down mass spectrometry using the Orbitrap Eclipse Tribrid mass spectrometer (Thermo Fisher Scientific) in high-pressure, intact protein mode. The 18^+^ charge state of HSP70 monomer was isolated in the linear ion trap (*m*/*z* 3900 ± 10 Th) and fragmented by HCD (CE 130). Protein fragments were measured in the Orbitrap (resolution: 240,000 @ *m*/*z* 200). b- and y-ions were assigned using precisION^58^.

### Lipidomics analysis of purified NHE6

Lipidomics analysis of phospholipids copurified with rat NHE6 WT was performed as described previously^59^. Briefly, 25 µg of NHE6 protein purified in LMNG was diluted to a final volume of 14 µL and buffer-exchanged into 200 mM ammonium acetate (pH 7.0) containing 2× CMC GDN detergent using 40 K MWCO Zeba Micro Spin desalting columns (Thermo Fisher Scientific). The samples were incubated with trypsin at a 1:10 trypsin:NHE6 ratio at 37 °C overnight with shaking at 750 rpm. The samples were briefly centrifuged, and 1 µL SPLASH II lipidomix mass spec standard (Avanti Research) was added to the samples destined for standard lipidomics. All samples were dried in a SpeedVac vacuum concentrator. For standard lipidomics analysis, the dried peptides and lipids were resuspended in 30 µl lipidomics buffer (80% buffer A/20% buffer B), sonicated for 5 min and centrifuged at maximum speed for 10 min to remove potential GDN precipitate. For analysis of phosphoinositides, the dried samples were redissolved in 15 µL methanol and diluted with 15 µL H_2_O, followed by sonication and centrifugation. The solutions were transferred to glass vials for lipidomics.

For standard lipidomics, the following buffers were used: buffer A containing acetonitrile/H_2_O (60/40, v/v), 10 mM ammonium formate, 0.1 % (v/v) formic acid, and buffer B containing isopropanol/acetonitrile (90/10, v/v), 10 mM ammonium formate, 0.1 % (v/v) formic acid. Liquid chromatography-tandem mass spectrometry (LC-MS/MS) was performed using a Dionex UltiMate 3000 system coupled to an Orbitrap Eclipse Tribrid mass spectrometer (Thermo Fisher Scientific). The chromatography system was equipped with a C18 column (Acclaim PepMap 100, C18, 75 µm × 15 cm, Thermo Fisher Scientific). During the 37 min chromatographic runs, the concentration of buffer B was increased from 20 % to 99 % using the same gradients profile as in ref.^59^. Survey MS scans were acquired in the Orbitrap (*m*/*z* 300–2000) with a resolution of 120,000 @ *m*/*z* 200. MS^2^ spectra were acquired with stepped HCD (25 and 30 % NCE in positive mode and 25 and 35 % NCE in negative mode) with an Orbitrap resolution of 15,000 @ *m*/*z* 200. Lipids were assigned using LipiDex^60^.

For the targeted analysis of phosphoinositides, the following buffers were used: buffer A containing MeOH/H_2_O (50/50, v/v), 0.12% (v/v) ethylamine, and buffer B containing isopropanol, 0.12% (v/v) ethylamine. The instrument setup and column were the same as described above. During the 37 min chromatographic run, buffer B was increased from 5 % to 95 % using the same gradient profile as in ref. (DOI 10.1038/s41596-024-01037-4). Survey MS scans were acquired in the Orbitrap (*m*/*z* 300–2000) with a resolution of 120,000 @ *m*/*z* 200 in negative ion mode. The MS^2^ spectra were acquired using stepped HCD (30 and 35% NCE) with a resolution of 15,000 @ *m*/*z* 200. The setup was tested with a mixture of synthetic PI, PI3P and PI(4,5)P_2_ (Avanti Research). Triplicate raw LC-MS/MS files were converted using MSConvert^61^ for lipid identification by LipiDex^60^ with custom-generated libraries for PIP and PIP2.

### Cell-based human NHE1, NHE6 and NHE6 K351A

#### Cell culture, constructs and transfection

All cells used in the experiments were confirmed to be mycoplasma-free and maintained at 37 °C in a humidified incubator with a 95% air/5% CO₂ atmosphere. PS120 fibroblasts, NHE1-null cells derived from Chinese hamster lung fibroblast CCL39 cells^62^ were cultured in high-glucose DMEM supplemented with 10% fetal bovine serum (FBS), 100 U/mL penicillin, and 100 µg/mL streptomycin (all from Sigma-Aldrich). Human full-length NHE6 was cloned into the pMH vector (Roche) with a C-terminal HA tag, while human full-length NHE1 was cloned into the p3xFLAG-CMV-14 vector (Sigma-Aldrich) with a C-terminal 3×FLAG tag. The NHE6 K350A mutant was generated from the pMH-NHE6 plasmid by replacing lysine (K) at position 350 with alanine (A) using QuikChange™ site-directed mutagenesis (Agilent). All constructs were verified by sequencing. Transient transfection was performed using Lipofectamine 2000 (Invitrogen) according to the manufacturer’s protocol. Experiments, including cell surface expression and transport function studies, were conducted 48 h post-transfection.

#### Cell surface biotinylation

Cell surface biotinylation was conducted as previously described^63^. Cells were rinsed with 1× PBS, then incubated with 1.5 mg/mL sulfo-NHS-LC-biotin in a labeling buffer containing 10 mM triethanolamine (pH 7.4), 1 mM MgCl₂, 2 mM CaCl₂, and 150 mM NaCl for 90 min at 4 °C with gentle horizontal agitation. After labeling, the plates were washed with a quenching buffer (1× PBS supplemented with 1 mM MgCl₂, 0.1 mM CaCl₂, and 100 mM glycine) for 20 min at 4 °C, followed by an additional rinse with 1× PBS. Cells were then lysed in RIPA buffer (150 mM NaCl, 50 mM Tris-HCl, pH 7.4, 5 mM EDTA, 1% Triton X-100, 0.5% deoxycholate, and 0.1% SDS), and the lysates were cleared by centrifugation. Equal amounts of protein from each lysate were incubated overnight at 4°C with streptavidin-agarose beads. The beads were sequentially washed with three different buffers: solution A (50 mM Tris-HCl, pH 7.4, 100 mM NaCl, 5 mM EDTA) three times, solution B (50 mM Tris-HCl, pH 7.4, 500 mM NaCl) twice, and solution C (50 mM Tris-HCl, pH 7.4) once. Finally, biotinylated proteins were eluted by heating to 95 °C in 2.5× Lämmli buffer.

#### SDS-PAGE, immunoblotting and antibodies

SDS-PAGE and immunoblotting were performed as previously described^64^. The proteins of the respective cell lysates were separated by SDS-PAGE and transferred onto PVDF membranes. The membranes were blocked for 60 min in PBS-T20 containing 5% (w/v) skimmed milk, followed by overnight incubation at 4 °C with the indicated primary antibodies in the same blocking solution. After three to six washes with PBS-T20, membranes were incubated for 1 h at room temperature with HRP-conjugated secondary antibodies in 5% (w/v) skimmed milk in PBS-T20. Following additional washes, signal detection was performed using an enhanced chemiluminescence reagent. Immunoblots were developed using a Fujifilm automatic processor, scanned at 600 dpi, and quantified using ImageJ software. The following primary antibodies from Sigma-Aldrich were used at a 1:1000 dilution: anti-HA (#H9658) and anti-Tubulin (#T9028). HRP-coupled goat anti-rat IgG antibody (#31430, Invitrogen) was used at a 1:2000 dilution, while avidin-HRP (#1706528, Bio-Rad) was used at a 1:1000 dilution.

#### NHE1, NHE6 and NHE6-K351A transport activity

NHE activity was measured fluorometrically using the intracellular pH-sensitive dye BCECF-AM (2ʹ,7ʹ-bis-(Carboxyethyl)-5(6ʹ)-carboxyfluorescein Acetoxymethyl Ester; #B1170, Invitrogen) and the NH₄Cl prepulse technique, as previously described^63^. Cells grown on glass coverslips were loaded with 1 µM BCECF-AM and incubated for 15 min in a buffer containing (in mM): 120 NaCl, 5 KCl, 2 CaCl₂, 1.5 MgCl₂, 25 NH₄Cl, and 30 HEPES, titrated to pH 7.4 with N-methyl-D-glucamine (NMDG). Cells were then washed three times and incubated in a buffer containing (in mM): 120 Tetramethylammonium-Cl (TMACl), 5 KCl, 2 CaCl₂, 1.5 MgCl₂, and 30 HEPES, titrated to pH 7.4 with NMDG. Fluorescence recording was initiated, and after 60 s, cells were rapidly exposed to an Na⁺-containing buffer (in mM: 120 NaCl, 5 KCl, 2 CaCl₂, 1.5 MgCl₂, and 30 HEPES, pH 7.4, titrated with NMDG). BCECF fluorescence signals (excitation at 490 and 440 nm, emission at 535 nm) were recorded using a computer-controlled spectrofluorometer (Fluoromax-2, Photon Technology International). The 490/440 nm fluorescence ratio was calibrated to intracellular pH (pHᵢ) using the K⁺/nigericin method, and the initial rate of sodium-dependent intracellular pH recovery (Vmax, pH/time) was calculated, as described previously^63^. All incubation, recording, and calibration steps were performed at 37°C.

#### Immunofluorescence

Cells were fixed with 4% paraformaldehyde (#15713, Electron Microscopy Sciences) in 1x PBS (#14190144, Thermo Fisher Scientific) for 10 min at 4 °C, followed by permeabilization with 0.1% Triton X-100 (#T8787-100ML, Merck) in 1x PBS for 3 min at room temperature. Blocking was performed using 5% bovine serum albumin (BSA, #A7906-10G, Merck) in 1x PBS for 1 h. Fixed cell monolayers were incubated overnight at 4 °C with primary antibodies diluted in 5% BSA. Following three washes in 1x PBS, cells were incubated with Alexa Fluor-conjugated secondary antibodies (Alexa Fluor 488 or 647) (#A32723, #A21245, Invitrogen) for 1 h at room temperature in the dark. Cytoliner dye (#30135-T, Biotium) was stained in 10 min at room temperature for cell membrane. Cells were subsequently washed three times in 1x PBS for 10 min each, counterstained with Hoechst 33342 (#H3569, Invitrogen) for DNA staining, and mounted on glass slides (#K540.1, Carl Roth) using ProLong™ Diamond Antifade Mountant (#P36961, Invitrogen). Confocal images were acquired using a Leica SP8 confocal microscope (Leica Microsystems).

#### Cell viability assay

Cell viability following phosphoinositide kinase inhibition was assessed using the alamarBlue™ HS Cell Viability Reagent (A50100, Thermo Fisher Scientific). Cells were seeded at 10,000 cell density per well in 96-well plate to ensure logarithmic growth and allowed to adhere overnight. Cells were then treated with a PIKfyve inhibitor (YM-201636, #HY-13228, MedChemExpress) or a VPS34 inhibitor (Vps34-IN-1, #HY-12795, MedChemExpress) at final concentrations of 0.25 μM, 0.5 μM, or 1 μM. For combination treatments, cells were exposed to both inhibitors simultaneously at matching concentrations (0.25 + 0.25 μM, 0.5 + 0.5 μM, or 1 + 1 μM). Treatments were performed for 4 h to assess acute cytotoxic effects. Following inhibitor exposure, alamarBlue reagent was added directly to the culture medium at 10% (v/v), and cells were incubated for 4 h at 37 °C. Absorbance was measured at 570 nm and 600 nm using a microplate reader, with culture medium containing alamarBlue but no cells serving as the blank control. Cell viability was calculated as the percentage reduction of alamarBlue relative to untreated control cells, which were defined as 100% viability.

#### Measurement of endosomal pH

Endosomal pH was evaluated using a dual-labeling method that involved a pH-stable Alexa Fluor 633-labeled transferrin and a pH-responsive pHrodo™ Red Transferrin Conjugate (both obtained from Invitrogen), following established protocols. Prior to pH measurements, cells were treated with a PIKfyve inhibitor (YM-201636, #HY-13228, MedChemExpress) or a VPS34 inhibitor (Vps34-IN-1, #HY-12795, MedChemExpress) at final concentrations of 0.25 μM, either individually or in combination at matched concentrations. Treatment durations (4 h) and concentrations were selected based on alamarBlue cell viability assays, which confirmed the absence of acute cytotoxic effects under these conditions. Before labeling, cells were incubated in serum-free DMEM at 37 °C for 1 h to induce starvation. Afterward, the cells were treated for 1 h at 37 °C with Hank’s Balanced Salt Solution (HBSS) containing 25 µg/mL each of Alexa Fluor 633-labeled human transferrin and pHrodo™ Red transferrin. The composition of the HBSS was as follows (in mM): 137 NaCl, 5.3 KCl, 1.3 CaCl₂, 0.82 MgSO₄, 0.34 Na₂HPO₄, 0.44 KH₂PO₄, 4.2 NaHCO₃, 5.6 glucose, and 15 HEPES–NaOH, adjusted to a pH of 7.4. Following thorough washing to eliminate excess probes, fluorescence was recorded at 37 °C using a Leica SP8 confocal microscope with a 63× oil objective. A pH calibration curve was generated using an intracellular pH calibration buffer set containing standard solutions at pH values of 7.5, 6.5, 5.5, and 4.5. Fluorescence signals from perinuclear recycling endosomes were analyzed using ImageJ software. For each experiment, the mean endosomal pH was determined by averaging measurements from 30 randomly selected live cells.

### Solid supported membrane-based electrophysiology

For SSM-based electrophysiology, samples were purified using the same protocol as that used for structural analysis in detergent, except that solubilization was performed with 1% (w/v) DDM, and all buffers contained 0.03% (w/v) DDM instead of 0.003% (w/v) LMNG. To determine the ion binding affinity of NHE6, reconstitution was initially screened using lipids from *E. coli* polar extract (100600C, Avanti), yeast polar lipids (190001C, Avanti), and Brain extract from bovine brain type VII (Sigma-Aldrich, cat. no. B3635). Based on the screening results, all subsequent reconstitution experiments were performed using Brain extract from bovine brain type VII. Lipid preparation was carried out as previously described for NHA2^22^. Size-exclusion chromatography (SEC)-purified NHE6 protein was incorporated into destabilized liposomes at a lipid-to-protein ratio (LPR) of 25:1, followed by incubation for 30 min at room temperature. Proteoliposomes were collected by ultracentrifugation at 195,000 × *g* for 40 min and resuspended in a buffer containing 20 mM Tris-HCl, pH 8.0, 20 mM NaCl, 20 mM KCl, and 5 mM MgCl₂. Samples were diluted to a final lipid concentration of 2 mg/mL using the same buffer. For electrophysiological measurements, proteoliposomes were further diluted 1:1 (v/v) with a non-activating buffer consisting of 20 mM Tris-HCl pH 8.0, 200 mM choline chloride, and 5 mM MgCl₂, and then sonicated using a bath sonicator.

For experiments involving phosphoinositides, PI3P (P-3016, Echelon Biosciences), PI(3,5)P₂ (P-3516, Echelon Biosciences), and PI(4,5)P₂ (P-4516, Echelon Biosciences) were added to the protein to a final concentration of 10 μM and incubated for 30 min prior to reconstitution into Brain VII liposomes. A volume of 10 μL of each sample was applied to 3 mm SSM sensors (#161001, Nanion Technologies).

To test the effect of phosphoinositides on NHE6 WT activity, 0.5 U of Phospholipase C (Invitrogen) was added during proteoliposome reconstitution, followed by shaking for 45 min on under 25 °C. Proteoliposomes were collected via ultracentrifugation at 195,000 × *g* for 40 min and resuspended into 5 mg/mL within a buffer containing 20 mM Tris-HCl pH 8.0, 20 mM NaCl, 20 mM KCl, and 5 mM MgCl₂, followed by a further dilution into 2.5 mg/mL within non-activating buffer. Sample was then loaded onto sensors and centrifuged for 1 h at 2500 × *g*. For experiments involving PI3P, 20 μL of above-mentioned 5 mg/mL suspension containing 4 μg of wtNHE6 was mixed with 0.1 mg/mL of PI3P stock to a molar ratio of 1:5. The mixture was then subjected to a round of shaking under 25 °C for 45 min, and the same 1:1 dilution was carried out prior to sample loading.

SSM-based electrophysiological recordings were performed using the SURFE²R N1 system (Nanion Technologies) according to the manufacturer’s protocol (Methods Enzymol. 594, 31–83, 2017). The non-activating buffer consisted of 20 mM Tris-HCl pH 8.0, 5 mM MgCl₂, and 200 mM choline chloride. The activating buffer had the same composition but contained decreasing amounts of choline chloride (200 − x mM) and increasing concentrations of substrate (x mM NaCl or KCl), such that the total concentration of choline chloride and salt was maintained at 200 mM.

Peak currents were recorded in triplicate with at least three independent 3 mm sensors and analyzed using nonlinear regression fitting (Michaelis–Menten) in GraphPad Prism software. Peak amplitudes were normalized to the average of the maximum response obtained across all measurements. The final *K*_d_ values are the mean ± s.d. of at least independent sensors.

### Reporting summary

Further information on research design is available in the Nature Portfolio Reporting Summary linked to this article.

## Supplementary information

Supplementary Information

Description of Additional Supplementary Files

Supplementary Movie 1

Reporting Summary

Transparent Peer Review file

## Source data

Source Data

## Data Availability

Source data are provided with this paper. The coordinates and the maps for cryo-EM structures of rat NHE6 have been deposited in the Electron Microscopy Data Bank (EMD) Protein Data Bank (PDB) with entries: NHE6-nanodisc (EMDB54661 [https://www.ebi.ac.uk/pdbe/entry/emdb/EMD-54661], PDB 9S8E [10.2210/pdb9S8E/pdb]); NHE6 WT (EMDB54660 [https://www.ebi.ac.uk/pdbe/entry/emdb/EMD-54660], PDB 9S8D [10.2210/pdb9S8D/pdb]); NHE6 K351A (EMDB54662 [https://www.ebi.ac.uk/pdbe/entry/emdb/EMD-54662], PDB 9S8G [10.2210/pdb9S8G/pdb; NHE6 D293A]) (EMDB54659 [https://www.ebi.ac.uk/pdbe/entry/emdb/EMD-54659], PDB 9S8C [10.2210/pdb9S8C/pdb)]. The native MS and lipidomics data have been deposited in MassIVE and can be accessed via DOI: 10.25345/C5862BR8P [10.25345/C5862BR8P]. Source data are provided with this paper.
