# Supplementary material for: Structure of NHE6 and its lipid-mediated interactions regulating endosomal pH
Source: Nat Commun. 2026 Aug 11;17:8177. doi: 10.1038/s41467-026-75877-x (PMC13463026; doi:10.1038/s41467-026-75877-x)
Supplement: Supplementary file 1 [file 41467_2026_75877_MOESM1_ESM.pdf]

This file contains Supplementary Figures 1-10 and Supplementary Tables 1-2

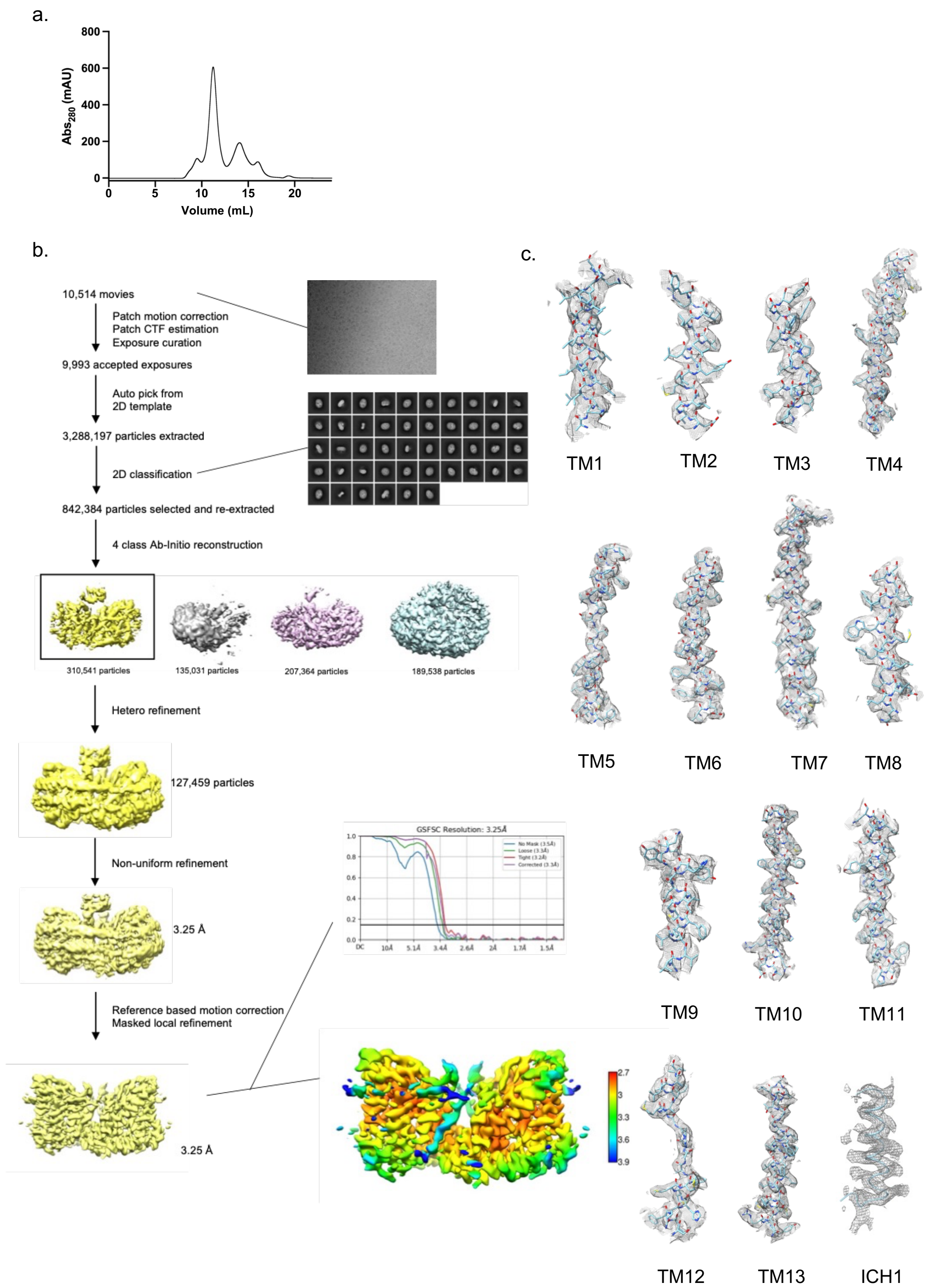

Supplementary Figure 1. The data-processing workflow of NHE6 in nanodiscs a. SEC trace of the LMNG purified rat NHE6 reconstituted into nanodiscs (Methods). **b**. The dataset contained 10,514 movies that were corrected by Patch Motion Correction and Patch CTF Estimation. After reference-based auto-picking, 3,288,197 particles were picked. Several rounds of 2D classifications yielded in good 2D classes of 842,384

particles. 3D classification and 3D refinement of model resulted in an electron density map of 3.25 Å. The electron density map was subjected to non-uniform refinement local refinement and reference based motion correction. A final resolution of 3.25Å was achieved at gold-standard FSC (0.143), with a local resolution range of 2.7–3.9 Å. **c**. Cryo-EM density map and model are shown for all the transmembrane segments for the NHE6 structure in nanodisc.

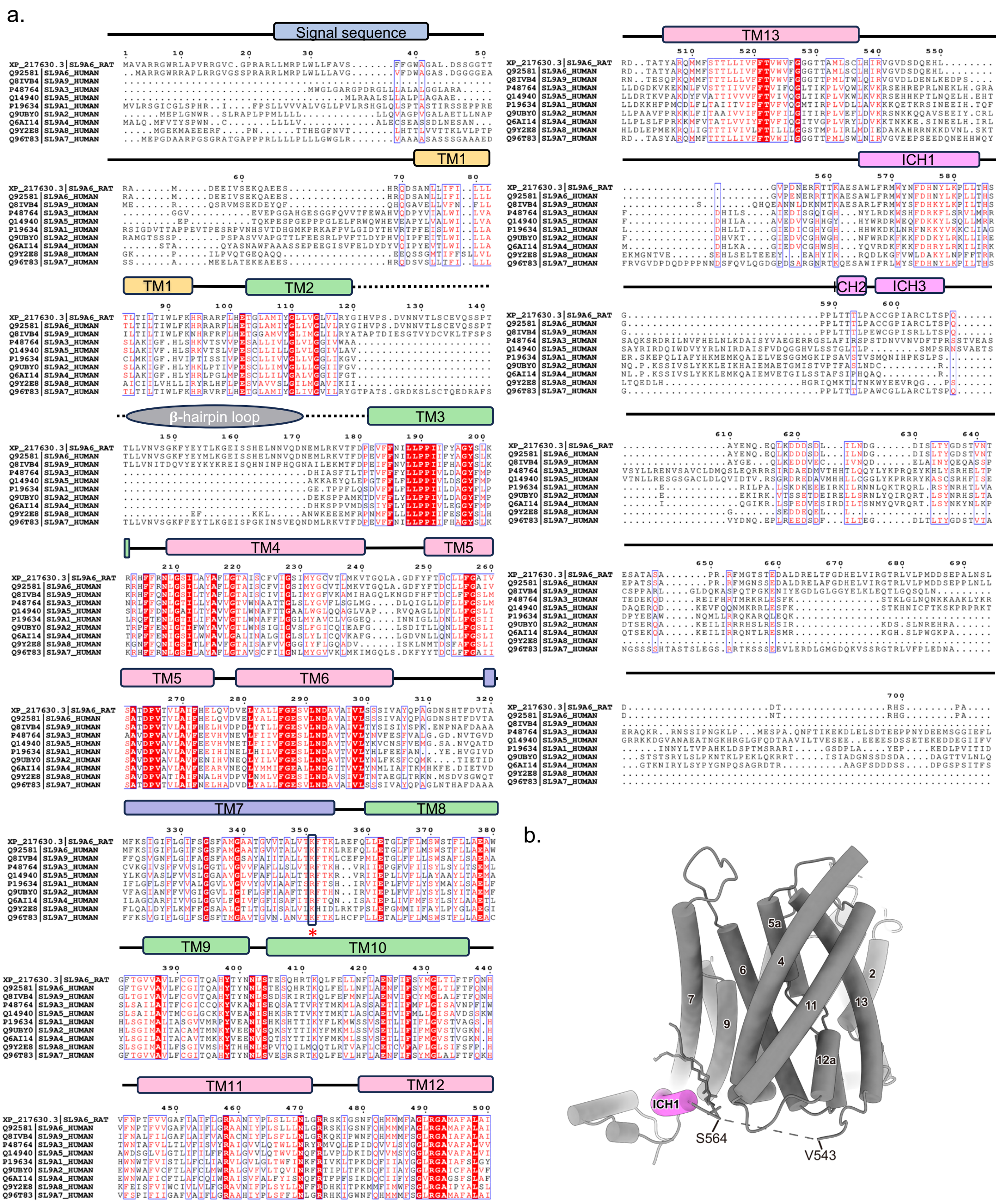

Supplementary Figure 2. Multiple sequence alignment and topology for rat NHE6. **a.** The following are the accession IDs for NHE1 (P19634), NHE2 (Q9UBY0), NHE3 (P48764), NHE4 (Q6AIL4), NHE5 (Q14940), NHE7 (Q96T83), NHE8 (Q9Y2E8), NHE9 (Q8IVB4), human NHE6 (Q92581) and rat NHE6 (XP_217630.3, isoform X1). Residues with over 90% sequence identity are indicated by red background. Residues which form TMs (coloured), loop (grey) and C-term helix (pink) are indicated. Dashed line represents regions that could not be built in the rat NHE6 structures. The Red asterisk highlights the strictly conserved lysine or arginine residue in TM7 that forms

the auto-inhibition interaction with the CTD. **b.** Cartooon representation of the rat NHE6 monomer structure in detergent (grey) highlighting that twenty one loop residues (V543 to S564, illustrated by the dashed line) connecting the end of TM13 with the beginning of ICH1 (pink) in the CTD that could not be built.

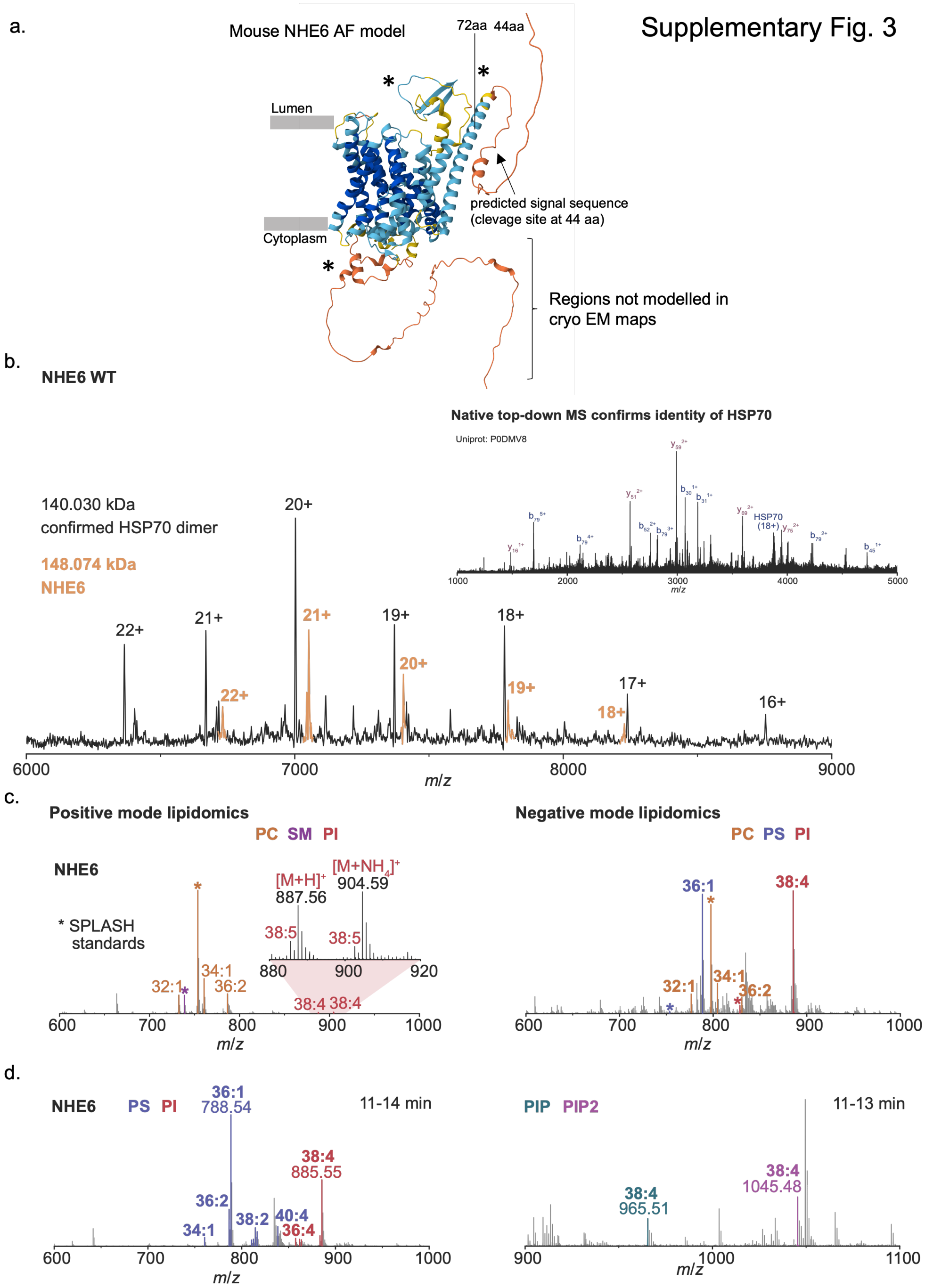

Supplementary Figure 3. AlphaFold2 prediction and Mass Spectrometry of rat NHE6. a. AlphaFold 2 model of the rat NHE6 monomer that showing the predicted position of signal sequence at the N-terminus (predicted cleavage site at residue 44). Colour scale from low confidence (orange) to high confidence (dark blue). Asterisk highlights regions that could not be modelled in the NHE6 cryo EM structures. **b.** Native mass spectrometry of purified rat NHE6 WT reveals a protein of 148 kDa, which is close to the expected mass of the signal peptide-cleaved NHE6 dimer (147.8 kDa).

The signal overlaps with the HSP70 dimer. The spectrum was obtained in 200 mM ammonium acetate, 2xCMC C8E4. *Insert:* Native top-down mass spectrum of HSP70 monomer. The identity of HSP70 was confirmed by higher-energy collisional dissociation (HCD) of the 18+ monomer charge state. The most abundant b- and y-fragments corresponding to Uniprot entry P0DMV8 are assigned in the spectrum. **c.** Standard lipidomics of rat NHE6 WT in positive and negative mode. SPLASH II Lipidomix standard was added to probe for enrichment or depletion of certain lipid classes. Phosphatidylserine (PS) and phosphatidylinositol (PI) lipids 18:0_20:4 were enriched in the WT, whereas amounts of PC was reduced relatively. **d.** Targeted negative-mode lipidomics. PI, PS, PIP and PIP2 copurified with rat NHE6 WT, as quantified from triplicate measurements. Representative spectra are shown.

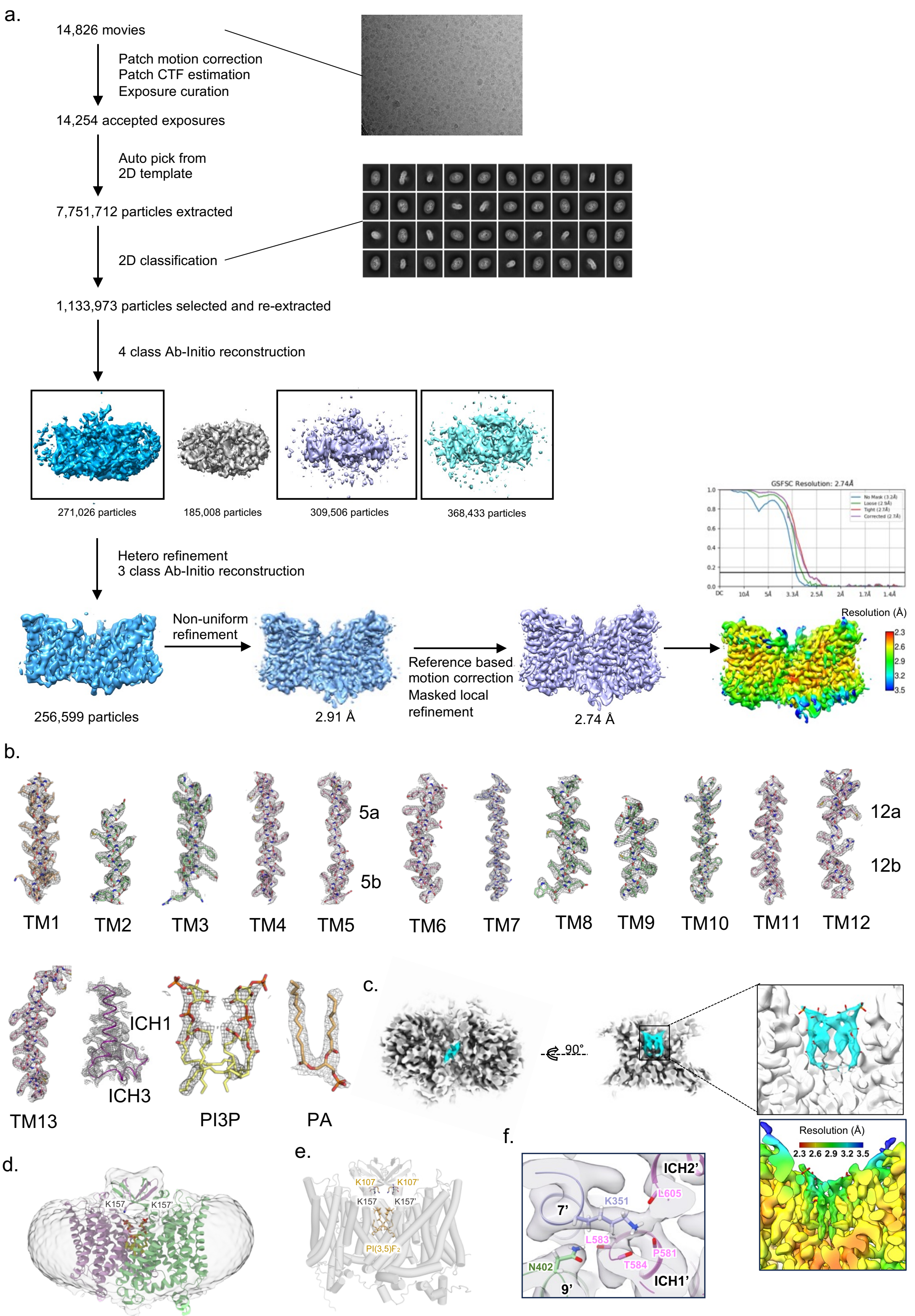

Supplementary Figure 4. The data-processing workflow of rat NHE6 with PI3P in detergent. a. The dataset contained 14,826 movies that were corrected by Patch Motion Correction and Patch CTF Estimation. After reference-based auto-picking, 7,751,712

particles were picked. Several rounds of 2D classifications yielded in good 2D classes of 1,133,973 particles. 3D classification and 3D refinement of model resulted in an electron density map of 2.91 Å. The electron density map was subjected to non-uniform refinement local refinement and reference based motion correction. A final resolution of 2.74Å was achieved at gold-standard FSC (0.143), with a local resolution range of 2.3–3.5 Å**.** **b** Cryo-EM density map and model are shown for all the transmembrane segments for the NHE6 structure in detergent with the dimer domain (pale green), transport domain (light pink), PI3P lipid (yellow) and PA lipid (light orange) all shown as sticks. **c.** Cryo-EM map densities for lipids (cyan) at dimer interface of rat NHE6 in detergent (extracellular view and front view). **d.** Cartoon representation of the rat AF3[42] homodimer model of rat NHE6 (purple, green). The cryo EM maps prior to masked refinement shows features for the β-hairpin loop domain, which could not be built into the final cryo EM maps due to poor map quality **e.** Cartoon representation of the horse NHE9 structure (grey) with $PI(3,5)P_2$ bound (organge sticks) and the β-hairpin domain modelled (PDB: 8PXB). In NHE9, two lysine residues K107 and K109 (tan sticks) are important for $PI(3,5)P_2$ binding and one of these lysine residues (K157) is conserved in NHE6 (black sticks). **f.** Cryo-EM density map and model at the TM7-ICH1-ICH2 interaction.

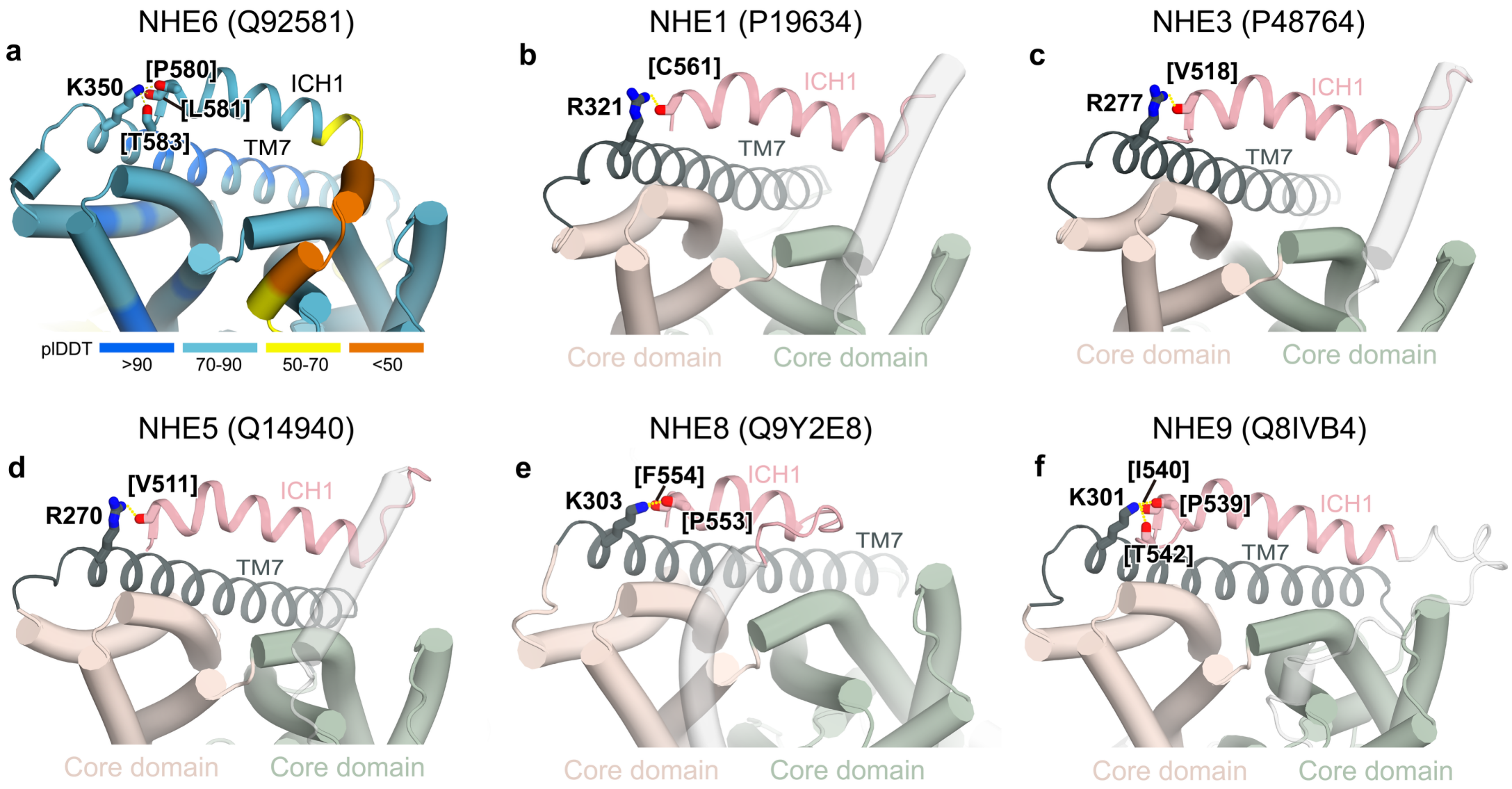

Supplementary Fig. 5. AlphaFold 3 predictions of interactions between the CTD and transporter module of representative NHE members. **a**. AF3[42] Cartoon representation of the backbone interactions between the end of ICH1 and the lysine residue (K350) in TM7 in human NHE6 (Uniprot: Q92581). Helices are colored based on the plDDT score as illustrated by the color scale and shown as cartoon, and residues of importance as sticks. The NHE6 dimer was predicted by AF3, but only one monomer is shown for sake of clarity. **b**. As in a., for NHE1 yet colored according to scaffold (pale pink) or core (pale green) domain. The plDDT scores correlate with the human NHE6. **c-f.** As in b. for human NHE3, human NHE5, human NHE8 and human NHE9, respectively.

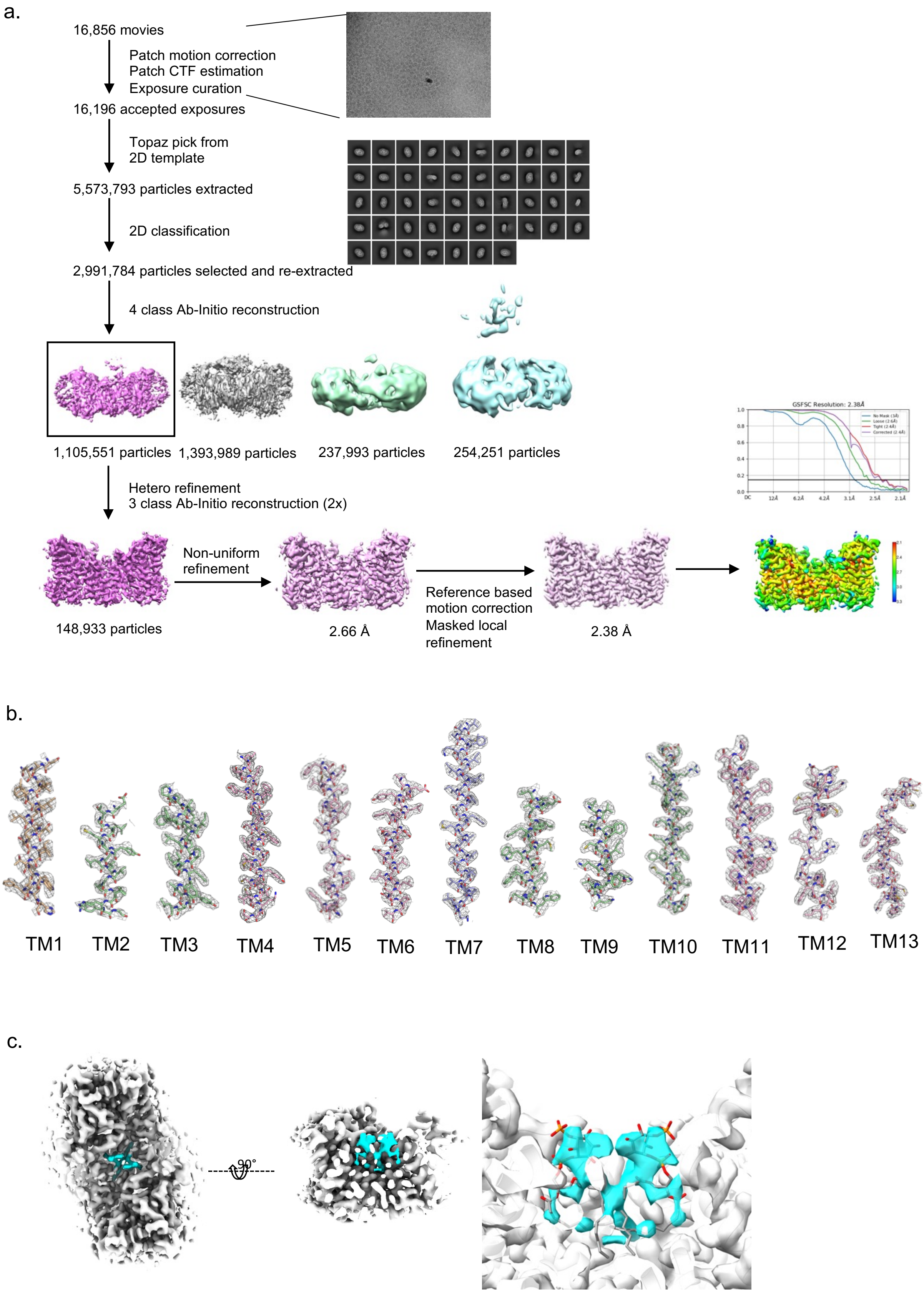

Supplementary Figure 6. The data-processing workflow of the NHE6 K351A variant in detergent PI3P. a. The dataset contained 16,856 movies that were corrected

by Patch Motion Correction and Patch CTF Estimation. After reference-based picking with Topaz 5,573,793 particles were picked. Several rounds of 2D classifications yielded in good 2D classes of 2,991,784 particles. 3D classification and 3D refinement of the model resulted in an electron density map of 2.66 Å. The cryo EM maps were subjected to non-uniform refinement, local refinement and reference based motion correction. A final resolution of 2.38Å was achieved at gold-standard FSC (0.143), with a local resolution range of 2.1–3.3 Å. **b** Cryo-EM density map and model are shown for all the transmembrane segments for K351A structure in detergent with the dimer domain (pale green), transport domain (light pink) **c.** *left:* Cryo-EM map densities of K351A in detergent, with lipid densities observed at dimer interface of K351A highlighted in cyan (extracellular view and front view). *right*: magnified view of the front view from left with the modelled lipids shown as sticks.

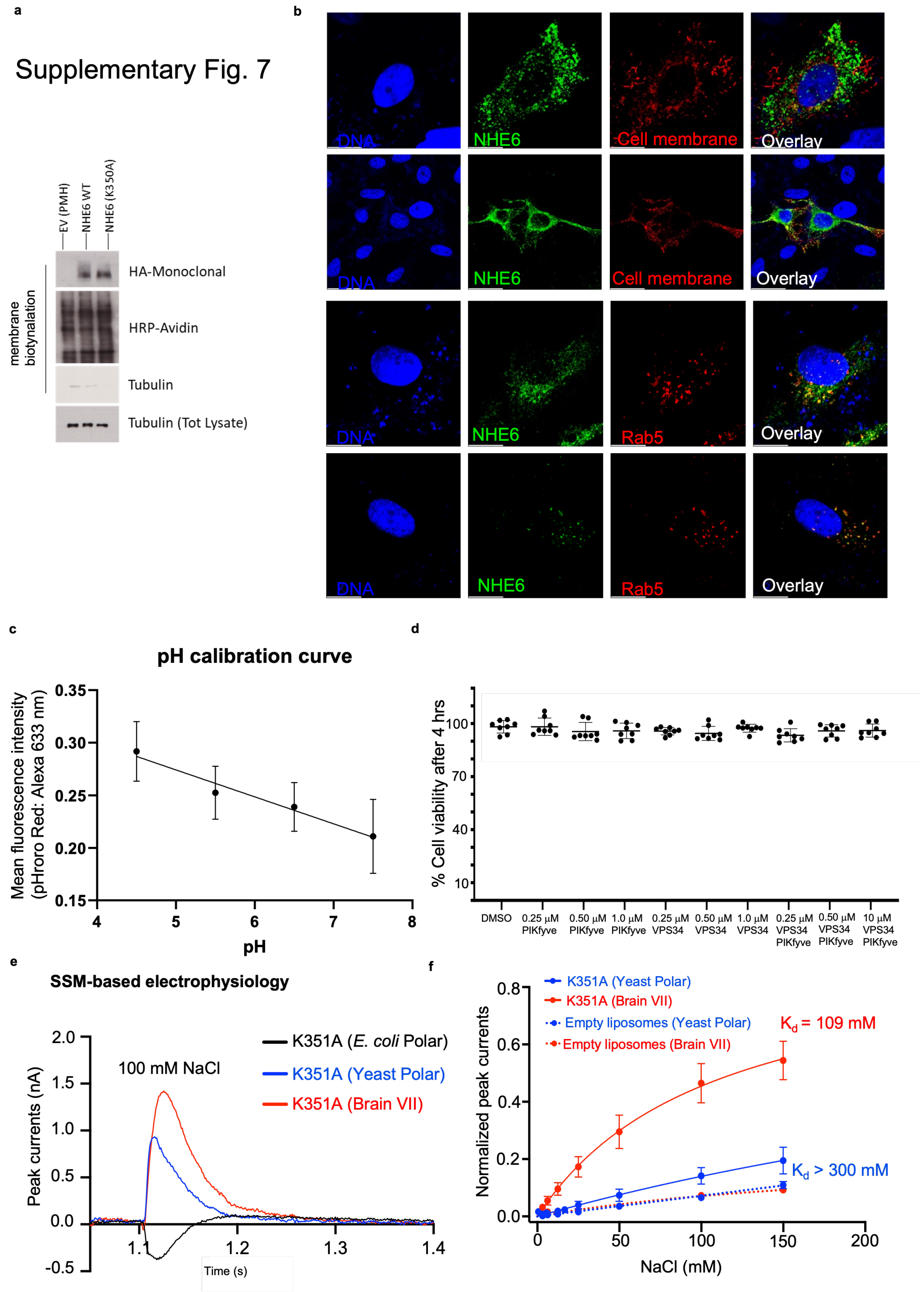

Supplementary Figure 7. SSM electrophysiology for NHE6WT. a. Plasma membrane expression of HA-tagged NHE6 and the NHE6 K350A variant (rat NHE6 K351A) in PS120 cells. PS120 were transfected with empty vector (EV; pMH), human NHE6 or human NHE6 K350A. Plasma membrane proteins were isolated using surface biotinlyation. Avidin–HRP was used as loading control. The absence of a strong tubulin signal confirms the purity of the membrane fraction. Three independent biological

replicates per condition. **b**. Representative immunofluorescence images of PS120 cells expressing human NHE6. Cells were fixed in 4% PFA and NHE6::HA visualized by labelling HA tag with immunoflouresence (green). Cell membranes were visualized using Cytoliner dye (red, bottom row) while endosomes were labelled with Rab5 (red, upper and middle row) and nuclei counterstained with Hoechst 33342 (blue). Images were acquired using a Leica SP8 confocal microscope. **c.** pH calibration curve for endosomal pH measurements for human NHE6**.** Error bars are the mean values ± s.e.m. of: n = 3 individual repeats **d**. Cell viability following phosphoinositide kinase inhibition was assessed using the alamarBlue™ HS Cell Viability Reagent. Treatments were performed for 4 h to assess acute cytotoxic effects. n = 8 individual repeats. **e**. Representative transient currents recorded for K351A proteoliposomes in liposomes made from either brain VII extract (red), yeast polar lipids (blue) or *E. coli* lipids (black) upon addition of 150 mM NaCl. **f.** Transient currents were recorded for K351A in liposome made from either brain VII extract (red) or yeast polar lipids (blue) extract with increasing Na^+ concentration. Empty liposomes were measured as control. Error bars are the mean values ± s.e.m. of: n = 3 individual experiments. Source data are provided as a Source Data file.

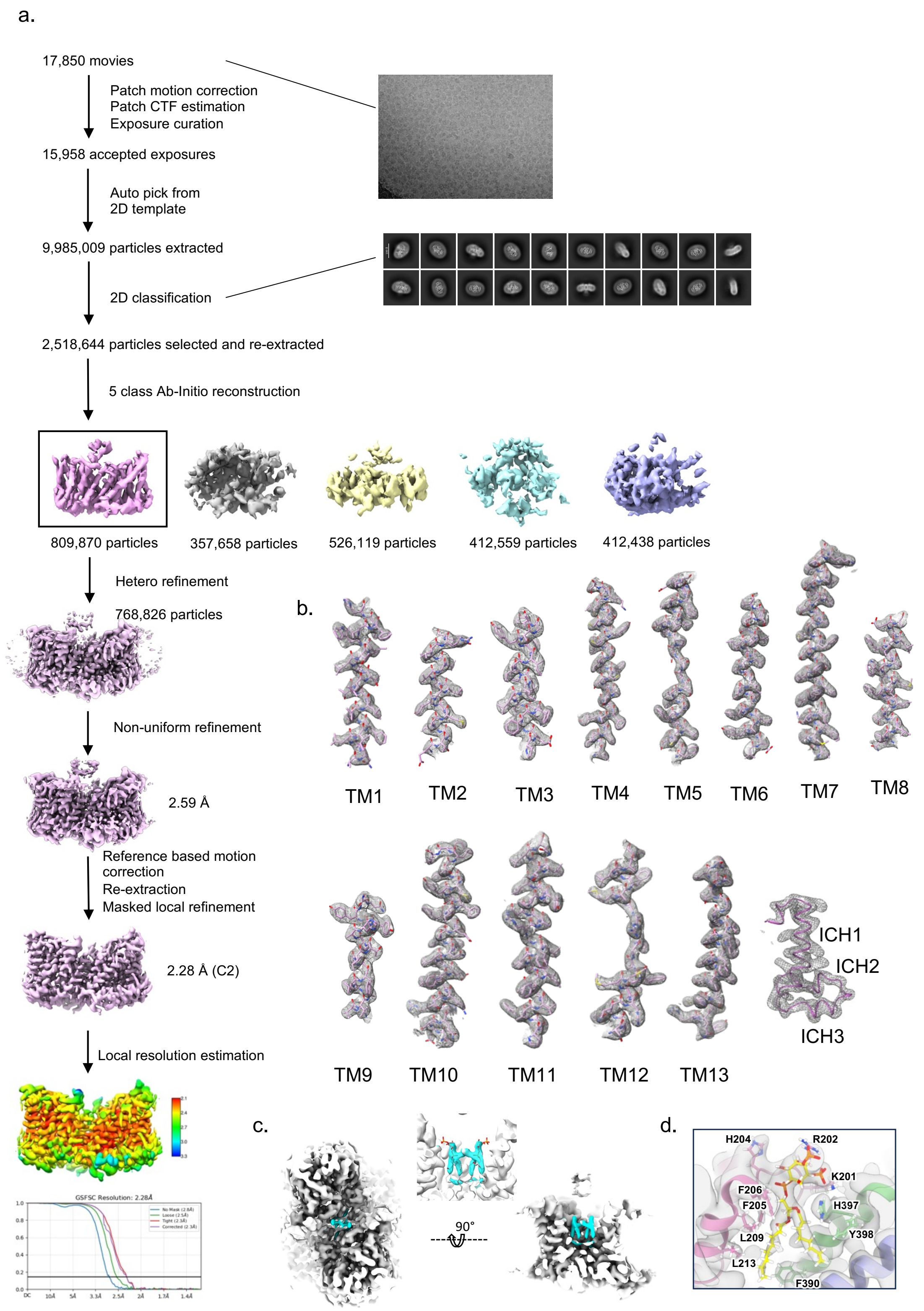

Supplementary Figure 8. The data-processing workflow of NHE6 D293A in complex with PI3P in detergent. a. The dataset contained 17,850 movies that were corrected by Patch Motion Correction and Patch CTF Estimation. After reference-based auto-picking, 9,985,009 particles were picked. Several rounds of 2D classifications

yielded in good 2D classes of 2,518,644 particles. 3D classification and 3D refinement of the model resulted in an electron density map of 2.59 Å. The electron density map was subjected to non-uniform refinement local refinement and reference based motion correction. A final resolution of 2.28Å was achieved at gold-standard FSC (0.143), with a local resolution range of 2.1–3.3 Å. **b.** Cryo-EM density map and model are shown for all the transmembrane segments for the D293A structure in complex with PI3P in detergent with the dimer domain (pale green), transport domain (light pink) **c.** *left:* overall cryo-EM map density with densities corresponding to the PI3P lipids highlighted in cyan, located at dimer interface (extracellular view and front view). *right:* magnified view of the front view from left with the modelled lipids shown as sticks. **d.** Cryo-EM density map and model at the PI(4,5)P_2 binding site.

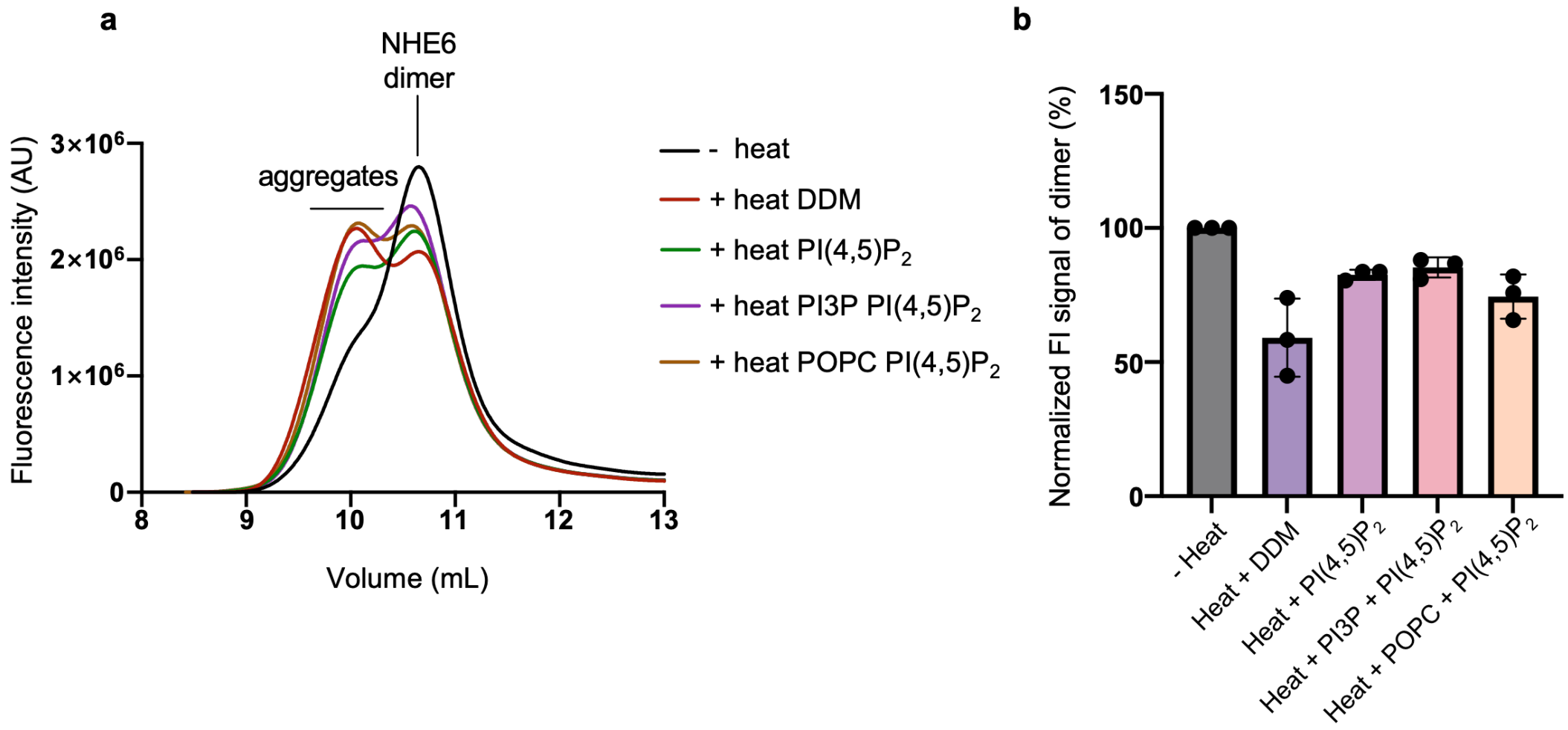

Supplemntary Fig. 9. Thermostabilization of rat NHE6 with PI(4,5)P2 lipids and relative competition by PI3P and POPC lipids. **a.** Representative FSEC traces of NHE6-GFP at 4°C and heated at 42°C with the addition of different DDM-solubilised lipids combinations. **b.** FSEC trances showing normalized percentages of NHE6 WT dimer remaining after heating in the presence of different DDM-solubilised lipids. Error bars represent the s.d. ± mean from n =3 independent experiments. Source data are provided as a Source Data file.

a

NHE6 mutations associated with Christianson Syndrome

Mutation/ Trucation	RnSLC9A6	HsSLC9A6	Reference
W89R	W90	W89	[66]
M106V	M107	M106	[67]
L188P	L189	L188	[68]
P189L	P190	P189	[69]
G218R	G219	G218	[70]
G383D<br>G383D<br>G383S	G384	G383	[14]<br>[71]<br>[68]
G448R	G449	G448	[72]
R568Q	R569	R568	[68]
E611K	E612	E611	[73]
L616F	L617	L616	[74]
ΔV176-R201	V177-R202	V176-R201	[8]
ΔE255-S256	E288-S289	E255-S256	[8]
ΔW369-T371	W371-T373	W369-T371	[75]
ΔL413-T450	L414-F451	L413-T450	[76]

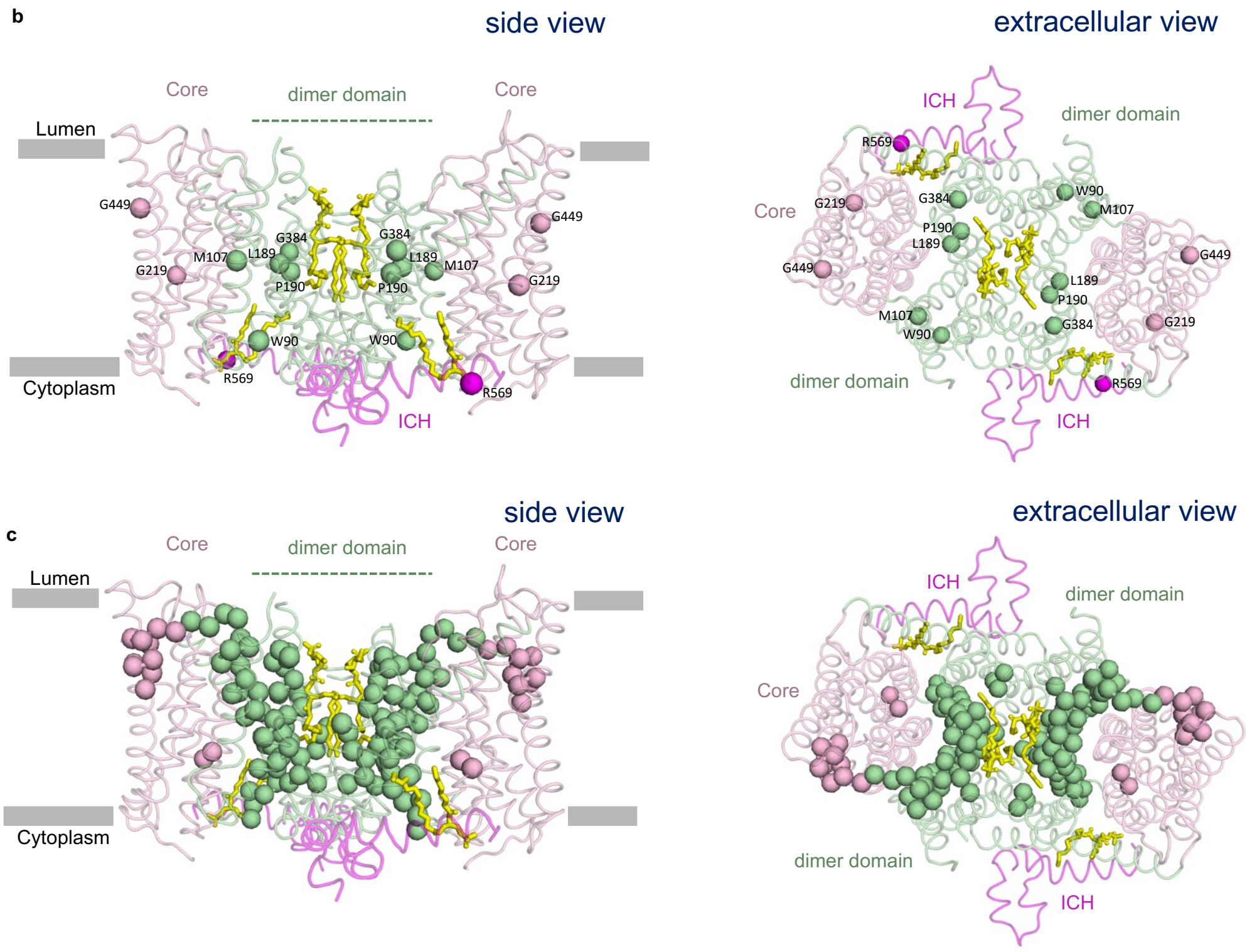

Supplementary Fig. 10. Mutations in human NHE6 causing Christianson Syndrome. a. Table of single point mutant and/or deletion in rat NHE6 and corresponding residues in human NHE6 causing the rare genetic X-linked disorder. **b**. Position of residue mutations (spheres) in rat NHE6 related to the rare genetic disease mapped onto the structure in the side (left) and extracellular (right) views, with the protein shown as cartoon colored as in Fig. 1a with PI3P lipid (yellow sticks). **c.** As in b., but spheres indicate the locations of deletion mutation. Most mutations are clustered in the dimerization domain.

Supplementary Table 1: Cryo EM processing and refinement statistics

	#1 NHE6-nanodisc (EMDB-54661) (PDB 9S8E)	#2 NHE6-WT (EMDB-54660) (PDB 9S8D)	#3 NHE6-K351A (EMDB-54662) (PDB 9S8G)	#4 NHE6-D293A (EMDB-54659) (PDB 9S8C)
**Data collection and processing**				
Magnification	130,000	130,000	130,000	130,000
Voltage (kV)	300	300	300	300
Electron exposure (e^{-}/Å^2)	63	57	58	53
Defocus range (μm)	0.4-2.3	0.4-2.3	0.4-2.0	0.4-2.0
Pixel size (Å)	0.66	0.65	0.65	0.65
Symmetry imposed	C2	C2	C2	C2
Initial particle images (no.)	3,288,197	7,751,712	5,573,793	9,985,009
Final particle images (no.)	127,174	256,599	148,933	767,151
Map resolution (Å)	3.25	2.74	2.38	2.28
FSC threshold	0.143	0.143	0.143	0.143
Map resolution range (Å)	2.7-3.9	2.3-3.5	2.1-3.3	2.1-3.3
**Refinement**				
Initial model used (PDB code)	Alphafold2 dimer	NHE6-nanodisc	NHE6 WT	NHE6 WT
Model resolution (Å)	3.4	2.9	2.6	2.4
FSC threshold	0.5	0.5	0.5	0.5
Model composition				
Non-hydrogen atoms	7,096	7,638	6,862	7,932
Protein residues	896	948	850	956
Ligands	-	PI3P, Na^+, PA	PI3P, Na^+, POPC	PI3P, PI(4,5)P_2, POPC, PE
B factors (Å^2)				
Protein	133	96	101	115
Ligand	-	121	124	134
R.m.s. deviations				
Bond lengths (Å)	0.002	0.003	0.002	0.004
Bond angles (°)	0.440	0.482	0.406	0.596
Validation				
MolProbity score	1.43	1.42	1.06	1.28
Clashscore	3.78	5.17	2.68	5.18
Poor rotamers (%)	0.27	0.51	0.43	0.63
Ramachandran plot				
Favored (%)	96.2	97.2	98.8	98.4
Allowed (%)	3.9	2.8	1.2	1.6
Disallowed (%)	0	0	0	0

Supplementary Table 1. Cryo-EM data collection and refinement statistics for rat NHE6 structure in nanodisc and detergent, K351A and D293A variants.

	NaCl (Kd, mM)							KCl (Kd, mM)					
	Yeast Polar	Brain VII						Brain VII					
	K351A	K351A	K351A +PI3P	K351A +PI35P2	K351A +PI45P2	WT	WT+PI3P	K351A	K351A +PI3P	K351A +PI35P2	K351A +PI45P2	WT +PLC	WT +PLC+ PI3P
Sensor #1	> 300	109	116	> 200	> 200	> 200	> 200	94	41	89	146	6	10
Sensor #2		87	93	179	> 200	> 200	> 200	85	39	117	148	20	26
Sensor #3		107	97	> 200	> 200	> 200	> 200	76	85	92	150	28	36
Sensor #4		80	101	> 200				56			125	19	23
Sensor #5		89											
**Kd (mM)**	**> 300**	**95 ± 6**	**102 ± 5**	**> 200**	**> 200**	**> 200**	**> 200**	**77 ± 6**	**55 ± 15**	**99 ± 9**	**142 ± 6**	**18 ± 5**	**24 ± 5**

Supplementary Table 2. Summary of the average apparent binding affinities (K_d^{app}) for NHE6 WT and NHE6 K351A in either the presence of different lipid compositions or after incubation with phospholipase C.

Supplementary Movie 1. Movie of the 3D Variability Analysis (3DVA) carried out in cryoSPARC on the cryo-EM map reconstruction of NHE6 WT in detergent with PI3P and processed in C1 symmetry.
