# Supplementary material for: Structure of NHE6 and its lipid-mediated interactions regulating endosomal pH
Source: Nat Commun. 2026 Aug 11;17:8177. doi: 10.1038/s41467-026-75877-x (PMC13463026; doi:10.1038/s41467-026-75877-x)
Supplement: Supplementary file 2 [file 41467_2026_75877_MOESM2_ESM.pdf]

Title: Supplementary Movie 1

Descripton: Movie of the 3D Variability Analysis (3DVA) carried out using cryoSPARC on the cryo-EM map reconstruction of NHE6 WT in detergent with PI3P and processed with C1 symmetry.
