# Supplementary material for: Structure of NHE6 and its lipid-mediated interactions regulating endosomal pH
Source: Nat Commun. 2026 Aug 11;17:8177. doi: 10.1038/s41467-026-75877-x (PMC13463026; doi:10.1038/s41467-026-75877-x)
Supplement: Supplementary file 4 [file 41467_2026_75877_MOESM5_ESM.pdf]

nature portfolio

Peer Review File

Structure of NHE6 and its lipid-mediated interactions regulating endosomal pH

Corresponding Author: Professor David Drew

This file contains all reviewer reports in order by version, followed by all author rebuttals in order by version.

Version 0:

Reviewer comments:

Reviewer #1

(Remarks to the Author)
The structure of NHE6 has not been resolved despite its importance in endosomal pH regulation and human diseases. The manuscript by Jung et al is an exemplary manuscript demonstrating the cryo-EM of rat NHE6 for the first time. The quality and the rigor of the experiments are exceptional. The impact of the study is high given the importance of this molecule to brain disease.

In this study, Jung et al. revealed the cryo-EM structure of rat NHE6. Similar to the NHE9 structure, NHE6 forms homodimer. NHE6 is a 13-TM protein predicted with its N-terminus located in the luminal side and its C-terminus located in the cytoplasmic side. They further revealed that PI3P, PI(3,5)P2, PI(4,5)P2 lipids bind to NHE6, but the authors propose that the lipid interactions have distinctive regulatory roles for NHE6. Specifically, PI3P (which is endosome enriched) binding at the dimer interface stabilizes the homodimer conformation of NHE6 and enhances activity. The authors propose that PIP2 binding at the C-terminal domain promotes the auto-inhibition state, inhibiting the exchange function. This is reasonable as PI3P/PI(3,5)P2 are abundant on endosomal membrane, while PIP2 is more on the plasma membrane. Additionally, they showed K351 in TM7 interacts with a conserved helix (ICH) in the CTD of NHE6 to maintain the auto-inhibition state of NHE6. Their findings are mostly agreed with the previous reports e.g., D293 as a key residue for ion binding, but at the same time, this is the first well-defined study on the NHE6 structure. This significant work connects the function of lipids to endolysosomal pH regulation. A few key points, including suggestions for marginal improvements in some of the functional assays and their interpretations, should be addressed along the minor comments before the publication – some of these comments regard the complexity of the biology and inherent limitations in data interpretation. The manuscript has many strengths. Among these strengths is that the data are very clearly and rigorously presented. The direct comparison with the prior work on CryoEM for NHE9 is a major strength. The investigators generated distinct NHE6 mutations, including the K351A mutation. These mutant NHE6 mutations are very strong additions to the data. There are also a number of very important observations from the data, including the proposal that under physiological states that NHE6 is likely to operate as a K+ exchanger with H+ (as opposed to Na+). Furthermore, the experimentation with the D293A variant is a major strength which has permitted the investigators to map the position of a bound ion to NHE6. Overall, we have a very high level of enthusiasm for the study.

Major comments:
1. The authors showed that NHE6 is able to bind different types of lipids including PI3P, PI(3,5)P2 and PI(4,5)P2, and these lipid-mediated interactions have functional impacts on NHE6. Lipodomics and other assays suggest that NHE6 preferably binds to PI3P than other two lipids. However, did the authors quantify the differential binding affinity of PI3P, PI(3,5)P2 and PI(4,5)P2 to NHE6? I may have missed this.
2. Figure 1e and 1F showed brain lipids, PI3P, and PI(3,5)P2 stabilize the NHE6 dimer. This assay might additionally include PI(4,5)P2, considering its functional importance for the plasma membrane.
3. The C-terminus of NHE6 is known to be disordered except a conserved helix (ICH), including the reports from a recent preprint (Feilen et al.) on the NHE6 structure. It is uncertain how the authors overcome this challenge in this study. If the authors were able to resolve the full structure of CTD, this specific should be included in the method. If not, the discussion about this disordered region should be included.
4. The authors include cellular functional assays to test whether NHE6 activity is inhibited on the plasma membrane where PI(4,5)P2 is abundant (Figure 3b-d), additional to these exciting structural works. The authors present biochemical evidence

of surface expression; however, as a control, it might be important for the authors to present the images of cells expressing each construct (WT, K350A, NHE1, empty) to determine their cellular distribution (plasma membrane vs. endosomes) using microscopy, although this experiment may not be essential.
5. The authors may discuss if the complete lack of NHE activity on the surface was expected for the K350A variant. This may be construed as somewhat inconsistent with their model that the cytoplasmic tail is auto-inhibitory as the K350A variant is proposed to remove the auto-inhibitory interaction. I believe that the authors may argue that the surface contains inhibitory lipids, ie PI(4,5)P2; however, to what extent do the authors understand the mechanism of this inhibition? The authors might consider discussing what is known and/or speculating on this in the Discussion. To this extent, while the authors propose that PI(4,5)P2 inactivates NHE6 on the plasma membrane (Fig 3), could they test this functionally with inhibitory lipids and with drugs that may inhibit the formation and/or localization of these lipids?
6. In Fig 3d, would the authors consider doing the endosomal pH measurement with and without PIP inhibitors to demonstrate the functional effect of these lipids on NHE6 activity?
7. In Figure 3e, will be WT less active in the presence of PI(4,5)P2? The authors may consider a control whereby they test whether PI(4,5)P2 inactivates wild-type NHE6.
8. Even though PI3P and PI(3,5)P2 stabilizes the NHE6 dimer in Figure 1f, the authors suggest that they have the opposite roles on regulating NHE6 in Figure 4f. Is there a discrepancy here? Please could the authors put a comment on this in the discussion, to the extent that they understand this biochemistry, about structural stabilization and functional impacts should be included.

Minor comments:
1. The following sentence is not so clearly written: “The unusual repositioning of TM1 weakens the interface surface area of the homodimer from 1500 A2 in NHE9 to only 1100 A 2in NHE6 (Methods).” I believe I understand what the authors mean here, but I wonder if they broke down the concepts here into two sentences, the information may be expressed more clearly.
2. Not sure Figure 7 schematics was introduced in the text.
3. No supplementary figure 2a was found for showing the variation of CTD among NHE isoforms.
4. Line colors for Fig 1e are not visibly distinguishable, especially between samples with brain lipids vs. samples with e.coli polar lipids.
5. In figure 3e-g, it is confusing that WT was tested with PI3P from brain lipids, while K351A was tested with brain lipids. Was the peak current of K351A different from WT in the presence of PI3P?
6. PIP2 vs. PI(4,5)P2 seems interchangeably used, but it is unclear throughout the text and figure legends, and PI(3,5)P2 are distinct.

Reviewer #2

(Remarks to the Author)

In general, the structural work is careful, and the lipid-regulation model is intriguing. To fully support the mechanistic conclusions, several additional experiments would strengthen the paper, as outlined below.

1.Lipid-dependent regulation claims (PI3P at the dimer interface; PIP2 at the CTD): The central claim is that PI3P stabilizes the dimer interface and enhances activity, whereas PIP2 engages the CTD to enforce auto-inhibition and silence NHE6 at the plasma membrane. Much of this rests on density assignments and indirect functional readouts. Suggestions: (1) Reconstitute into liposomes with lipid compositions (±PI3P, ±PI(3,5)P2, ±PIP2; ±cholesterol) and quantify functional activity. (2) Mutate key contacts at the interface (e.g., N74, W371/β-hairpin Lys) and at the CTD–core latch (e.g., K351 and basic residues near the modeled PIP2 pocket) and test effects on stability and transport. (3) Provide local map-model validation around the lipid densities.

2.Ion-binding site identity and evidence: The Na^+ site is modeled with supportive data (D293A abolishes the extra density), but chemical identity/occupancy is still inferred. Suggest doing a contour analysis to rule out that the density is water.

3.Na^+ vs K^+ selectivity and kinetics: SSM measurements rely primarily on K351A and suggest similar millimolar Kd’s for Na^+ and K^+ with PI3P-dependent enhancement. It is concluded that K^+ is the transported ion. It would be more physiologic to perform Na^+-K^+ competition studies by measuring the dose response to K^+ in the presence of physiologic Na^+ and vise versa since they compete. These data would provide the effective Kd for each ion. I would also add inhibitor controls (amiloride-class) to confirm NHE-specific exchange in the reconstitution used for SSM.

4.CTD auto-inhibition: correlation vs causation: Structures and cell data argue the CTD latches onto TM7 (K351) and that PIP2 stabilizes an off-state; however, direct functional causality needs clearer results. Would test ΔC1/point mutants at the CTD–TM7 interface (e.g., L581/L582↔TM7 Lys) for effects on activity and conformational bias.

5.Plasma-membrane inactivity experiments: Consider also depletion of plasma membrane PIP2 as a control.

6. The pH-responsive pHrodo™ Red transferrin endosomal pH measurement: The resolution of the assay seems poor given the small changes in the ratio between pH 7.0 and 4.5. Other than these extremes, how accurate is the measurement?

Reviewer #3

(Remarks to the Author)
I co-reviewed this manuscript with one of the reviewers who provided the listed reports. This is part of the Nature Communications initiative to facilitate training in peer review and to provide appropriate recognition for Early Career Researchers who co-review manuscripts.

Reviewer #4

(Remarks to the Author)
I co-reviewed this manuscript with one of the reviewers who provided the listed reports. This is part of the Nature Communications initiative to facilitate training in peer review and to provide appropriate recognition for Early Career Researchers who co-review manuscripts.

Reviewer #5

(Remarks to the Author)
- What are the noteworthy results?
The manuscript includes one figure (S3) with native mass spectrometry data and lipidomics work. The portion of the mass spectrum shown shows the signals for NHE6 dimer overlapping with HSP70 dimer. HSP70 was identified by MSMS analysis of the HSP70 monomer (shown inset). The NHE6 protein did not need to be pure for cryo-EM as other proteins can be ignored in the analysis. The lipidomics is adequate.

- Will the work be of significance to the field and related fields? How does it compare to the established literature? If the work is not original, please provide relevant references.
The work is of some significance to the analysis but placement in supplementary data is probably adequate.

- Does the work support the conclusions and claims, or is additional evidence needed?
The work supports the conclusions made in the manuscript.

- Are there any flaws in the data analysis, interpretation and conclusions? - Do these prohibit publication or require revision?
The data analysis is not flawed with acceptable interpretation and conclusions.

- Is the methodology sound? Does the work meet the expected standards in your field?
The methodology is sound and meets the expected standards of the field.

- Is there enough detail provided in the methods for the work to be reproduced?
There is adequate detail in the methods. The origin of the time values in the top right of part D is unclear – they can be removed.

Version 1:

Reviewer comments:

Reviewer #1

(Remarks to the Author)
The authors have addressed all of my prior comments. Thank you for this important contribution to the field.

Eric M. Morrow PhD MD

Reviewer #2

(Remarks to the Author)
The CTD auto-inhibition mechanism is still not established with sufficient direct causality for the strength of the claim.

The authors rely mainly on the K351A mutant, the inability to model CTD density in that mutant structure, and the new PLC activation result to argue that PI(4,5)P2 stabilizes an auto-inhibited CTD-bound state. These findings do support the general model. However, the authors did not perform the additional interface-testing experiments that were suggested, and the rebuttal explicitly argues that one mutation is sufficient. Since CTD-based inhibition is one of the principal mechanistic conclusions of the paper, the remaining point is that the current data support a CTD-linked inhibitory model, but do not yet fully establish the precise CTD-TM7 inhibitory mechanism with the degree of certainty currently claimed.

Reviewer #3

(Remarks to the Author)

I co-reviewed this manuscript with one of the reviewers who provided the listed reports. This is part of the Nature Communications initiative to facilitate training in peer review and to provide appropriate recognition for Early Career Researchers who co-review manuscripts.

Reviewer #4

(Remarks to the Author)
I co-reviewed this manuscript with one of the reviewers who provided the listed reports. This is part of the Nature Communications initiative to facilitate training in peer review and to provide appropriate recognition for Early Career Researchers who co-review manuscripts.

Reviewer #5

(Remarks to the Author)
The author has addressed any concerns I had following my original review. I recommend proceeding with the publication process.

Version 2:

Reviewer comments:

Reviewer #2

(Remarks to the Author)
1. Lipid-dependent regulation: This point is significantly improved. The authors now provide better support that PI3P and PI(4,5)P2 have distinct functional effects. PI3P supports activity, whereas PI(4,5)P2 inhibits activity. The authors also added thermostability data and argue that PI3P does not bind tightly to the CTD PI(4,5)P2 site because PI3P was not observed there despite being added before blotting at high concentration. The PLC experiment is particularly important because it functionally connects PI(4,5)P2 removal with rescue of WT NHE6 activity. This is much stronger than relying only on static cryo-EM lipid density. The authors still did not perform defined lipid reconstitution experiments with controlled lipid compositions or mutagenesis of proposed lipid-contact residues. The lipid-regulation model is now much better supported, especially the inhibitory role of PI(4,5)P2. However, the precise lipid-specific mechanism remains incompletely tested at the residue level.

2. Na^+ versus K^+ physiological relevance: The authors now provide separate apparent affinity measurements for Na^+ and K^+ and argue that K^+ is more likely to be the physiologic counter-ion because intracellular/endosomal-facing K^+ concentration is much higher than Na^+. This is a reasonable interpretation, and the manuscript now frames the argument more clearly. The authors still did not perform Na^+/K^+ competition experiments under mixed-ion conditions. This is important because, in vivo, Na^+ and K^+ are not presented to the transporter separately. They compete in the same intracellular/endosomal cytoplasmic environment. The rebuttal says separate Kd measurements are standard and that competition experiments would provide IC50/Ki-type information, but this does not fully answer the physiological competition question. The data support NHE6 transport signals for both Na^+ and K^+ and make K^+ a plausible physiologic counter-ion. However, the conclusion that K^+ is the main physiological ion remains inferential rather than directly proven by mixed-ion competition experiments.

3. CTD auto-inhibition: This point is also significantly improved. The K351A mutant, loss of modeled CTD density in that mutant, activity of K351A in brain-lipid liposomes, and now PLC activation of WT NHE6 together provide a much stronger case that the CTD participates in auto-inhibition and that PI(4,5)P2 stabilizes the inhibited state. The authors also clarify that K351 interacts primarily with backbone atoms at the ICH1/ICH3 breakpoint, and they added a figure to better show the TM7–ICH1/ICH3 interaction. The authors still did not successfully provide additional functional tests of the CTD interface. They argue that K351A is sufficient because K351 is the main TM7 contact and because mutations in ICH1/ICH3 side chains might not disrupt a backbone-mediated interaction. They also state that a CTD deletion construct expressed poorly and could not be purified. That explanation is reasonable, but it does not fully replace direct reciprocal mutagenesis or a successful CTD truncation/interaction test. The CTD auto-inhibition model is now strongly supported. However, the exact CTD–TM7 interface mechanism is not exhaustively validated. It should be presented as a strong mechanistic framework, not as a completely proven residue-by-residue causal mechanism.

4. Plasma-membrane inactivity: The authors added immunofluorescence/localization data and confirm that NHE6-WT partially overlaps with surface dye and strongly marks RAB5-positive early endosomes. They also rely on surface biotinylation to support plasma-membrane expression of the constructs. They also address the apparent inconsistency that the K350A mutant remains inactive at the plasma membrane by arguing that CTD displacement alone may be insufficient and that endosomal PI3P and other CTD-associated factors may also be required. They did not directly deplete plasma-membrane PI(4,5)P2 in the cell-based assay. Instead, they used PLC treatment in proteoliposomes as the cleaner biochemical test of PI(4,5)P2 removal. That is useful, but it is not exactly the same as demonstrating the mechanism in cells at the plasma membrane. The plasma-membrane inactivity model is plausible and better supported than before, but the cellular PI(4,5)P2 mechanism remains indirectly supported rather than directly demonstrated in the plasma-membrane assay.

5. Ion-binding-site identity: The authors provide structural and comparative arguments supporting the modeled ion-binding

site, including the conserved location in the core domain and comparison to other NHE-family structures. The chemical identity of the density is still inferred. There is no direct anomalous validation, heavy-ion substitution, or functional mutagenesis of the coordinating residues specifically to validate the ion-site assignment. The ion-site assignment is reasonable, but it remains structurally inferred rather than directly validated chemically.

6. Disease mutations: The authors map Christianson syndrome mutations onto the structure and discuss how mutations may affect PI3P-dependent dimer stabilization, CTD regulation, or ion transport. They did not functionally test disease mutations. Therefore, the disease relevance is strengthened anatomically/structurally but not experimentally. The disease-mutation mapping is useful, but the functional consequences of specific patient variants remain untested.

Reviewer #3

(Remarks to the Author)
I co-reviewed this manuscript with one of the reviewers who provided the listed reports. This is part of the Nature Communications initiative to facilitate training in peer review and to provide appropriate recognition for Early Career Researchers who co-review manuscripts.

Open Access This Peer Review File is licensed under a Creative Commons Attribution 4.0 International License, which permits use, sharing, adaptation, distribution and reproduction in any medium or format, as long as you give appropriate credit to the original author(s) and the source, provide a link to the Creative Commons license, and indicate if changes were made.
In cases where reviewers are anonymous, credit should be given to 'Anonymous Referee' and the source.
The images or other third party material in this Peer Review File are included in the article's Creative Commons license, unless indicated otherwise in a credit line to the material. If material is not included in the article's Creative Commons license and your intended use is not permitted by statutory regulation or exceeds the permitted use, you will need to obtain permission directly from the copyright holder.
To view a copy of this license, visit https://creativecommons.org/licenses/by/4.0/

Corresponding authors:
David Drew

We appreciate the critique to improve our manuscript. We have carefully examined each comment and have responded to all points below.

Reviewers' Comments:
Reviewer #1 (Remarks to the Author):

Major comments:

1. The authors showed that NHE6 is able to bind different types of lipids including PI3P, PI(3,5)P2 and PI(4,5)P2, and these lipid-mediated interactions have functional impacts on NHE6. Lipodomics and other assays suggest that NHE6 preferably binds to PI3P than other two lipids. However, did the authors quantify the differential binding affinity of PI3P, PI(3,5)P2 and PI(4,5)P2 to NHE6? I may have missed this.

During purification we supplement NHE6 purification with crude brain lipids and the last step before analysis is a SEC run in detergent. The main lipids from lipidomics match PI3P and also PIP2. Although its not possible to directly measure affinities for lipids, it seems resonable to assume that PI3P and PIP2 bind better than others given their low concentrations and the fact they were not out-competeted by detergent during purification.

2. Figure 1e and 1F showed brain lipids, PI3P, and PI(3,5)P2 stabilize the NHE6 dimer. This assay might additionally include PI(4,5)P2, considering its functional importance for the plasma membrane.

Correct. We now include data to show that PI(4,5)P2 that, like PI(3,5)P2 can stabilise NHE6. So ligand-like lipids binding to either the dimer interface or the CTD stabilise NHE6. The main evidence we have is that PI3P site is different from the PI(4,5)P2 is that we have never observed PI3P lipids in the CTD even thought it was always added prior to structural analysis. This also includes the D293A structure where we could model PI(4,5)P2 at the CTD. We also have differences between their addition to functional activity. That is, addition of PI(4,5)P2 inhibits NHE6 activity whereas PI3P does not.

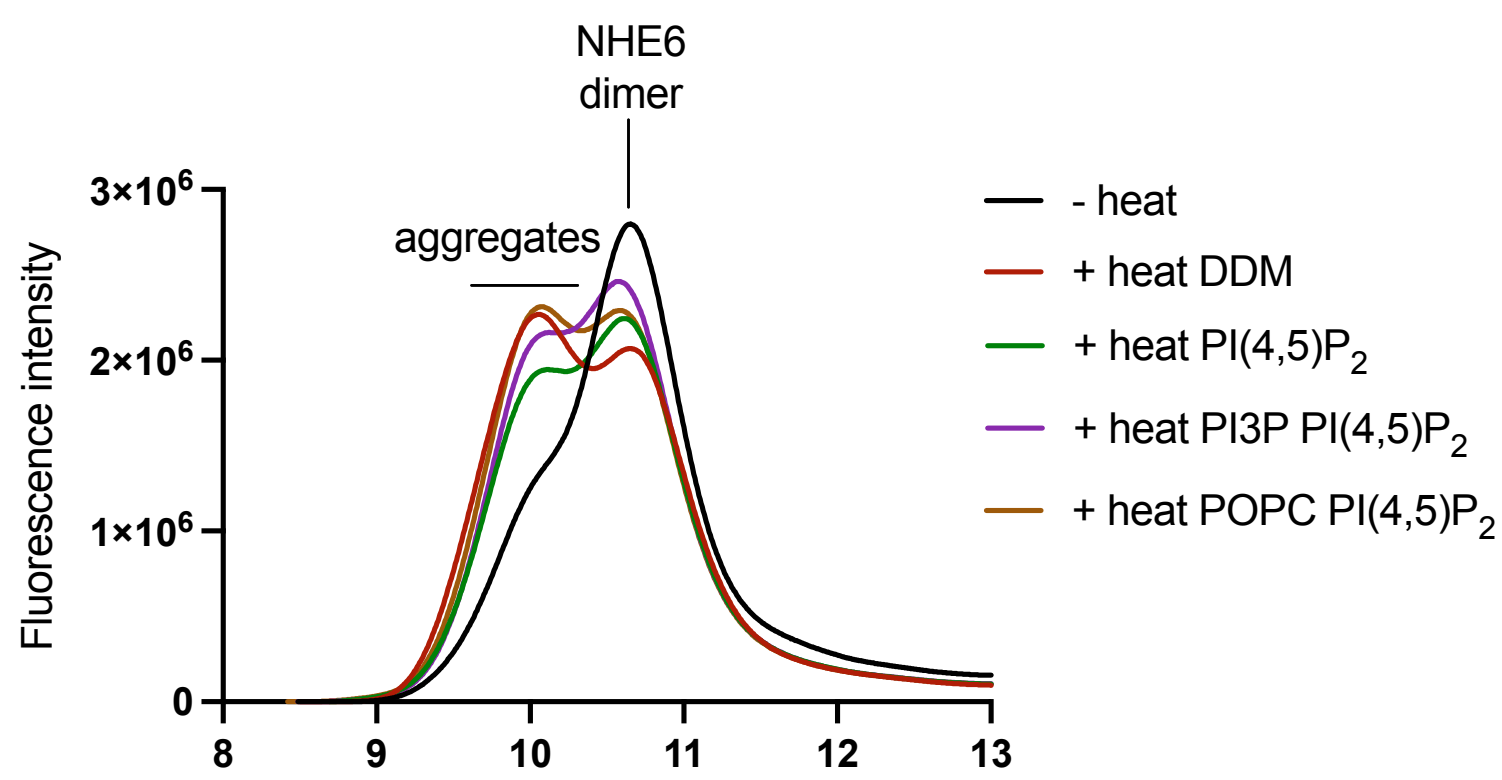

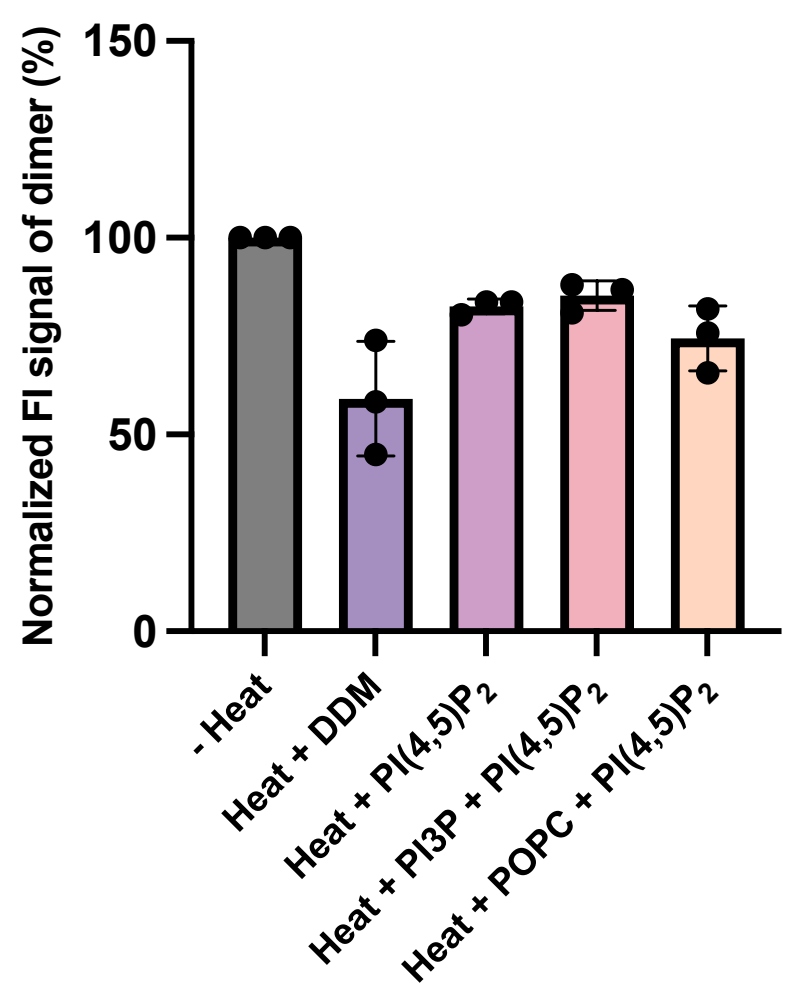

3. The C-terminus of NHE6 is known to be disordered except a conserved helix (ICH), including the reports from a recent preprint (Feilen et al.) on the NHE6 structure. It is uncertain how the authors overcome this challenge in this study. If the authors were able to resolve the full structure of CTD, this specific should be included in the method. If not, the discussion about this disordered region should be included.

The C-terminal tail is clearly flexible, but its only the very terminal regions that are disorded. AlphaFold2 is known to do a pretty good job at predicting disorder and it can clearly model ICH1 to ICH3 helices. In the nanodisc structure we could only model part of the C-terminal tail (ICH1) as we think the CTD is likely more flexible in yeast lipids. However, in all subsequent structures (apart from the K351A mutant) where we added brain lipids, we could model more of the CTD. Presumably the CTD will be more disordered in detergent, but the flexibility in detergent may not be physiologically relevant and lipid binding to the CTD can stabilise this domain. We did nothing different in terms of cryo EM processing. We think the paper is already clear on this point, but happy to add this discussion point explicitly if this reviewer thinks its needed.

4. The authors include cellular functional assays to test whether NHE6 activity is inhibited on the plasma membrane where PI(4,5)P2 is abundant (Figure 3b-d), additional to these exciting structural works. The authors present biochemical evidence of surface expression; however, as a control, it might be important for the authors to present the images of cells expressing each construct (WT, K350A, NHE1, empty) to determine their cellular distribution (plasma membrane vs. endosomes) using microscopy, although this experiment may not be essential.

We thank the reviewer for this thoughtful suggestion.

We performed immunofluorescence analysis for NHE6-WT and confirmed there is indeed partial overlap with the surface dye. Furthermore, RAB5-positive early endosomes that pinch off directly from the plasma membrane, are strongly positive (now shown in Supplementary Figure 6b). Importantly, our surface biotinylation experiments provide biochemical evidence for plasma membrane expression of the constructs used in the functional assays, which directly addresses the reviewer's concern regarding surface localization.

For NHE1, we observed that its overexpression led to strong signal not only at the plasma membrane but also spreaded throughout intracellular compartments, likely because of non physiological expression levels. For the K350A mutant, we did not detect an obvious qualitative difference in biochemical surface expression compared to WT (Figure 3b–c), and our functional assays indicate that the mutant behaves similarly in terms of membrane accessibility.

5. The authors may discuss if the complete lack of NHE activity on the surface was expected for the K350A variant. This may be construed as somewhat inconsistent with their model that the cytoplasmic tail is auto-inhibitory as the K350A variant is proposed to remove the auto-inhibitory interaction. I believe that the authors may argue that the surface contains inhibitory lipids, ie PI(4,5)P2; however, to what extent do the authors understand the mechanism of this inhibition? The authors might consider discussing what is known and/or speculating on this in the Discussion. To this extent, while the authors propose that PI(4,5)P2 inactivates NHE6 on the plasma membrane (Fig 3),

could they test this functionally with inhibitory lipids and with drugs that may inhibit the formation and/or localization of these lipids?

We thought the “cleanest” experiment was to show if we could activate NHE6 WT by PIP_2 removal, with our assumption that the lack of measured activity by SSM was that the PI(4,5)P2 lipids coming from the crude brain lipids (used in making the liposomes) were binding to the CTD of NHE6 WT and inhibiting its activity.

Satisfactorily, we now show that we can measure K^+-induced currents for NHE6 WT after incubation of NHE6 WT liposomes with phosphilapse C. Moreover, the K^+ affinties are much higher than than we measured for the K351A mutant, which indicates that PIP2 was probably still partially inhibiting the mutant too, i.e., consistemt with the reduced currents meaured upon addition of PIP_2 to the K351A mutant. We also show that the addition of PI3P after PLC treatment does not reduce, but enhances activity, which is consistent with the separate PI3P binding site.

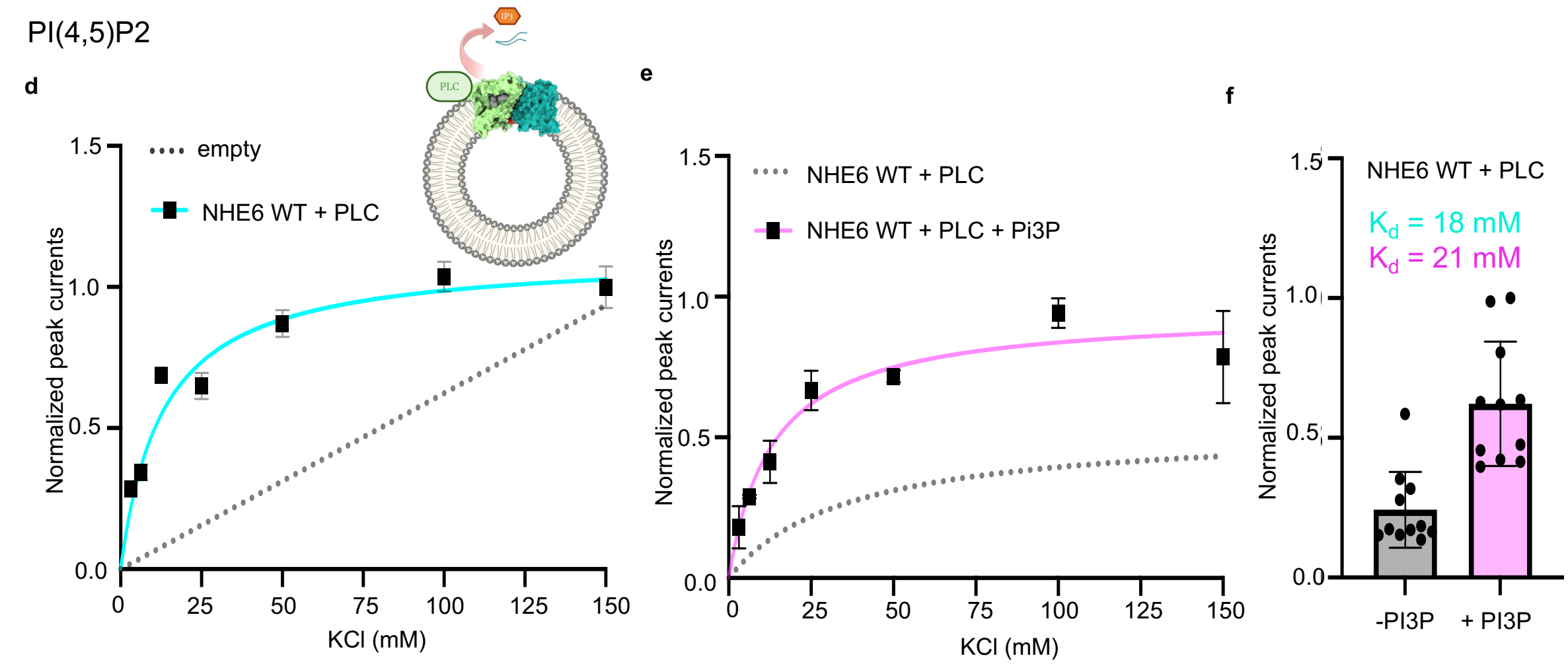

6. In Fig 3d, would the authors consider doing the endosomal pH measurement with and without PIP inhibitors to demonstrate the functional effect of these lipids on NHE6 activity?

We now show that the addition of inhbitors that block the formation of either PI(3,5)P2 or PI3P lipids reduced the pH of endosomes to that of empty vesicles (EV). Since these lipids are required for proper endosomal trafficking I think it only indirectly supports our observations, but at least it supports that the pH differences observed aredue to NHE6 expression.

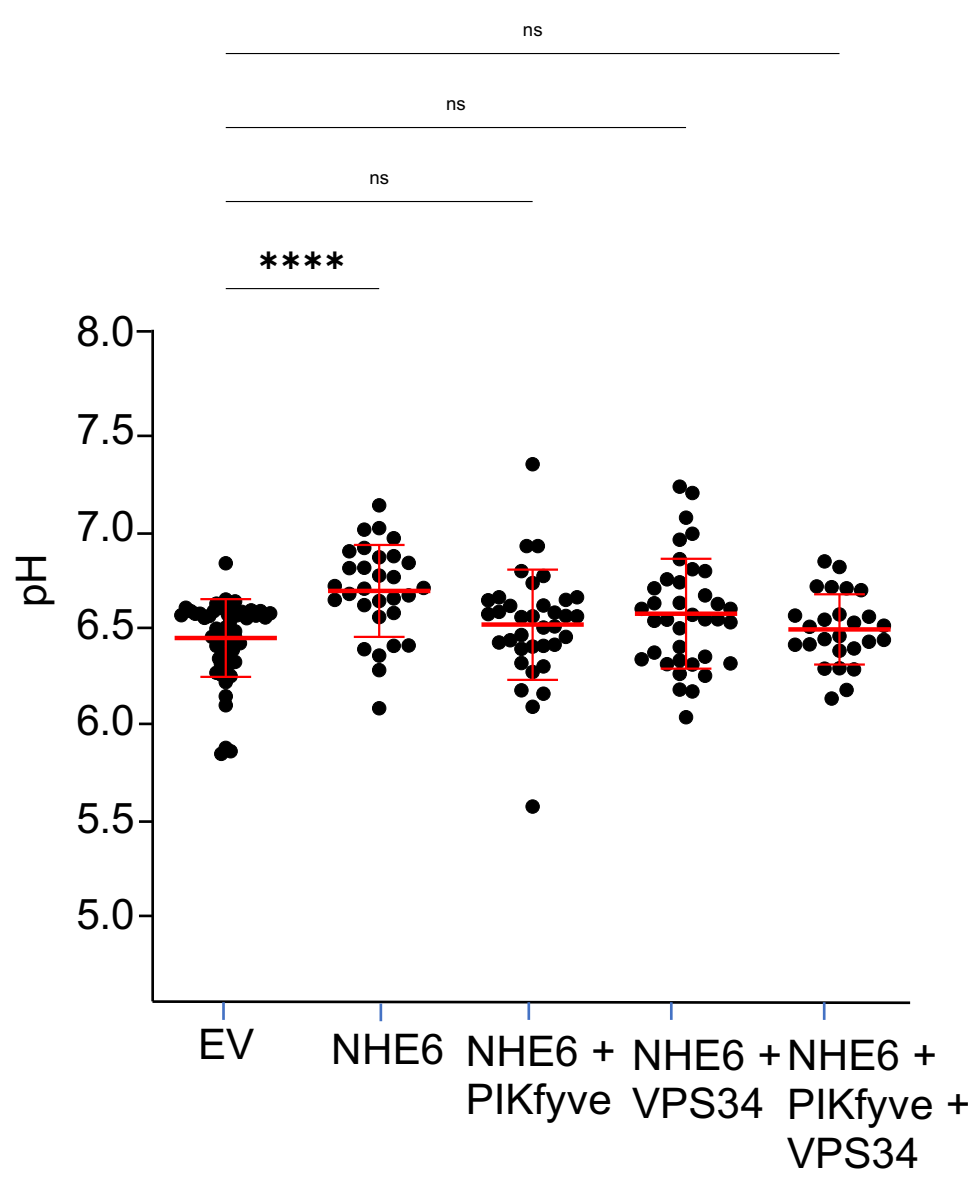

7. In Figure 3e, will be WT less active in the presence of PI(4,5)P2? The authors may consider a control whereby they test whether PI(4,5)P2 inactivates wild-type NHE6.

There is a slight shift in the background slope, but otherwise WT NHE6 is **inactive** compared to empty. Its possible that if we don't add brain lipids during purification of NHE6 WT we might activity, but we think these experiments are not needed as the PLC is a cleaner experiment.

8. Even though PI3P and PI(3,5)P2 stabilizes the NHE6 dimer in Figure 1f, the authors suggest that they have the opposite roles on regulating NHE6 in Figure 4f. Is there a discrepancy here? Please could the authors put a comment on this in the discussion, to the extent that they understand this biochemistry, about structural stabilization and functional impacts should be included.

This is an excellent point and highlights just how difficult these experiments are. The GFP-thermostability assay can measure ligand binding, but it cant distinguigh were the ligand-like lipids actually bind. From a structural viewpoint, the PI(4,5)P2 lipid is as good as you probably are going to get in cryo EM structures and certainly one of the best PI(4,5)P2 lipid densities I have seen to date. The PI3P lipid density is ambigious and we have had to infer this lipid from the fact that NHE6 co-purifies with PI3P (as measured by native MS), the high concentration of PI3P lipids found in early endosomes and for the fact that PI3P addition caused rearragment in TM1 helices, which also coordinated the PI3P lipid. We can say for certain that the dimer interface isnt a good PI(4,5)P2 lipid site as it lacks enough positively charged residues to bind such a negatively charged lipid.

In response to **Q2** we have already outlined why the PI(4,5)P_2 site isnt sufficient enough to attract PI3P lipid binding. Certainly, the PI(4,5)P_2 site would not support POPC binding and we have included some additional experiments in response to **Q2** to show that POPC still destablised the NHE6 protein in the presence of PI(4,5)P_2. The simplest interpretation is that POPC can compete out the PI3P lipid site. Thus, in addition with the functional meaurements that show very different reponses to PI3P vs PI(4,5)P_2 lipid addition, overall, we think the

evidence for separate phosphoinosital lipid binding sites are quite strong. This has been clarified in the paper and summarized below.

"In an effort to distinguish between PI3P and PI(4,5)P2 lipid binding sites we repeated the GFP-thermostability assay by adding either PI3P or POPC lipids together with the PI(4,5)P2 lipids. Whilst PI3P afforded no more thermostability than the addition of PI(4,5)P2 on its own, the addition of POPC was destabilizing (Supplementary Fig. 8b). PI3P is unlikely to bind tightly to the PI(4,5)P_2 site given that it was not observed in the CTD in any of the structures, including the D293A structure, despite the fact that synthetic PI3P lipids were added prior to blotting, i.e, PI3P concentrations would be several magnitudes higher than PI(4,5)P_2 lipids. Furthermore, only PI(4,5)P_2 lipids addition to purified *rat* NHE6 decreased Na^+(K^+)-induced peak currents (Fig. 4c-d). Since PI3P doesn't clearly bind to the CTD, it reasonable to assume that POPC would even bind more poorly and so we interpret the reduced stability as competition of POPC for the PI3P lipid binding site."

Minor comments:

1. The following sentence is not so clearly written: "The unusual repositioning of TM1 weakens the interface surface area of the homodimer from 1500 A2 in NHE9 to only 1100 A2 in NHE6 (Methods)." I believe I understand what the authors mean here, but I wonder if they broke down the concepts here into two sentences, the information may be expressed more clearly.

   Thank you, we agree. The sentence has modified for clarity.

2. Not sure Figure 7 schematics was introduced in the text.
   Thank you. This has beeen incoporated.

3. No supplementary figure 2a was found for showing the variation of CTD among NHE isoforms.
   Thank you. In an earlier version we had the sequence aligment as a seperate panel. It should be just Supplementary Fig.2, which is the sequence alignement. I think its clear that there is poor sequence conservation for the CTD.

4. Line colors for Fig 1e are not visibly distinguishable, especially between samples with brain lipids vs. samples with e.coli polar lipids.
   These colours have been selected to be compatable with color blindness. We think its still clear to distingiush between the different lipids.

5. In figure 3e-g, it is confusing that WT was tested with PI3P from brain lipids, while K351A was tested with brain lipids. Was the peak current of K351A different from WT in the presence of PI3P?
6.

   Apologies for the confusion. Both WT and K351A were tested in liposomes made from brain lipids. Since we didn't see any activity for NHE6 WT in brain liposmes we also tested if it made a difference to add PI3P lipids to NHE6 WT prior to reconstution into

the liposomes made from brain lipids (Fig. 3e). As seen in Fig. 3e the Na^+-induced currents are just background levels as they are not higher than the empty liposome control. This is just one of many meausremets for WT NHE6 spanning some 3 years of failure (!!). The key to achieve NHE6 WT was to incubate the proteoliposomes with PLC.

Yes, we tested the addition of PI3P to the K351A mutant (Fig. 4c-d) as well as PI3P addition after PLC treatment of the NHE6 WT in proteoliposomes (Fig. 6e-f). In both cases the PI3P increases the peak current signal, although not the ion-binding affinity. Since we cannot directly measure turnover with SSM-based electrophysiology we cannot say for certain this is because of faster rates, but this would be the most straightforward interpretation. Its also possible that "only" NHE6 molecules that retain some PI3P lipid in liposomes give rise to the observed SSM-based peak currents and so when we add PI3P to the purified protein we don't change the measurable ion affinity, but just the number of NHE6 molecules responding to the ion. Certainly PI3P addition produces a different response to either PI(4,5)P2 or PI(3,5)P2 addition.

We havent added the speculation about PI(3,5)P2 lipids to the paper, but my hunch is that PI(3,5)P2 could inhibit NHE6 in late endosomes where in contrast NHE9 is more active with PI(3,5)P2 lipids. These experiments might take a few more years to tease out and the speculation may better serve in a review.

7. PIP2 vs. PI(4,5)P2 seems interchangeably used, but it is unclear throughout the text and figure legends, and PI(3,5)P2 are distinct.

   Thank you. We have now updated the manuscript to specifally refer to the exact PIP_2 lipid type where appropriate to do so.

Reviewer #2 (Remarks to the Author):

In general, the structural work is careful, and the lipid-regulation model is intriguing. To fully support the mechanistic conclusions, several additional experiments would strengthen the paper, as outlined below.

1.Lipid-dependent regulation claims (PI3P at the dimer interface; PIP2 at the CTD): The central claim is that PI3P stabilizes the dimer interface and enhances activity, whereas PIP2 engages the CTD to enforce auto-inhibition and silence NHE6 at the plasma membrane. Much of this rests on density assignments and indirect functional readouts. Suggestions: (1) Reconstitute into liposomes with lipid compositions (±PI3P, ±PI(3,5)P2, ±PIP2; ±cholesterol) and quantify functional activity. (2) Mutate key contacts at the interface (e.g., N74, W371/β-hairpin Lys) and at the CTD–core latch (e.g., K351 and basic residues near the modeled PIP2 pocket) and test effects on stability and transport. (3) Provide local map-model validation around the lipid densities.

We have avoided the reconsitutions into different lipid compositions as we would then need to work with synthetic lipid preperations and, in our experience, this could take several years to optimise (Nat Comms: 14: 4070 (2023). Rather, where ever possible, we have added the synthetic PI3P *vs* PI(4,5)P2 lipid to the purified NHE6 protein prior to reconsitution into liposomes, which were made with the same brain lipids.

In terms of mutants, we have already shown than NHE6 WT is inactive in liposomes made with brain lipids whereas NHE6 K351A is active. The interpretation is PI(4,5)P2 coming from crude brain lipids can stabilise the auto-inhibited state in the NHE6 WT but not for the K351A mutant with a more flexible CTD.

The "cleanest" experiment we could think was show if we remove PI(4,5)P_2 in the brain lipids used for SSM-based electrophysiology measurements we could activate NHE6 WT. Thus, by incubating with phospholipase C we could "finally" obtain K^+-induced currents for NHE6 WT. Further, by adding PI3P addition after PLC we increase the K^+ induced signals for the NHE6 WT protein too.

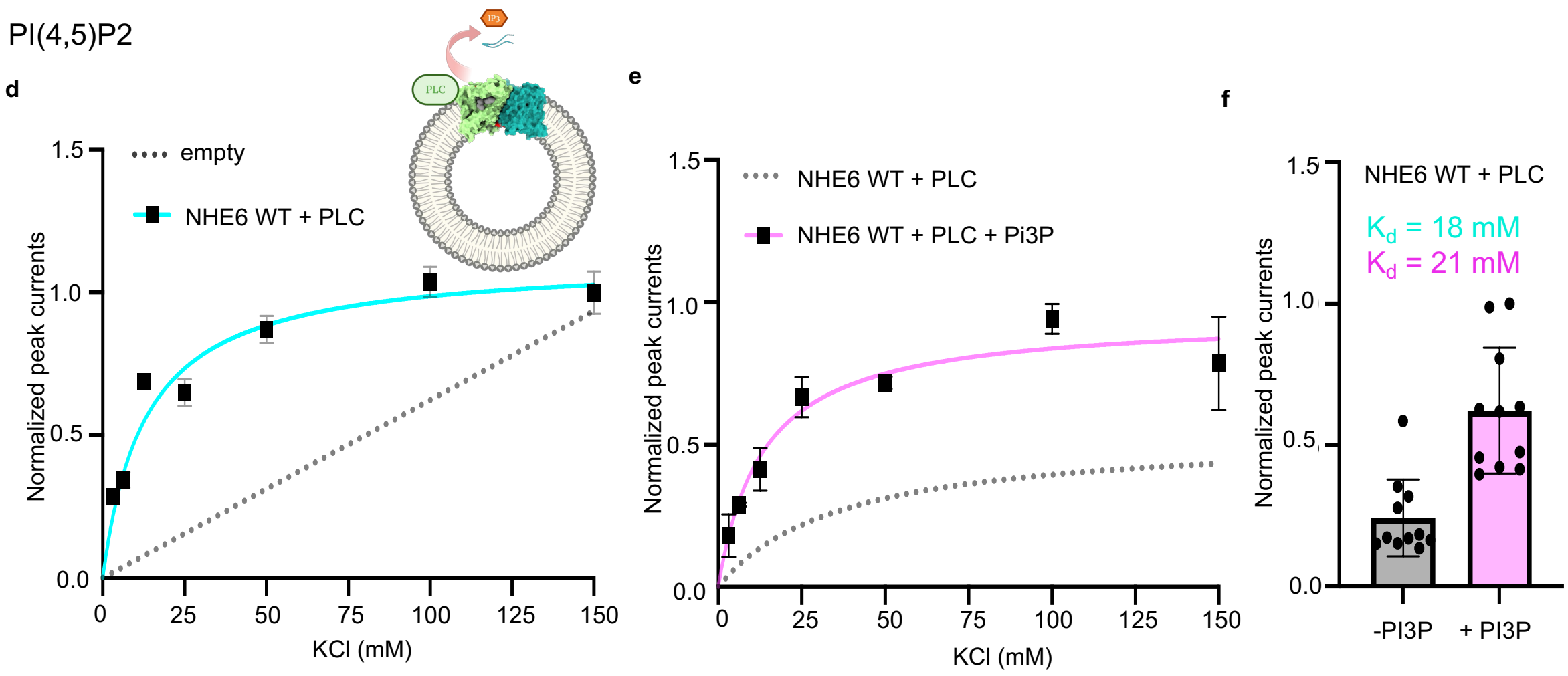

The density for PI(4,5)P_2 is exquisite. Please look at the maps and structures provided with our submission. We have also incldued the resolution estimate for NHE6 WT in detergent, which has local resolution estimates for the lipid < 3Å. Regarding PIP_2 vs PI3P lipid binding sites please see the responses to **Q2** and **Q8** to Referee 1.

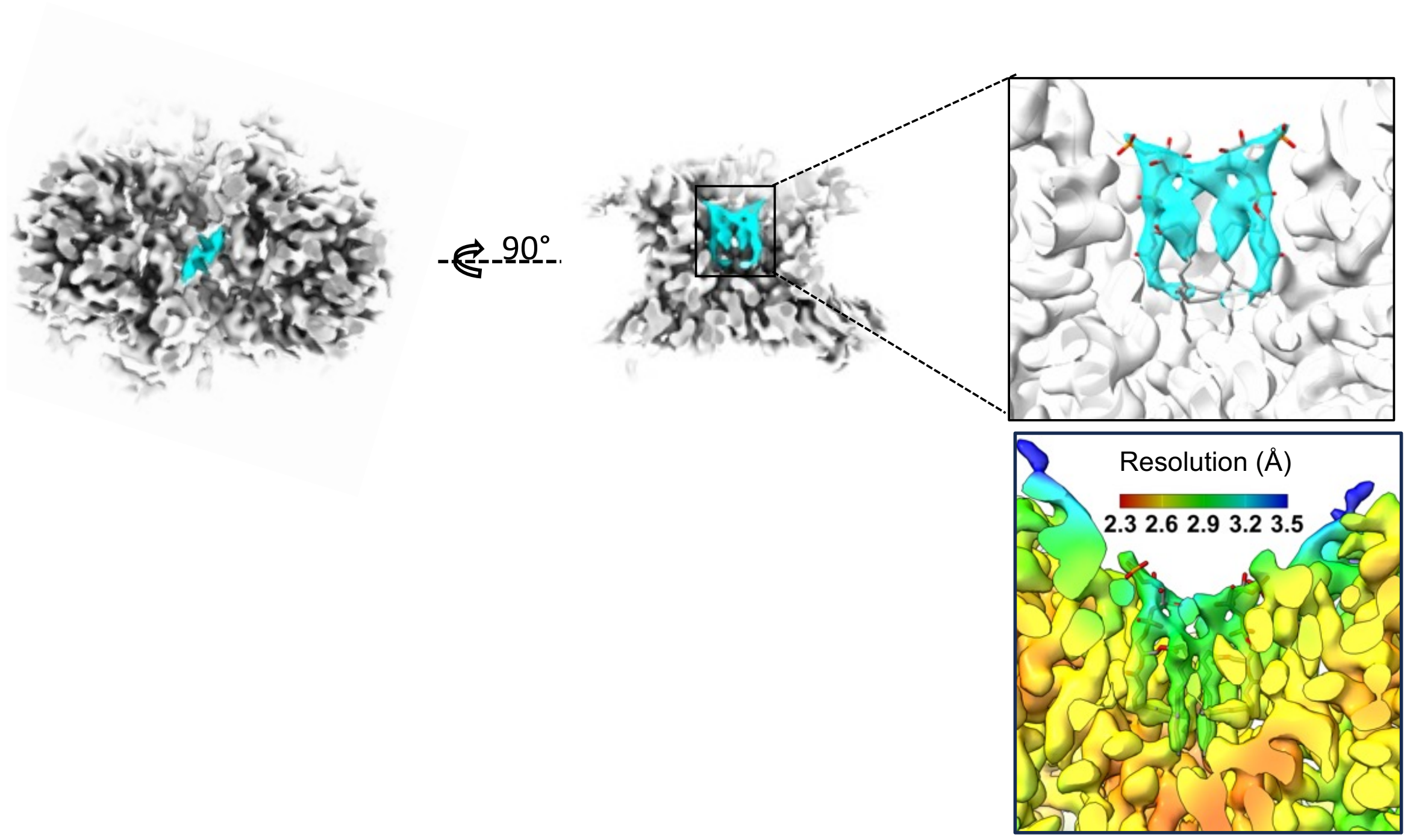

2.Ion-binding site identity and evidence: The Na^+ site is modeled with supportive data (D293A abolishes the extra density), but chemical identity/occupancy is still inferred. Suggest doing a contour analysis to rule out that the density is water.

Im not sure contour analysis would help to distinguish between water and a metal ion. We have been working close to 20 years on Na^+/H^+ exhangers and have obtained high resolution crystal structures at 2Å resolution (e.g., Nat Struct Mol Biol 23:248-55) and yet, in this family of transporters, we have never seen any "waters" next to the strictly conserved asparatate residue.

This NHE6 map quality is excellent and the Na^+ ion position makes a lot of sense based on MD simulations of NapA, NHE9 and structures of KefC. Indeed, the Na^+ ion is a close match to the predicted Na^+ position by AlphaFold 3, which is we now included in the paper.
3.Na^+ vs K^+ selectivity and kinetics: SSM measurements rely primarily on K351A and suggest similar millimolar Kd's for Na^+ and K^+ with PI3P-dependent enhancement. It is concluded that K^+ is the transported ion. It would be more physiologic to perform Na^+-K^+ competition studies by measuring the dose response to K^+ in the presence of physiologic Na^+ and vise versa since they compete. These data would provide the effective Kd for each ion. I would also add inhibitor controls (amiloride-class) to confirm NHE-specific exchange in the reconstitution used for SSM.

We have calculated the K_d for Na^+ and the K_d for K^+ under the same experimental conditions seperately. This is standard practice in the transport field for obvious reasons. One can measure substrate affinities in the presene of competing substrate (which is either a IC_{50} or a Ki depending on the relative concentrations), but the IC_{50} estimates are typically similar to the Kd estimated. Given the relativley higher concentration of K^+ vs Na^+ in the cytoplasm then there is a higher probablity for K^+ to bind. Inhibitor experiments are complicated as we would need to have a similar compound that doesn't inhibit in the non-activating buffer to control for any artefacts caused by introducing the compound. It seems that the amiloride-class inhbitors do not work on the endosomal NHEs, like NHE6. I think the data we have is already convincing that the peak SSM signals come from NHE6 functional activity, which was already clear by the poor responses seen in liposomes made from *E. coli* and *yeast lipids* vs *brain lipids*.

4.CTD auto-inhibition: correlation vs causation: Structures and cell data argue the CTD latches onto TM7 (K351) and that PIP2 stabilizes an off-state; however, direct functional causality needs clearer results. Would test ΔC1/point mutants at the CTD–TM7 interface (e.g., L581/L582↔TM7 Lys) for effects on activity and conformational bias.

While we could make many further mutations, we think the lysine mutant is already sufficient to make the point that the CTD is interacting with TM7. Also we have been able to build more of the CTD in structures where we have lipids interacting with the transporter. One mutation is enough to fully detach the CTD, based on the fact that i) it cant be modelled in the maps and ii) this protein was producing currents when reconsituted into brain lipids. The interaction between TM7 and a charged residue is highly conserved interaction (as seen by AF2 structures for all NHE members) and it was also seen in the volatge-gated member SLC9C1 (Nature 623:193–201 (2023).

Since we now record NHE6 WT activity after PLC addition, then its also clear that the $PI(4,5)P_2$ lipid was inhibiting the protein and further the C-terminal tail auto-inhibits the protein.

5.Plasma-membrane inactivity experiments: Consider also depletion of plasma membrane PIP2 as a control.

We think the PLC addition in the in vitro proteoliposome SSM-based electrophysiology assays effectively depletes for PI(4,5)P_2 lipids and shows that its removal now activate NHE6 WT.

6. The pH-responsive pHrodo™ Red transferrin endosomal pH measurement: The resolution of the assay seems poor given the small changes in the ratio between pH 7.0 and 4.5. Other than these extremes, how accurate is the measurement?

pH is a log scale. A change of just 0.2 pH units is enough to arrest cultured fibroblast cells in the Go phase of the cell cycle. The changes we see here are robust and significant. To help support this claim we now show if we add inhibitors of either PI3P or PI(3,5)P2 formation that would disrupt NHE6 traffacking and activity that the pH changes decrease to background (EV levels.

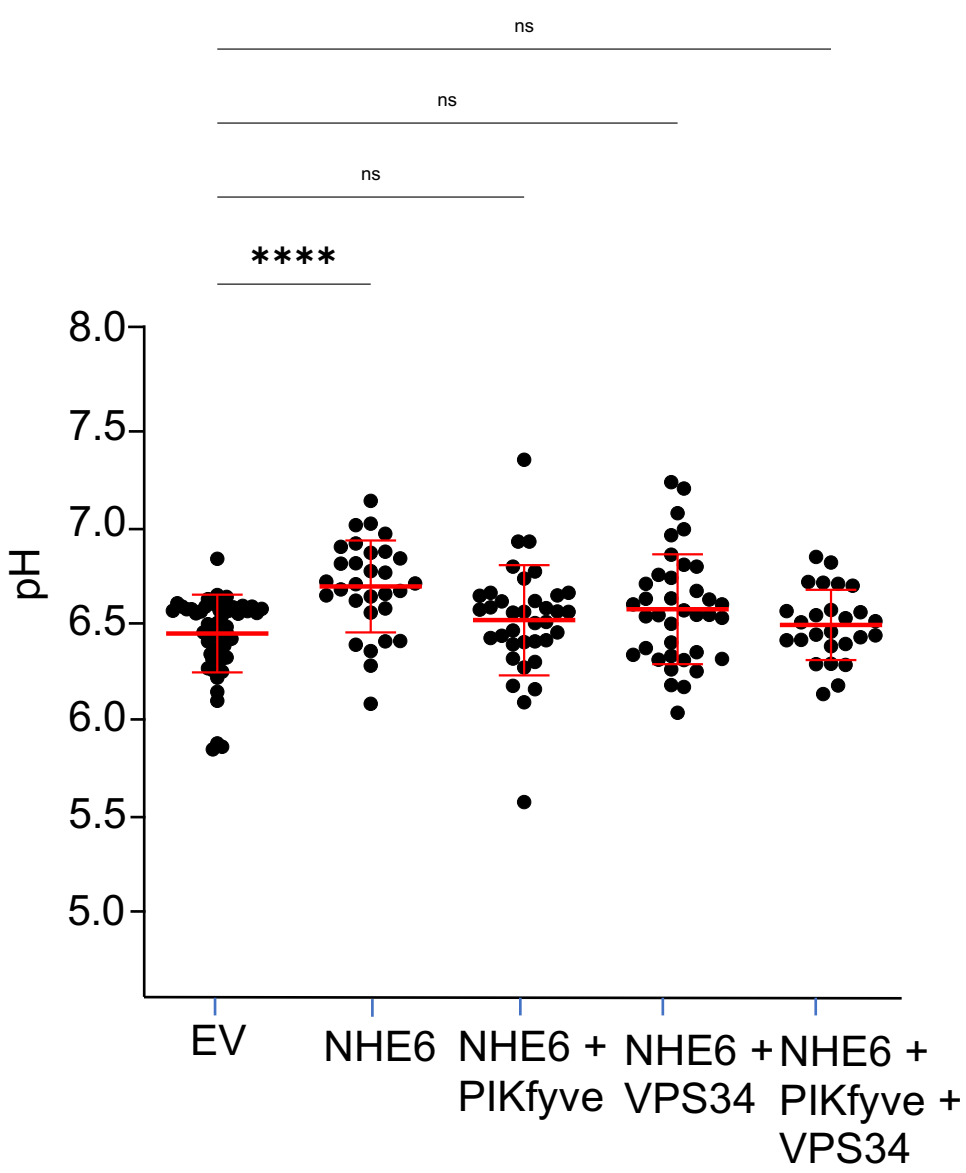

Reviewer #3 (Remarks to the Author):

I co-reviewed this manuscript with one of the reviewers who provided the listed reports. This is part of the Nature Communications initiative to facilitate training in peer review and to provide appropriate recognition for Early Career Researchers who co-review manuscripts.

Reviewer #4 (Remarks to the Author):

I co-reviewed this manuscript with one of the reviewers who provided the listed reports. This is part of the Nature Communications initiative to facilitate training in peer review and to provide appropriate recognition for Early Career Researchers who co-review manuscripts.

Reviewer #5 (Remarks to the Author):

- What are the noteworthy results?

The manuscript includes one figure (S3) with native mass spectrometry data and lipidomics work. The portion of the mass spectrum shown shows the signals for NHE6 dimer overlapping with HSP70 dimer. HSP70 was identified by MSMS analysis of the HSP70 monomer (shown inset). The NHE6 protein did not need to be pure for cryo-EM as other proteins can be ignored in the analysis. The lipidomics is adequate.

OK

- Will the work be of significance to the field and related fields? How does it compare to the established literature? If the work is not original, please provide relevant references.
The work is of some significance to the analysis but placement in supplementary data is probably adequate.

OK

- Does the work support the conclusions and claims, or is additional evidence needed?
The work supports the conclusions made in the manuscript.

OK

- Are there any flaws in the data analysis, interpretation and conclusions? - Do these prohibit publication or require revision?
The data analysis is not flawed with acceptable interpretation and conclusions.

OK

- Is the methodology sound? Does the work meet the expected standards in your field?
The methodology is sound and meets the expected standards of the field.

OK

- Is there enough detail provided in the methods for the work to be reproduced?
There is adequate detail in the methods. The origin of the time values in the top right of part D is unclear – they can be removed.

OK, this has been removed.

However, the authors did not perform the additional interface-testing experiments that were suggested, and the rebuttal explicitly argues that one mutation is sufficient. Since CTD-based inhibition is one of the principal mechanistic conclusions of the paper, the remaining point is that the current data support a CTD-linked inhibitory model, but do not yet fully establish the precise CTD-TM7 inhibitory mechanism with the degree of certainty currently claimed.

Referee 2 suggested that we generate additional mutants to further probe the interaction between TM7 and ICH1. In our resubmission, we should have more clearly justified why we consider the single K351A mutation to be sufficient for the proposed model.

The key point is that the interaction between TM7 and ICH1 involves the side chain of K351 engaging primarily with backbone atoms at the breakpoint of ICH1 and ICH3. As this represents the only direct interaction between TM7 and ICH1/ICH3, mutations within either ICH1 or ICH3 are unlikely to directly perturb this interface, since they would not alter the backbone interactions that mediate the contact.

To clarify this, we have made a new main figure shown below to better show the interaction between the CTD and the transporter module TM7 in the rat NHE6 structure in detergent.

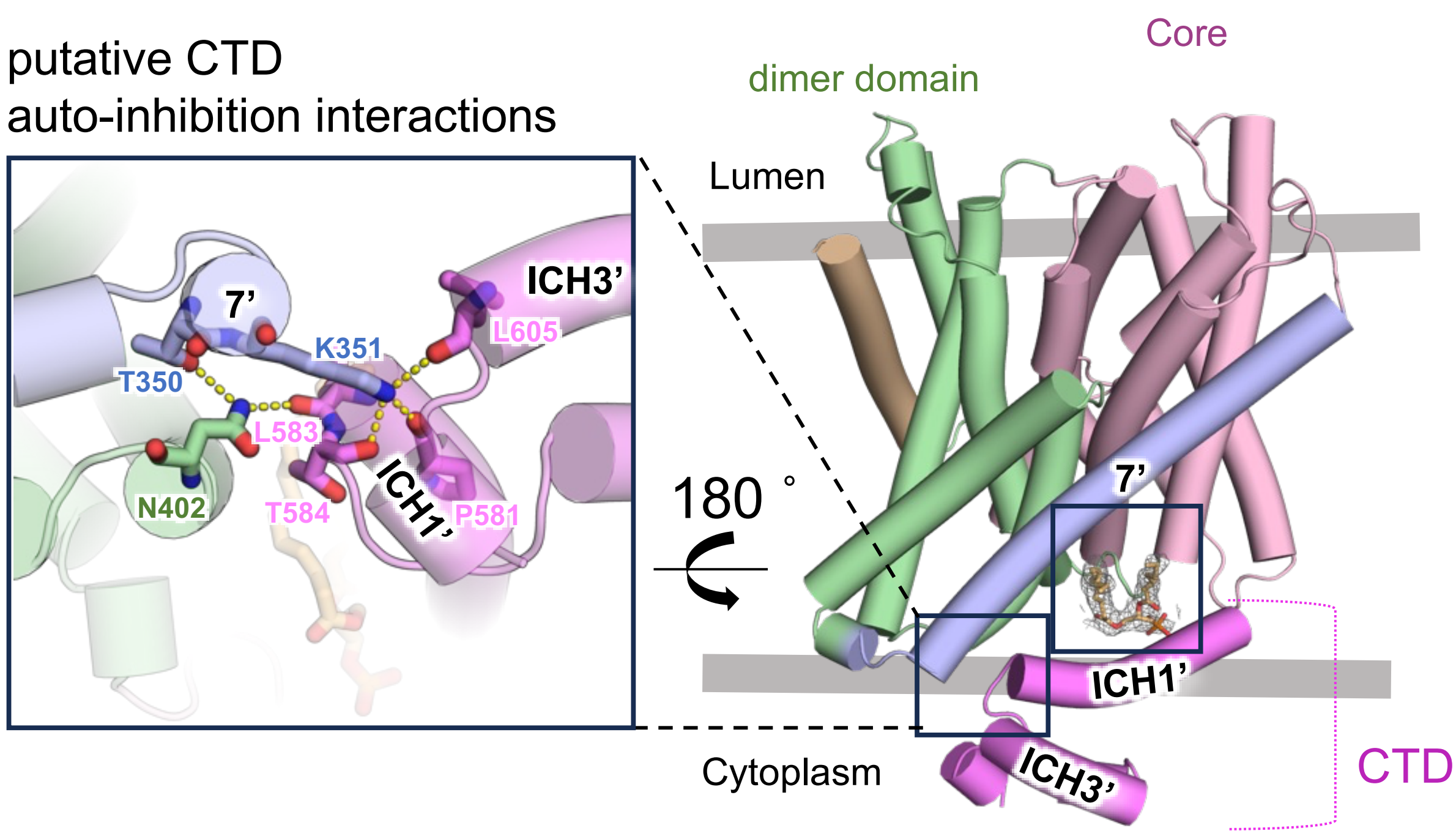

We have also made a new Supplementary Figure 4. This figure shows that the same TM7–ICH1 interaction (as was also observed in the experimental NHE9 structure; Nat Comms: 16: 3055 (2025) is also consistently predicted by AlphaFold3 across all NHE family members.

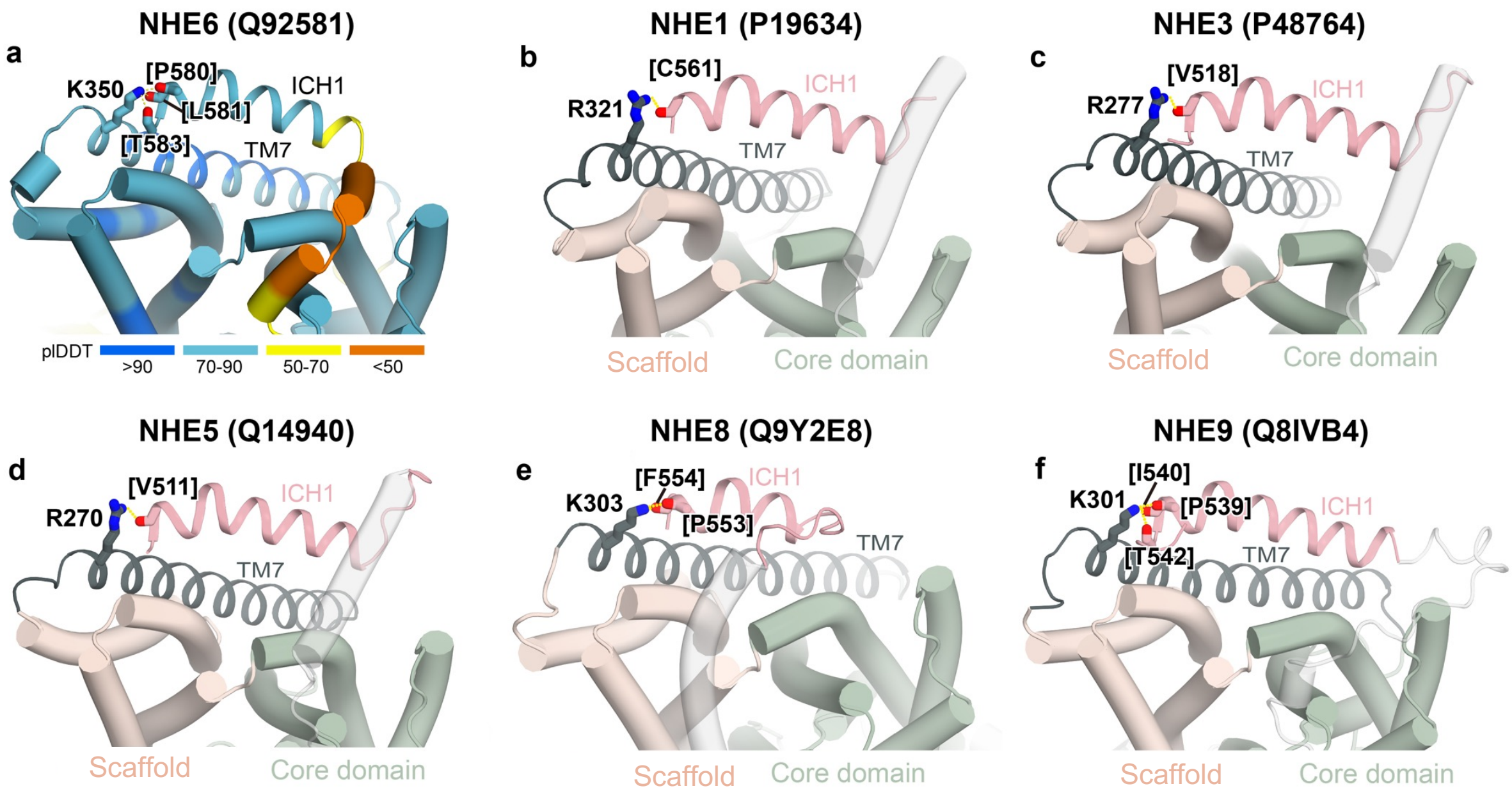

While it remains formally possible that mutations in ICH1 could indirectly influence this interaction—for example, by altering the mobility of the helix—we consider this unlikely. Furthermore, ICH1-ICH3 already appears to be highly flexible; indeed, we are unable to model the loop connecting the end of TM13 to the start of ICH1. This suggests that ICH1-ICH3 is only weakly tethered and already samples a high degree of conformational freedom. We think this flexibility is important as it allows the CTD to regulate ativity also by lipid interactions, like PIP_2. To highlight the CTD is flexible (in addition to Fig. 2 that shows the different positions of ICH1 with and wihout lipids bound) we have made another figure (now Supplementary Fig. 2b and shown below) to complement the multiple sequence alignment and topology to clarify the loop located between TM13 and the start of ICH1 that could not be modelled.

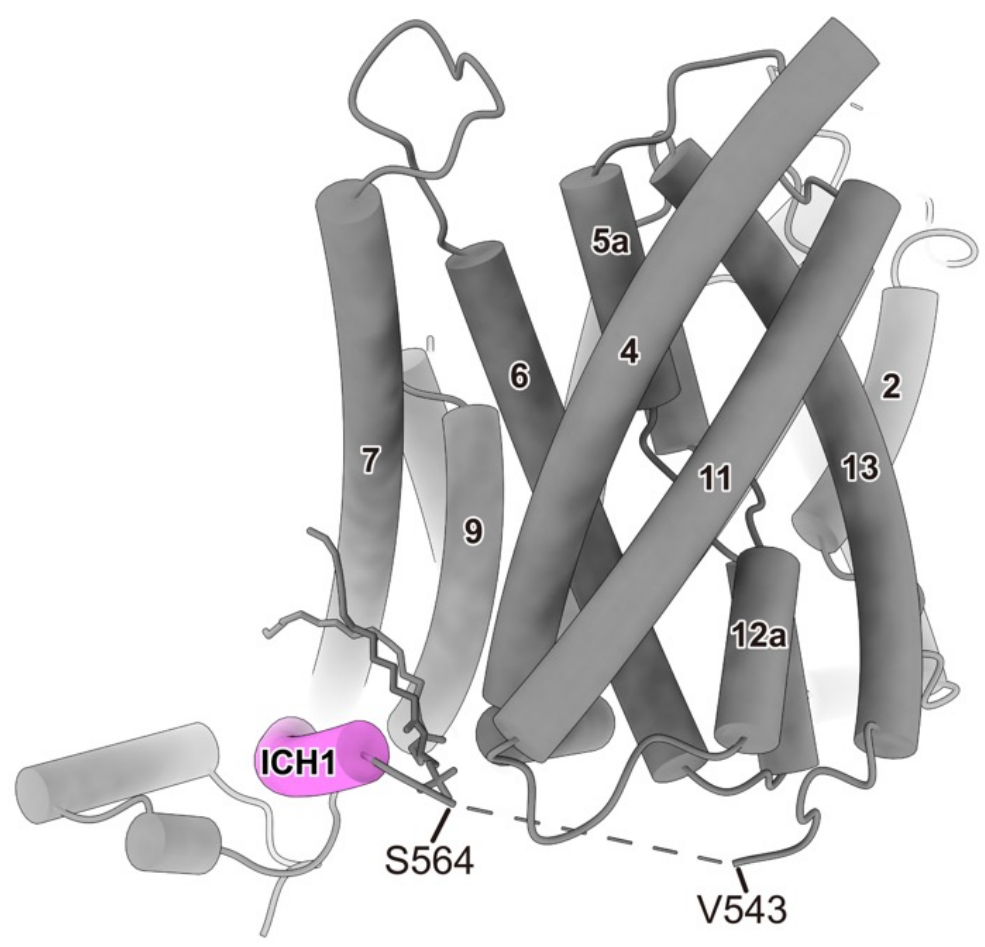

Lastly, instead of introducing point mutations within ICH1 that we think will be too subtle to interpret with our current in vitro analysis (since the TM7 forms interactions with ICH1-ICH3 backbone atoms), we have generated a construct lacking the entire CTD of NHE6. However, this truncation mutant expression levels were considerably lower and could not be purified; in the absence of the CTD, the protein might be too dynamic leading to lower yeilds and degradation.

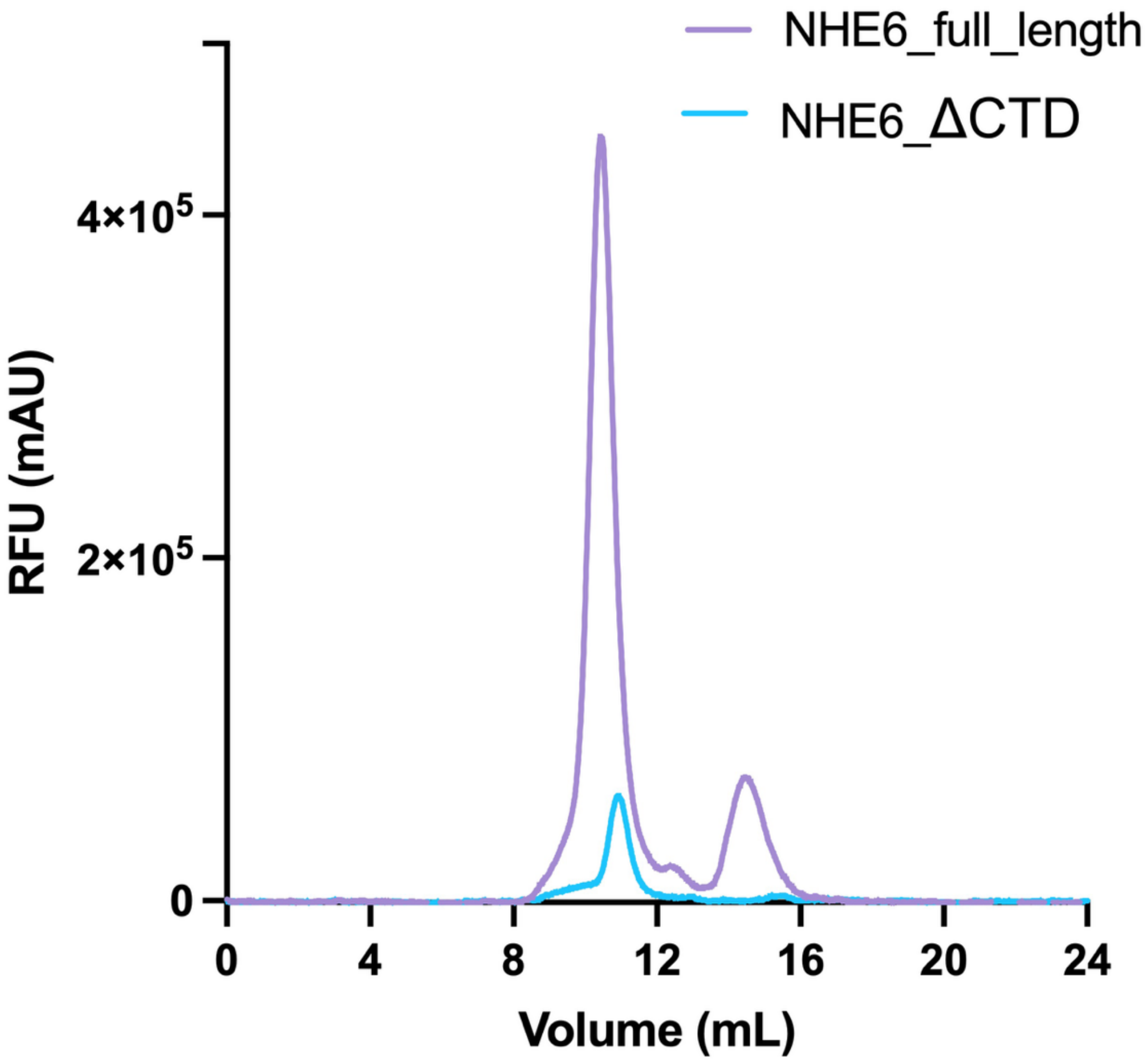

In summary, we think our data is sufficient to support that the proposed auto-inhibitory interaction is located between the charged TM7 residue and the backbone ICH1-ICH3 residues. I think its also clear that this CTD is still flexible and hence the sensitivity of CTD auto-inhbition by specific lipids as seen by cryo EM studies and SSM-based electrophysiology. We don't think we have over-claimed our model, but we think its clear that this is just the "framework" from which we could build upon with futher studies. Rather than ICH1-ICH3 mutations and in vitro analysis, we will rather focus on phophorylation mapping and FRET based traffciking studies as what we are really interested to understand in vivo CTD dynamics. We this point is clear in the discussion, but let us know if you think this needs to be more explicitiy made.

Reviewer #2 (Remarks to the Author):

1. Lipid-dependent regulation: This point is significantly improved. The authors now provide better support that PI3P and PI(4,5)P2 have distinct functional effects. PI3P supports activity, whereas PI(4,5)P2 inhibits activity. The authors also added thermostability data and argue that PI3P does not bind tightly to the CTD PI(4,5)P2 site because PI3P was not observed there despite being added before blotting at high concentration. The PLC experiment is particularly important because it functionally connects PI(4,5)P2 removal with rescue of WT NHE6 activity. This is much stronger than relying only on static cryo-EM lipid density. The authors still did not perform defined lipid reconstitution experiments with controlled lipid compositions or mutagenesis of proposed lipid-contact residues. The lipid-regulation model is now much better supported, especially the inhibitory role of PI(4,5)P2. However, the precise lipid-specific mechanism remains incompletely tested at the residue level.

We chose brain lipids for our reconstitution experiments because NHE6 is highly expressed in the brain and therefore operates in a native lipid environment enriched in phosphoinositides. Using this system, we demonstrate that addition of synthetic $PI(4,5)P_2$ to purified NHE6 prior to reconstitution strongly inhibits ion-induced currents. In contrast, addition of synthetic PI3P enhances ion-induced currents. Importantly, we also performed the reciprocal experiment: PLC treatment of brain lipid liposomes activates WT NHE6, and subsequent addition of PI3P further increases activity. Together, these complementary gain- and loss-of-function experiments provide strong evidence that $PI(4,5)P_2$ and PI3P exert opposing regulatory effects on NHE6 activity.

These functional observations are supported by several independent structural and biophysical findings. The cryo-EM structure reveals a highly defined $PI(4,5)P_2$-binding site located at the interface between the C-terminal regulatory domain and the transporter domain, providing a mechanistic basis for the inhibitory effect of $PI(4,5)P_2$. Consistent with lipid specificity, PI3P was not observed at this site even when added at high concentration prior to grid preparation. Furthermore, the density associated with PI3P localizes to the dimerization interface. Although this density is less well resolved than that of $PI(4,5)P_2$, it coincides with lipid-binding regions that have been implicated in stabilization of the dimeric active state in other Na^+/H^+ exchangers. In addition, native mass spectrometry demonstrates that purified NHE6 co-purifies with lipids whose masses are consistent with PI3P and $PI(4,5)P_2$, supporting the physiological relevance of these interactions.

While additional mutagenesis or reconstitution into fully defined lipid mixtures could potentially provide further insight, it is not clear that such experiments would allow substantially stronger conclusions. Reconstitution into simplified lipid compositions introduces significant challenges, including variable reconstitution efficiency and potential loss of protein stability arising from the absence of native stabilizing lipids. Likewise, although mutagenesis can be informative, interpretation is complicated by the possibility that mutations alter protein structure, stability, or conformational equilibria independently of lipid binding. Rigorous interpretation would therefore require structural characterization of each of the individual mutants.

In our view, the PLC treatment experiments we have shown provide a particularly clean and minimally perturbative approach, as they modulate endogenous phosphoinositides within an

otherwise native-like membrane environment without introducing potentially confounding structural alterations to the protein itself.

2. Na^+ versus K^+ physiological relevance: The authors now provide separate apparent affinity measurements for Na^+ and K^+ and argue that K^+ is more likely to be the physiologic counter-ion because intracellular/endosomal-facing K^+ concentration is much higher than Na^+. This is a reasonable interpretation, and the manuscript now frames the argument more clearly. The authors still did not perform Na^+/K^+ competition experiments under mixed-ion conditions. This is important because, in vivo, Na^+ and K^+ are not presented to the transporter separately. They compete in the same intracellular/endosomal cytoplasmic environment. The rebuttal says separate Kd measurements are standard and that competition experiments would provide IC50/Ki-type information, but this does not fully answer the physiological competition question. The data support NHE6 transport signals for both Na^+ and K^+ and make K^+ a plausible physiologic counter-ion. However, the conclusion that K^+ is the main physiological ion remains inferential rather than directly proven by mixed-ion competition experiments.

We agree that mixed-ion competition experiments can be informative in transport systems where different ions bind to distinct sites or exert allosteric effects on transport. However, $Na^+(K^+)/H^+$ exchangers operate through a ping-pong exchange mechanism in which Na^+ and K^+ compete for the same ion-binding site. Under these conditions, the physiologically relevant transported ion is determined by the combination of ion affinities and the relative ion concentrations (see Drew and Boudker, Nature 2024, "Ion and lipid orchestration of secondary active transport").

In endosomes, the inwardly directed H^+ gradient established by the V-type ATPase provides the driving force for cation uptake from the cytoplasm into the endosomal lumen. Under physiological conditions, cytoplasmic K^+ concentrations are approximately tenfold higher than Na^+ concentrations. Combined with our observation that NHE6 exhibits an approximately twofold higher apparent affinity for K^+ than for Na^+, these conditions strongly favor K^+ binding and transport. We therefore conclude that K^+ is likely to be the predominant physiological counter-ion for NHE6 in endosomes.

We acknowledge that mixed-ion competition experiments could provide an additional quantitative assessment of the relative contributions of Na^+ and K^+ under defined conditions. However, because both ions compete for the same binding site, such experiments would primarily reflect the combined effects of affinity and concentration that are already captured by the measured apparent affinities and the known physiological ion gradients. For this reason, we believe that the current data provide a robust basis for identifying K^+ as the most likely physiological counter-ion, while recognizing that Na^+ could also serve as a substrate under conditions where its concentration becomes sufficiently elevated in respect to K^+.

An interesting implication of these findings is that NHE6 could potentially mediate K^+/Na^+ exchange when present at the plasma membrane. In this context, the complex lipid-dependent regulatory mechanisms described in this study may help prevent inappropriate transporter activity outside the endosomal system. Given the rapid recycling of membrane proteins between endosomes and the plasma membrane, such regulation may be particularly important for maintaining compartment-specific transport activity.

3. CTD auto-inhibition: This point is also significantly improved. The K351A mutant, loss of modeled CTD density in that mutant, activity of K351A in brain-lipid liposomes, and now PLC activation of WT NHE6 together provide a much stronger case that the CTD participates in auto-inhibition and that PI(4,5)P2 stabilizes the inhibited state. The authors also clarify that K351 interacts primarily with backbone atoms at the ICH1/ICH3 breakpoint, and they added a figure to better show the TM7–ICH1/ICH3 interaction. The authors still did not successfully provide additional functional tests of the CTD interface. They argue that K351A is sufficient because K351 is the main TM7 contact and because mutations in ICH1/ICH3 side chains might not disrupt a backbone-mediated interaction. They also state that a CTD deletion construct expressed poorly and could not be purified. That explanation is reasonable, but it does not fully replace direct reciprocal mutagenesis or a successful CTD truncation/interaction test. The CTD auto-inhibition model is now strongly supported. However, the exact CTD–TM7 interface mechanism is not exhaustively validated. It should be presented as a strong mechanistic framework, not as a completely proven residue-by-residue causal mechanism.

We agree with the referee's assessment. While our structural, biochemical, and functional data collectively provide strong support for a CTD-mediated auto-inhibitory mechanism, we do not claim that the precise residue-by-residue interactions at the CTD–TM7 interface have been exhaustively validated.

Accordingly, we have been careful to present the CTD–TM7 interaction as a mechanistic framework that is strongly supported by the available evidence, including the K351A mutant phenotype, the loss of CTD density in the K351A structure, the increased activity of K351A in brain lipid liposomes, and the activation of WT NHE6 following PLC treatment. At the same time, we recognize that additional experiments would be required to establish a complete residue-level mechanism.

To reflect this distinction, we have deliberately avoided depicting a highly detailed residue-by-residue interaction model in the final schematic. Instead, the model illustrates the broader regulatory mechanism supported by our current data while avoiding overinterpretation of specific molecular contacts.

4. Plasma-membrane inactivity: The authors added immunofluorescence/localization data and confirm that NHE6-WT partially overlaps with surface dye and strongly marks RAB5-positive early endosomes. They also rely on surface biotinylation to support plasma-membrane expression of the constructs. They also address the apparent inconsistency that the K350A mutant remains inactive at the plasma membrane by arguing that CTD displacement alone may be insufficient and that endosomal PI3P and other CTD-associated factors may also be required. They did not directly deplete plasma-membrane PI(4,5)P2 in the cell-based assay. Instead, they used PLC treatment in proteoliposomes as the cleaner biochemical test of PI(4,5)P2 removal. That is useful, but it is not exactly the same as demonstrating the mechanism in cells at the plasma membrane. The plasma-membrane inactivity model is plausible and better supported than before, but the cellular PI(4,5)P2 mechanism remains indirectly supported rather than directly demonstrated in the plasma-membrane assay.

We agree with the referee that our proposed plasma-membrane PI(4,5)P2 regulatory mechanism is supported indirectly rather than demonstrated directly in the cellular plasma-membrane assay.

A direct test of this hypothesis in cells is technically challenging. To our knowledge, there is currently no experimental approach that selectively removes plasma-membrane PI(4,5)P2 without perturbing the cellular pathways responsible for its synthesis, turnover, or downstream signaling. Manipulations that reduce PI(4,5)P2 levels generally rely on inhibition or recruitment of lipid-modifying enzymes, which inevitably affect numerous PI(4,5)P2-dependent cellular processes. Given the central role of PI(4,5)P2 in regulating a wide range of membrane proteins and signaling pathways, the interpretation of such experiments would therefore be complicated by substantial indirect effects.

For this reason, we chose to test the role of PI(4,5)P2 in a more controlled biochemical system. The PLC experiments in proteoliposomes allow selective depletion of PI(4,5)P2 while avoiding the many confounding variables present in cells. Together with the cryo-EM structure, which reveals a defined PI(4,5)P2-binding site linking the CTD to the transporter domain, and the activation of NHE6 following PI(4,5)P2 depletion, we believe these data provide strong support for the proposed mechanism.

Nevertheless, we agree that the cellular plasma-membrane inactivity model should be viewed as a mechanistic interpretation supported by multiple lines of evidence rather than as a directly demonstrated consequence of plasma-membrane PI(4,5)P2 depletion in living cells. We have therefore taken care to present this model accordingly in the revised manuscript.

5. Ion-binding-site identity: The authors provide structural and comparative arguments supporting the modeled ion-binding site, including the conserved location in the core domain and comparison to other NHE-family structures. The chemical identity of the density is still inferred. There is no direct anomalous validation, heavy-ion substitution, or functional mutagenesis of the coordinating residues specifically to validate the ion-site assignment. The ion-site assignment is reasonable, but it remains structurally inferred rather than directly validated chemically.

We agree that the identity of the modeled ion is inferred from the structural data rather than directly established through anomalous scattering. However, this is also the case for the vast majority of ion assignments in membrane protein structures, including many determined by X-ray crystallography. While approaches such as Rb^+ substitution can provide anomalous signal, they nevertheless rely on inference because Rb^+ is a surrogate ion and not identical to the physiological Na^+ or K^+ substrates.

In our case, the assignment is supported by several independent observations. First, the density is located at the conserved ion-binding site within the transporter core, where ion coordination is expected based on structural and functional studies of the NHE family. Second, the observed density is consistent with the expected coordination geometry of a bound monovalent cation. Third, when we determined the structure of an ion-binding-site mutant, the corresponding non-protein density was no longer observed, providing independent support that the feature is associated with the modeled ion-binding site.

Importantly, we do not observe similarly well-defined non-protein densities elsewhere in the structure that could plausibly correspond to ordered water molecules and therefore lead to

systematic misassignment. The modeled ion density is a distinct and prominent feature within the map. In addition, the location of the ion-binding site is consistent with the AlphaFold3 prediction. This agreement is notable because, to our knowledge, no experimentally determined Na^+-bound structure of a Na^+/H^+ exchanger was available during model training, making this an independent line of support for the assignment.

We therefore agree that the ion identity remains structurally inferred, but we believe that the combination of density, coordination geometry, conservation within the transporter family, loss of density in the ion-binding-site mutant, and agreement with AlphaFold3 provides strong evidence that the assignment is correct.

6. Disease mutations: The authors map Christianson syndrome mutations onto the structure and discuss how mutations may affect PI3P-dependent dimer stabilization, CTD regulation, or ion transport. They did not functionally test disease mutations. Therefore, the disease relevance is strengthened anatomically/structurally but not experimentally. The disease-mutation mapping is useful, but the functional consequences of specific patient variants remain untested.

We agree that our analysis of Christianson syndrome mutations is primarily structural and does not constitute a direct functional characterization of individual patient variants.

However, it is important to note that NHE6 disease mutations have been the subject of extensive investigation over many years by multiple groups. The majority are loss-of-function mutations that impair NHE6 activity and lead to endosomal overacidification, consistent with the established pathogenic mechanism underlying Christianson syndrome. In contrast, a small number of rare variants have been reported to exhibit gain-of-function behavior (e.g., Neurobiology of Disease 121, 187–204).

The primary goal of our mutation mapping was therefore not to provide a comprehensive functional analysis of disease-associated variants, but rather to place these previously identified mutations into a structural context. We believe that the structural analysis substantially strengthens the interpretation of the existing disease literature by linking previously characterized pathogenic mutations to how they may impact NHE6 activity.
